# The Acidity of Weak NH Acids: Expanding the p*K*_a_ Scale in Acetonitrile

**DOI:** 10.1021/acsorginorgau.4c00095

**Published:** 2025-03-13

**Authors:** Märt Lõkov, Carmen Kesküla, Sofja Tshepelevitsh, Marta-Lisette Pikma, Jaan Saame, Dmitri Trubitsõn, Tõnis Kanger, Ivo Leito

**Affiliations:** † Institute of Chemistry, University of Tartu, Tartu 50411, Estonia; ‡ Department of Chemistry & Biotechnology, Tallinn University of Technology, 12618 Tallinn, Estonia

**Keywords:** acidity, p*K*_a_, nitrogen heterocycles, aromatic amines, acetonitrile, gas-phase acidity, UV−vis spectroscopy

## Abstract

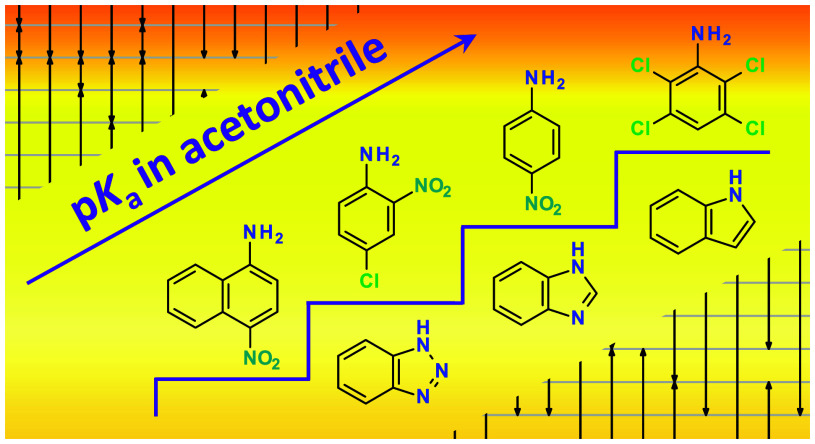

Nitrogen heterocycles and aromatic amines are well-known and widely used compounds that are usually not seen as acids, although in organic solvents like acetonitrile (MeCN) or dimethyl sulfoxide (DMSO) their acidic properties can be observed. In this work, the acidities (p*K*_a_ values) of 37 such weak NH acids were determined in acetonitrile and presented together with computational gas-phase acidities and literature p*K*_a_ values in DMSO. In the course of the work the weakest acids range (from p*K*_a_ 29 upward) of the MeCN acidity scale has been revised and expanded to around 33.5 by adding 31 compounds in that specific region and the span of experimental p*K*_a_ values in MeCN is now more than 30 orders of magnitude. The relations between the structure and acidity of a selection of the studied compounds have been investigated in MeCN and DMSO. The measured p*K*_a_ values in MeCN and the gathered p*K*_a_ values in DMSO were used for a correlation analysis between the two solvents and for assessing the quality of p*K*_a_ conversion equations. A number of p*K*_a_ values have been predicted in MeCN from p*K*_a_ values in DMSO via the correlation analysis and p*K*_a_ conversion equations.

## Introduction

In most chemical processes, the acidities or basicities of the involved compounds are essential, particularly when charged species are involved. The most widely used representation of the acidity of a molecule in the liquid phase is based on the Brønsted theory^1^ and is quantitatively expressed by the equilibrium constant *K*_a_ of the acid dissociation reaction in solvent S (eq 1) or, more often, its negative logarithm p*K*_a_ (eq 2).
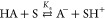
1
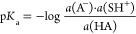
2When dealing with chemical reactions in the presence of strong bases, in order to control the reaction path and outcomes it is important to understand which of the solution components can get deprotonated by the chosen base. For that, knowing the p*K*_a_ values of even very weak acids is crucial. As the majority of such reactions are carried out in nonaqueous media, it is the p*K*_a_ values in organic solvents such as acetonitrile (MeCN) and dimethyl sulfoxide (DMSO) that are relevant.

One significant type of acids are NH acids, in which the acidic proton is directly attached to the nitrogen atom. NH acids cover a wide range of p*K*_a_ values, from superacidic catalysts^2^ to the weakly acidic indole and its derivatives.^3−5^ In MeCN, the p*K*_a_ values of numerous superacidic NH have been reported,^6^ and the Bordwell group has published the p*K*_a_ values of numerous weak NH acids in DMSO.^3,7^ However, to the best of our knowledge, only very limited information is available regarding the acidities of very weak acids (p*K*_a_ > 30) in MeCN.

Prominent classes of weak acids lacking experimental p*K*_a_ data are conjugated nitrogen heterocycles and aromatic amines - compounds that are usually better known as bases. Compounds such as benzimidazole, benzotriazole, indazole, carbazole, azaindoles and especially their derivatives have numerous applications: these moieties are found in pharmaceuticals,^8^ pesticides, bioactive compounds,^9^ etc. Nitrogen heterocycles are one of the most significant structural fragments in pharmaceuticals^10^ as their derivatives exhibit significant anti-inflammatory, antiviral, antihistaminic, antiparasitic, antifungal and anticancer activity.^11−13^ Despite the available acidities in DMSO, their p*K*_a_ values in a less basic solvent like MeCN are also of interest. Yet, no such data have been published. Some acids of interest also have no p*K*_a_ values in DMSO. For instance, to the best of our knowledge, the acidities of azaindoles and benzocarbazoles have not been measured in any nonaqueous solvents before.

Besides the above-mentioned fields of application, weak NH acids are well-suited additions to the previously published comprehensive self-consistent acidity scale in acetonitrile, which contains p*K*_a_ values of 231 acids and covers close to 30 orders of magnitude of acidity.^6^ Despite the substantial number of acids in it, the p*K*_a_ scale in MeCN is scarce in compounds in the region of the weakest acids. It contains only five acids with p*K*_a_ values between 29 and 32.57 (the highest p*K*_a_ value on the scale). For comparison, between p*K*_a_ values of 25.4 and 29, there are 34 acids on the scale. The available acidity data in MeCN show that the p*K*_a_ values of aromatic amines, such as substituted diphenylamines and anilines, can vary over a wide range depending on the number and properties of the substituents.^6^ Thus, aromatic amines with a suitable structure and substituent groups should have p*K*_a_ values in the desired region.

MeCN as a solvent is generally not considered as suitable as DMSO for studying very weak acids because of its lower basicity.^14^ Nevertheless, numerous acids are expected to have p*K*_a_ values above 29 in MeCN, enabling supplementing and expanding the current acidity scale in MeCN. As the highest p*K*_aH_ (p*K*_a_ value of the conjugated acid of a base)^14^ value of the MeCN basicity scale is 33.14,^15^ extending the acidity scale to at least a similarly high p*K*_a_ value should be possible.

The aim of this study was to present a number of new previously unavailable p*K*_a_ values of weak NH acids in MeCN and to expand the p*K*_a_ scale in MeCN. In addition, this study aims to evaluate the previously published simple conversion equations for p*K*_a_ estimations between DMSO and MeCN,^6^ specifically for weak NH acids, and to predict a number of additional p*K*_a_ values in MeCN. Also, computational gas-phase acidity (GA) values were calculated for all the compounds studied in this work.

## Results and Discussion

### Expanded p*K*_a_ Scale in Acetonitrile

The p*K*_a_ values of 37 weak NH acids, mainly conjugated nitrogen heterocycles and aromatic amines, were determined in MeCN by 141 relative acidity measurements. As a result, the least acidic section of the MeCN p*K*_a_ scale,^6^ with p*K*_a_ values over 29, was rebuilt and expanded by adding 31 new compounds. Consequently, the highest p*K*_a_ value on the scale is now 33.51 belonging to 2,3,5,6-Cl_4_-aniline. The previously published p*K*_a_ values for indole (32.57 → 32.78), 3-MeCOO-indole (29.97 → 30.2) and 7-NO_2_-indole (29.99 → 30.12) were revised. The results are presented in Table 1. The p*K*_a_ values of the aromatic amines presented in this work include some of the highest ever experimentally determined p*K*_a_ values of neutral NH acids in MeCN. In addition to the results in Table 1, the p*K*_a_ value of benzotriazole in MeCN was determined to be 22.98 (see Table S1 in the SI).

**Table 1:**
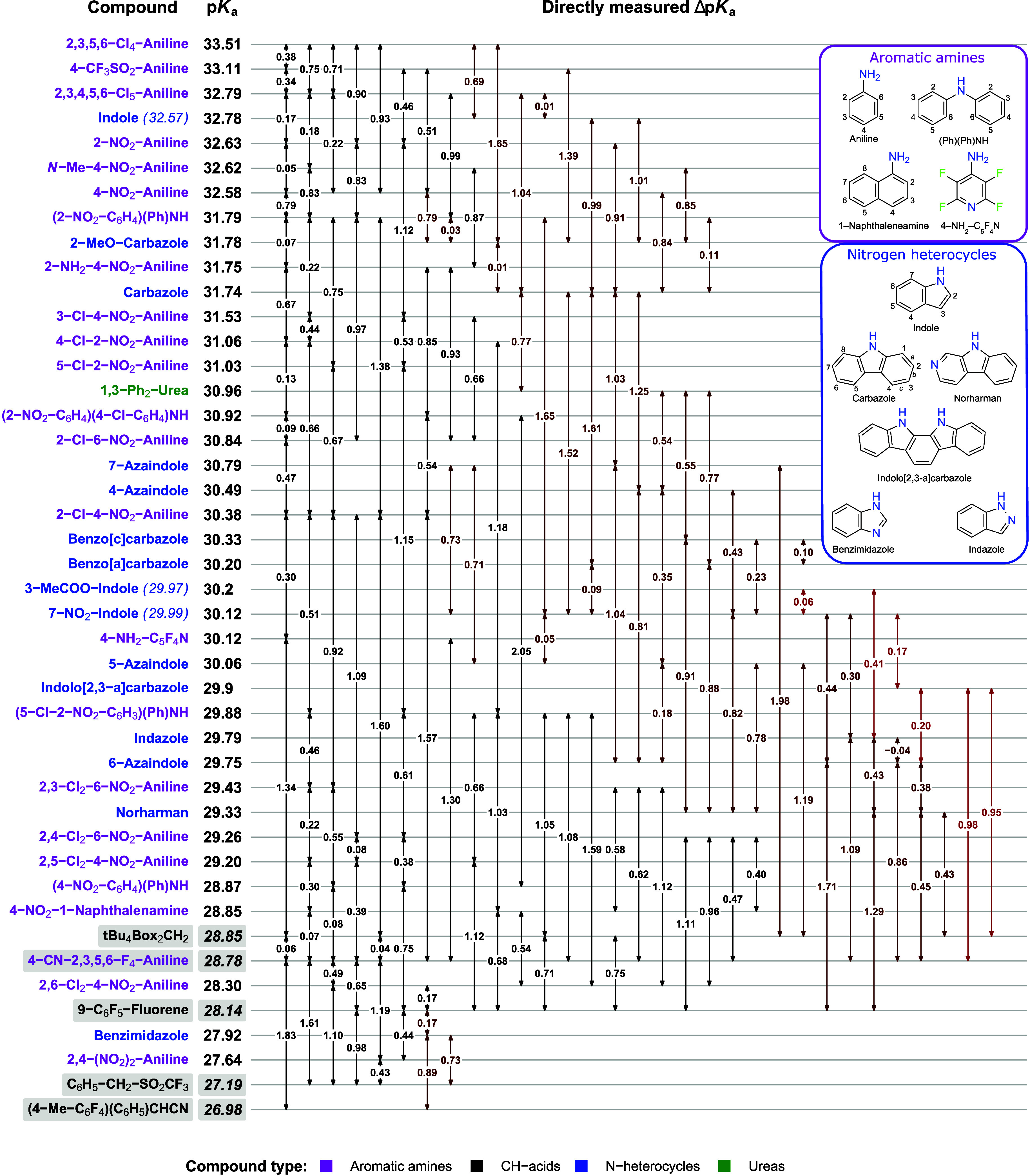
Acidity Measurements and Assigned p*K*_a_ Values of Weak NH Acids in MeCN^a^

a Compounds with a gray background serve as anchor compounds with fixed p*K*_a_ values from ref (6) Previously published p*K*_a_ values (from ref (6)) are given for indole, 3-MeCOO-indole and 7-NO_2_-indole in parentheses for comparison. Black, brown and red arrows denote high-, medium- and low-reliabiliy Δp*K*_a_ measurements, respectively, depending on the type of acids. See text for more details.

The p*K*_a_ scale in Table 1 was built by performing relative acidity (relative p*K*_a_ or Δp*K*_a_) measurements. Each double-headed arrow corresponds to a Δp*K*_a_ determination between a pair of acids (described in more detail in the Experimental Section and SI). Absolute p*K*_a_ values of the analyzed acids were assigned using the “ladder” approach,^6,16^ i.e. the following least-squares minimization procedure with the experimentally determined relative acidities.
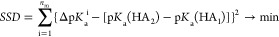
3According to eq 3, the sum of squares of the differences between all the experimentally determined relative acidities Δp*K*_a_ and the differences between the assigned absolute p*K*_a_ values of acids HA_2_ and HA_1_ over all the relative acidity determination series *n*_m_ was minimized. If absolute p*K*_a_ values are desired as a result of the minimization then the ladder has to contain at least one reference acid (anchor acid) with a previously known and reliable p*K*_a_ value. This p*K*_a_ value is kept constant while the p*K*_a_ values of the remaining compounds are varied until the minimal *SSD* is reached. The acids *t*Bu_4_Box_2_CH_2_,^17^ 4–CN–2,3,5,6–F_4_–aniline, 9–C_6_F_5_–fluorene, C_6_H_5_–CH_2_–SO_2_CF_3_ and (4–Me–C_6_F_4_)(C_6_H_5_)CHCN have known reliable p*K*_a_ values^6^ and were used as reference acids in the least-squares minimization process.

Before the minimization, the analyzed acids were divided into two groups – “backbone” and “secondary” acids–according to the reliability of the relative acidity measurements conducted with them. The acids with the most convenient properties that enable high-quality Δp*K*_a_ measurements were used as the backbone acids. As described in a previous publication, the reliability of the measurements was evaluated by considering the spectral properties of the acids, consistency of values obtained within a measurement series, self-consistency of the Δp*K*_a_ values obtained for a given compound against different reference compounds and the possibility of unwanted side processes.^6^ Aromatic amines were chosen as the “backbone” acids because of their very suitable spectral properties, good within-series agreement, and good consistency of measurements. As a rule, nitrogen heterocycles displayed somewhat worse performance in p*K*_a_ measurements and were categorized as “secondary” acids. The third category consists of indolo[2,3-*a*]carbazole and 3-MeCOO-indole. Even after additional purification, the relative acidity measurements of these compounds were found to be less reliable than those of the other studied compounds. This could be caused by possible side reactions or the presence of impurities, which influenced the UV–vis spectra of these compounds during the spectrophotometric titration in such a way that the self-consistency and accuracy of the experimental results were lowered.

In total, three minimization steps were performed to assign p*K*_a_ values for all analyzed compounds. All Δp*K*_a_ measurements involving “backbone” acids are presented as black-colored double-sided arrows in Table 1. The arrows of “secondary” acids are brown, and those involving indolo[2,3-*a*]carbazole or 3-MeCOO-indole are red.

For the first minimization step, only the relative p*K*_a_ values between aromatic amines and relative p*K*_a_ values between aromatic amines and the above-mentioned reference acids were used. As a result of this minimization, p*K*_a_ values for the “backbone” acids were assigned. In the second minimization step the acidities of the “backbone” acids were kept constant and used as reference to obtain the p*K*_a_ values for the “secondary” acids. The third minimization was performed to obtain the p*K*_a_ values of 3-MeCOO-indole and indolo[2,3-*a*]carbazole using all other compounds, which had p*K*_a_ values assigned in the previous steps, as reference acids.

The quality of a p*K*_a_ scale formed by the relative p*K*_a_ determination approach can be evaluated using the consistency standard deviation *s* of the scale, according to the following equation:
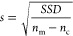
4In eq 4, *SSD* is the sum of squares found in eq 3, *n*_m_ is the number of measurement series, and *n*_c_ is the number of compounds for which an absolute p*K*_a_ value was assigned. Strictly speaking, the parameter *s* is not interpretable as the uncertainty of an individual p*K*_a_ value in the scale. However, it serves as a general estimate of the quality of the p*K*_a_ values forming the acidity scale, can be loosely interpreted as the average standard uncertainty of the p*K*_a_ values with respect to the scale, and can be used for comparing the strengths of the acids belonging to the scale. The consistency standard deviation of the Δp*K*_a_ measurements involving aromatic amines (“backbone” acids) is 0.03. It is the same consistency as that of the “backbone” acids forming the overall p*K*_a_ scale in MeCN spanning almost 30 orders of magnitude and can be rated as good.^6^ The *s* value of the Δp*K*_a_ measurements involving the “secondary” acids is 0.06 and the *s* value of the Δp*K*_a_ measurements involving indolo[2,3-*a*]carbazole or 3-MeCOO-indole is 0.09, which can both be considered satisfactory.

The acidity scale published in 2021 by Kütt et al.^6^ was scarce in compounds with p*K*_a_ values over 30. The compounds indole, 3-MeCOO-indole and 7-NO_2_-indole were already present in that scale. However, because of the lack of other compounds with similar p*K*_a_ values, measurements of considerable p*K*_a_ differences (Δp*K*_a_ values around or above 2) were used to assign p*K*_a_ values for these compounds. Usually, experimental Δp*K*_a_ values higher than 1.5 are less accurate than lower Δp*K*_a_ values because of the narrow (or nonexistent) range within which both measured acids have accurately measurable degrees of dissociation (i.e., degrees of dissociation between 0.1 and 0.9). Thus, such results need confirmatory measurements. The present work adds a large number of acids to the p*K*_a_ range of 30 to 33, completely eliminating the need for measuring large Δp*K*_a_ values. The results from the present work indicate that such higher than recommended Δp*K*_a_ measurements caused a contraction of the upper part of the acidity scale. The following four earlier experimental relative acidity measurements^6^ seem to be the main cause of this effect (directly measured Δp*K*_a_ values in parentheses): 3-MeCOO-indole vs 5-NO_2_-indole (Δp*K*_a_ = 1.82), 7-NO_2_-indole vs 9-C_6_F_5_-fluorene (Δp*K*_a_ = 1.88), 7-NO_2_-indole vs 6-NO_2_-indole (Δp*K*_a_ = 2.21) and 7-NO_2_-indole vs 5-NO_2_-indole (Δp*K*_a_ = 1.74). The corresponding Δp*K*_a_ values calculated from the results of this work (Table 1) are systematically higher by 0.1–0.2 p*K*_a_ units. Thus, these four Δp*K*_a_ measurements were considered unreliable and were excluded from the data analysis. The previous^6^ directly measured Δp*K*_a_ values for the acid pairs indole vs 3-MeCOO-indole (Δp*K*_a_ = 2.58) and indole vs 7-NO_2_-indole (Δp*K*_a_ = 2.61) are in agreement with the absolute p*K*_a_ values in the scale presented in Table 1. Nevertheless, these two previous Δp*K*_a_ measurements were also considered doubtful (in part because of the very high Δp*K*_a_ value–far above the reliable directly measured Δp*K*_a_ range of the used method of up to approximately 1.5 p*K*_a_ units) and excluded from the data analysis.

### Comparison of Acidities between Different Solvents and the Gas Phase

The experimental p*K*_a_ values in DMSO and H_2_O, as well as the gas-phase acidity (GA) values (found in literature and/or calculated) are presented in Table 2 together with the p*K*_a_ values determined in this work. The p*K*_a_ values in DMSO and H_2_O have been published for less than half of the compounds of the present work. In the case of water, the obvious reason for the scarcity is that the aqueous p*K*_a_ values of the majority of the investigated compounds are outside the common aqueous p*K*_a_ range. In the case of DMSO, all the values are well within the experimentally achievable p*K*_a_ range in this solvent. Data from the Bordwell^3,7^ group were preferred if p*K*_a_ values from multiple sources were available. The available experimental gas-phase acidity (GA) data was even more scarce than in either of the two solvents. The GA values of only seven studied compounds were found in the NIST Chemistry WebBook,^18^ so computational GA values are also provided in Table 2. The root-mean-square difference between the available experimental GA values and their computational counterparts is 10 kJ mol^–1^ and the maximum difference is 15 kJ mol^–1^ (for comparison, a typical uncertainty of GA values reported in the NIST database is ±8.4 kJ mol^–1^). Most of the computational values are higher than the experimental values, displaying an average bias of 8 kJ mol^–1^. The possible reason for these comparatively high discrepancies could be a compound class-specific bias that some methods exhibit, method-specific systematic bias (which, in turn, could originate from overestimation of vibrational frequencies),^19^ or the interplay between the two.

**Table 2:** Acidities in Different Solvents and the Gas Phase

Compound	CAS RN	p*K*_a_ (MeCN)^a^	p*K*_a_ (DMSO)	p*K*_a_ (H_2_O)	Exp. GA,^b^ kJ mol^-1^	Calc. GA,^c^ kJ mol^-1^
2,3,5,6-Cl_4_-Aniline	3481–20–7	33.51	21.0^20^	19.22^21^		1426
4-CF_3_SO_2_-Aniline	473–27–8	33.11	21.8^7^			1400
2,3,4,5,6-Cl_5_-Aniline	527–20–8	32.79				1415
Indole	120–72–9	32.78	20.95^3^	16.97^22^	1431;^23^ 1440^24^	1441
2-NO_2_-Aniline	88–74–4	32.63		17.7^25^		1436
N-Me-4-NO_2_-Aniline	100–15–2	32.62		18.2^25^		1421
4-NO_2_-Aniline	100–01–6	32.58	20.9^7^	18.2^25^	1407^26^	1421
(2-NO_2_–C_6_H_4_)(Ph)NH^g^	119–75–5	31.79	19.2^d^ (17.7^27^)	18.0^25^		1421
2-MeO-Carbazole	6933–49–9	31.78				1417
Carbazole	86–74–8	31.74	19.9^3^	16.7^28^	1412^23^	1420
2-NH_2_-4-NO_2_-Aniline	99–56–9	31.75				1416
3-Cl-4-NO_2_-Aniline	825–41–2	31.53				1403
4-Cl-2-NO_2_-Aniline	89–63–4	31.06	18.9^3^	17.1^21^		1412
5-Cl-2-NO_2_-Aniline	1635–61–6	31.03				1411
1,3-Ph_2_-Urea	102–07–8	30.96	19.6^3^			1396
(2-NO_2_–C_6_H_4_)(4–Cl-C_6_H_4_)NH	23008–56–2	30.92				1402
2-Cl-6-NO_2_-Aniline	769–11–9	30.84				1423
7-Azaindole	271–63–6	30.79		12.1^29^^,^^f^		1437
4-Azaindole^e^	272–49–1	30.49		15.5^30^^,^^e^; 16.1^,^^30^^,^^e^		1422
2-Cl-4-NO_2_-Aniline	121–87–9	30.38		18.06^31^		1398
Benzo[c]carbazole	205–25–4	30.33				1401
3-MeCOO-Indole	608–08–2	30.2				1427
Benzo[a]carbazole	239–01–0	30.20				1400
7-NO_2_–Indole	6960–42–5	30.12				1415
4-NH_2_–C_5_F_4_N	1682–20–8	30.12	18.7^d^ (19.2^32^)		1392^33^	1407
5-Azaindole	271–34–1	30.06				1414
Indolo[2,3-*a*]carbazole	60511–85–5	29.9				1387
(5-Cl-2-NO_2_–C_6_H_3_)(Ph)NH	25781–92–4	29.88				1397
Indazole	271–44–3	29.79	18.2^28^	13.86^28^	1425^34^	1433
6-Azaindole	271–29–4	29.75				1413
2,3-Cl_2_-6-NO_2_-Aniline	65078–77–5	29.43				1403
Norharman	244–63–3	29.33		14.53^35^		1398
2,4-Cl_2_-6-NO_2_-Aniline	2683–43–4	29.26		16.39^31^		1401
2,5-Cl_2_-4-NO_2_-Aniline	6627–34–5	29.20	17.4^7^	16.05^36^		1380
(4-NO_2_–C_6_H_4_)(Ph)NH^g^	836–30–6	28.87	16.85^7^	15.6^25^	1374^33^^,^^g^	1384
4-NO_2_-1-Naphthalenamine	776–34–1	28.85	17.4^d^ (18.0^37^)			1385
2,6-Cl_2_-4-NO_2_-aniline	99–30–9	28.30		15.55^36^		1387
Benzimidazole	51–17–2	27.92	16.4^3^	12.86^38^		1403
2,4-(NO_2_)_2_-Aniline	97–02–9	27.64	15.9^7^	15.0^36^		1367
1H-Benzotriazole	95–14–7	22.98	11.92^3^	8.57^39^	1384^34^	1383

a p*K*_a_ values from this work (Table 1).

b Experimental GA values.

c Computational GA values (this work).

d Original values from the respective publications (shown in parentheses) were corrected in ref (20)

e In the abstract and the main text of ref (30) two different p*K*_a_(H_2_O) values were presented for 4-azaindole and it was not possible to tell which one is the correct one.

f Doubtful value.

g The GA value of 1374 kJ mol^–1^, actually belonging to (4-NO_2_–C_6_H_4_)(Ph)NH, is erroneously assigned to (2-NO_2_–C_6_H_4_)(Ph)NH in the NIST Webbook.

**Figure 1:**
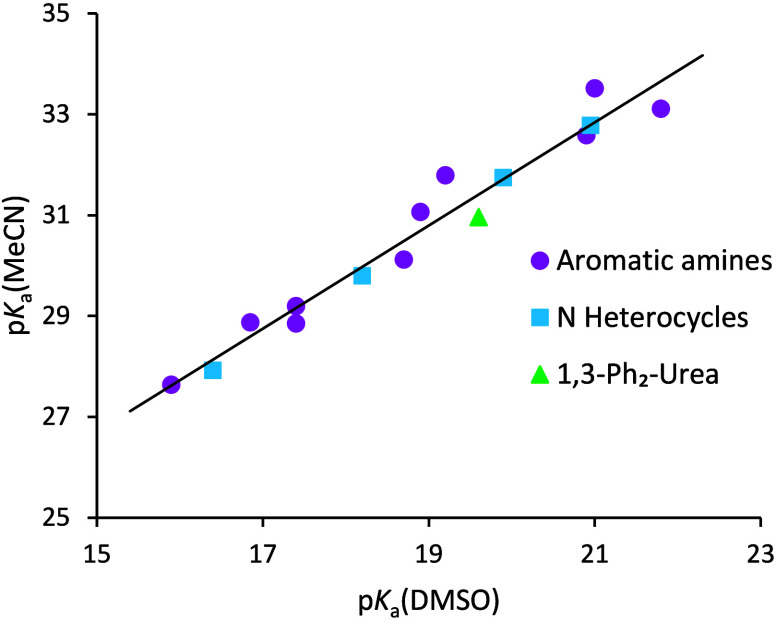
Correlation between p*K*_a_ values in MeCN and DMSO.

Fifteen compounds studied in this work had p*K*_a_(DMSO) values available in the literature (ten aromatic amines, four nitrogen heterocycles and 1,3-diphenylurea). Using these values and the respective p*K*_a_(MeCN) values determined in the present work, a correlation between acidities in the two solvents was composed (Figure 1). Benzotriazole was excluded from the correlation because of its almost 5 orders of magnitude higher acidity compared to the other compounds. The following correlation equation was obtained:
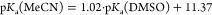
5
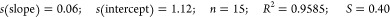


Figure 1 shows that the acidities of the selection of NH acids correlate reasonably well between DMSO and MeCN without any strongly deviating compounds. Solvent–solvent correlations are expected to be good if the solvents have sufficiently similar properties and the involved acids are of a similar nature.^14^ Both requirements are fulfilled in this case. Better correlations could be obtained for heterocycles and aromatic amines separately. The adequacy of the regression equation is evidenced by the fact that if the p*K*_a_ of benzotriazole in MeCN is predicted from it, the value will be 23.6, which is approximately 0.5 p*K*_a_ units different from the experiment. Considering the significant extrapolation involved, the agreement can be considered good.

### Equations for p*K*_a_ Conversions between Solvents

Kütt et al.^6^ developed several equations to predict the p*K*_a_ values of acids in different nonaqueous solvents based on p*K*_a_ values in MeCN and simple structure-based descriptors. Equations 6 (eq 2.3 in Table 2 in ref (6)) and 7 (eq 2.1 in Table 2 in ref (6)) are the universal and NH acid-specific equations, respectively, to convert p*K*_a_(MeCN) values to p*K*_a_(DMSO) values. These equations have a root-mean-square error (RMSE) of prediction of 0.5 and 1.2, respectively.
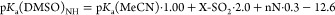
6
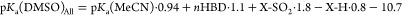
7

The p*K*_a_ values measured in this work offer a convenient possibility to assess the quality of these equations, as most compounds in this work (a) were not used in the training data set for developing the equations and (b) have higher p*K*_a_ values than the majority of the training set compounds. Thus, they serve as a good and demanding test set. The accuracy of p*K*_a_(DMSO) values from the literature was critically evaluated, and the corrected p*K*_a_(DMSO) values of (2-NO_2_–Ph)(Ph)NH, 4-NH_2_–C_5_F_4_N, 4-NO_2_-1-Naphthalenamine were used (see Table 2).

The descriptors used in eqs 6 and 7 are the number of hydrogen bond donors (*n*HBD), the number of hydrogen atoms or sulfonyl groups attached directly to the acidity center (X-H, X-SO_2_), and the number of nitrogen atoms in the molecule (*n*N). The equations were rearranged in such a way as to predict the p*K*_a_ values in MeCN from values in DMSO and the predicted values were compared to the experimental p*K*_a_ values determined in this paper. The results are presented in Table 3.

**Table 3:** p*K*_a_(MeCN) Values Predicted Using Conversion Equations (Eqs 6 and 7)^a^

		Prediction from p*K*_a_(DMSO)			
Compound	p*K*_**a**_(MeCN)^d^	NH^b^	Difference	All^c^	Difference
2,3,5,6-Cl_4_-Aniline	33.51	**3**3.30	–0.21	33.09	–0.42
4-CF_3_SO_2_-Aniline	33.11	34.10	0.99	33.94	0.83
4-NO_2_-Aniline	32.58	32.90	0.32	32.98	0.40
(2-NO_2_–C_6_H_4_)(Ph)NH	31.79	31.20	–0.59	31.49	–0.30
Carbazole	31.74	32.20	0.46	32.23	0.49
4-Cl-2-NO_2_-Aniline	31.06	30.90	–0.16	30.85	–0.21
1,3-Ph_2_-Urea	30.96	31.60	0.64	30.74	–0.22
4-NH_2_–C_5_F_4_N	30.12	30.70	0.58	30.64	0.52
Indazole	29.79	30.20	0.41	30.43	0.64
2,5-Cl_2_-4-NO_2_-Aniline	29.20	29.40	0.20	29.26	0.06
(4-NO_2_–C_6_H_4_)(Ph)NH	28.87	28.85	–0.02	28.99	0.12
4-NO_2_-1-Naphthalenamine	28.85	29.40	0.55	29.26	0.41
Benzimidazole	27.92	28.40	0.48	28.51	0.59
2,4-(NO_2_)_2_-Aniline	27.64	27.60	–0.04	27.66	0.02
Benzotriazole	22.98	23.62	0.64	23.74	0.76

		RMSE^e^	0.49		0.46

a Conversion equations taken from ref (6).

b p*K*_a_(MeCN) values predicted from p*K*_a_(DMSO) using eq 6.

c p*K*_a_(MeCN) values predicted from p*K*_a_(DMSO) using eq 7

d Experimental values from this work.

e Root mean square error.

The RMSE of the p*K*_a_(MeCN) prediction of the selection of weak acids is 0.49 when using the NH acid-specific and 0.46 when using the universal equation suitable for all types of acids. These RMSE values are equal to or lower than the RMSE values of p*K*_a_(MeCN) to p*K*_a_(DMSO) conversion equations in the original publication (0.5 and 1.2, respectively). This result is made even more noteworthy by considering that these equations were created using different compound classes (mainly sulfonamides and diarylamines), which are typically stronger acids than the aromatic amines and nitrogen heterocycles studied in this work.

Although similar p*K*_a_ conversion equations were published in ref (6) for the water-MeCN pair, these almost always yield less accurate results than conversions between DMSO and MeCN. Aqueous p*K*_a_ values from Table 2 were used to predict the p*K*_a_ values of the respective acids in MeCN, but the obtained RMSE was significantly over 2, which shows that the conversion equations between water and MeCN from ref (6) are unusable for such weak acids. One reason for this large discrepancy is the much different nature of both solvents. MeCN is an aprotic solvent with very weak hydrogen bond donor (HBD) and hydrogen bond acceptor (HBA) properties, whereas water is a protic solvent with strong HBD and HBA properties, thus having the ability to specifically solvate anions. Usually, better correlations of acidities are obtained between solvents with similar properties.^14^ Another reason is that the majority of the aqueous p*K*_a_ values of the compounds studied in this paper are outside the conventional aqueous pH scale (0–14). This means that certain approximations (regarding transferability of p*K*_a_ values from highly concentrated aqueous alkali solutions or aqueous organic mixtures into dilute aqueous solutions)^25^ have been involved in obtaining these values, which invariably make the values less reliable.

Due to the low RMSE of prediction on the basis of p*K*_a_(DMSO) values, the acidities of a selection of well-known heterocycles were estimated in MeCN using their p*K*_a_ values in DMSO. The results are presented in Table 4. To our knowledge, no experimental p*K*_a_ values have yet been determined in MeCN for pyrrole, pyrazole, imidazole, 1,2,3-triazole, and 1,2,4-triazole.

**Table 4:** Estimated p*K*_a_ Values of Some Heterocyclic Compounds

			p*K*_a_(MeCN) estimated from p*K*_a_(DMSO)					
Compound	CAS RN	p*K*_a_ (DMSO)^a^	eq 5	eq 6	eq 7	Recommended p*K*_a_ (MeCN)^b^	Exp. GA^c^ [kJ mol^-1^]	Calc. GA^d^ [kJ mol^-1^]
Pyrrole	109–97–7	23.0	34.9	35.3	35.5	35.2 ± 0.5	1468;^40^ 1472^41^	1482
Pyrazole	288–13–1	19.8	31.6	31.8	32.1	31.8 ± 0.5	1449^42^	1463
Imidazole	288–32–4	18.6	30.4	30.6	30.9	30.6 ± 0.5	1434^42^	1443
1,2,4-Triazole	288–88–0	14.75	26.5	26.5	26.8	26.6 ± 0.5	1410^18^	1419
1*H*-1,2,3-Triazole	288–36–8	13.9	25.6	25.6	25.9	25.7 ± 0.5	1419^34^	1412

a All experimental values from the Bordwell group.^3^

b Recommended p*K*_a_ values based on the arithmetic mean of estimations from DMSO, uncertainty estimates refer to standard uncertainty.

c Experimental GA values.

d Computational GA values from this work.

### Structural Effects on Acidity

**Scheme 1:**
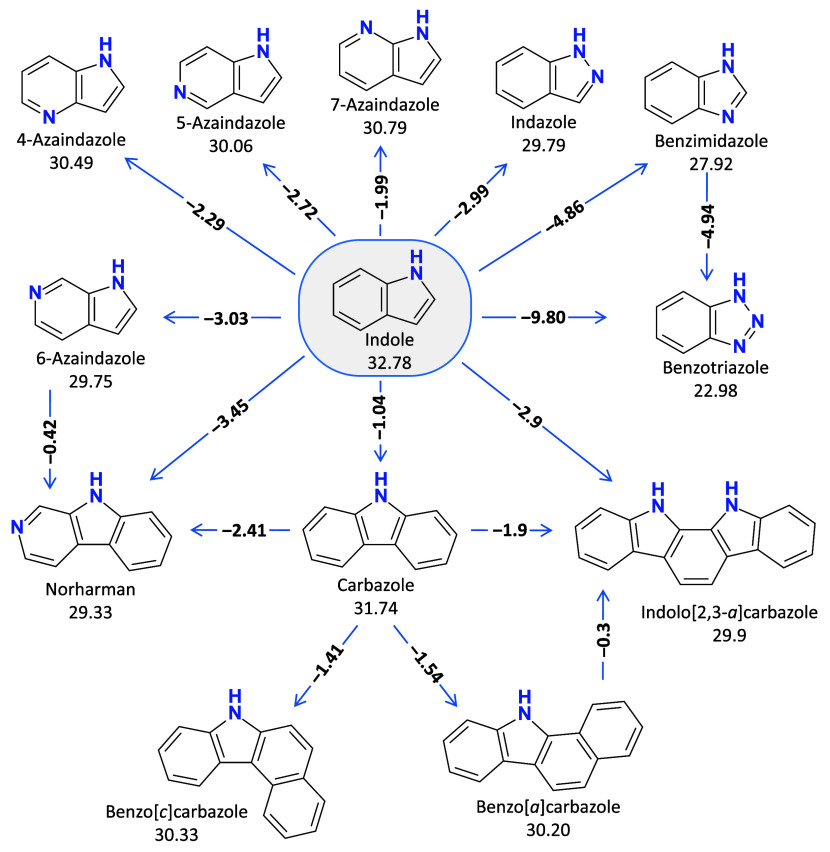
Relations between Structural Features and Acidities of Indole Derivatives Absolute p*K*_a_ values (determined in this work) are given below the compound names. p*K*_a_ differences between compounds are shown on the arrows.

Scheme 1 shows the effects of structural differences on the p*K*_a_(MeCN) values of nitrogen (N) heterocycles related to indole. The compounds indazole, benzimidazole and benzotriazole can be viewed as indole derivatives with two or three ring nitrogen atoms. Adding N atoms into the 5-membered indole ring has an acidity-enhancing effect, the extent of the effect depending on their position. Indazole (N in position 2) has by 2.99 units lower p*K*_a_ value than indole, whereas the p*K*_a_ of benzimidazole (N in position 3) is by 4.86 units lower. Similar acidity differences were observed in DMSO, 2.75 and 4.55 p*K*_a_ units, respectively.^3^ The increase in acidity is caused by a combination of the inductive and resonance effects, which stabilize the anion. Although in benzimidazole, the second N is further away from the acidity center, thus having a lower inductive effect, it has a more substantial resonance effect, resulting in a lower p*K*_a_ value than in the case of indazole. Benzotriazole has by 9.80 units lower p*K*_a_ than indole. The third N in the five-member ring further stabilizes the anion. Interestingly, the Δp*K*_a_ of indole and benzimidazole with 4.86 is almost the same as the Δp*K*_a_ of benzimidazole and benzotriazole with 4.94. In DMSO, the acidity enhancing effect of the two additional N atoms is even larger, amounting to 11.92 units.^3^

The acidifying effect of an N atom in the six-membered ring of azaindoles is weaker due to the longer distance from the acidity center. The variation in p*K*_a_ values of the azaindoles with an N atom in different positions is small, spanning around 1 p*K*_a_ unit. 7-Azaindole is by 1.99, 4-azaindole by 2.29, 5-azaindole by 2.72 and 6-azaindole by 3.03 p*K*_a_ units stronger acid than indole. In the case of norharman, which has by 2.41 p*K*_a_ units lower p*K*_a_ value than carbazole, the extent of this acidifying effect is comparable to that of azaindoles.

Comparing the p*K*_a_ values of indole and carbazole reveals that the acidity-enhancing effect of a fused aromatic ring is smaller than the effect of additional N atoms, and carbazole is only by 1.04 p*K*_a_ units stronger acid than indole. The effect is almost identical in DMSO with 1.05 units.^3^ The effect of a fused aromatic ring in 6-azaindole is still lower, and accordingly, the p*K*_a_ value of norharman is only 0.42 units lower than the p*K*_a_ of 6-azaindole. Somewhat surprisingly, the effect of adding a second fused benzene ring into the carbazole molecule has a slightly higher impact on the p*K*_a_ value. Benzo[*c*]carbazole and benzo[*a*]carbazole are more acidic than carbazole by 1.41 and 1.54 units, respectively. Although the benzene ring fused to the c side of carbazole is further away from the acidity center than the one fused to the a side, the difference between the p*K*_a_ values of these two compounds is marginal. Indolo[2,3-*a*]carbazole can be viewed as a carbazole with an indole fused to its a side. Indolo[2,3-a]carbazole has by 0.3 units lower p*K*_a_ value than benzo[a]carbazole. The NH fragment close to the deprotonated acidity center might have some anion-stabilizing effect.

2-NO_2_-Aniline and 4-NO_2_-aniline have similar p*K*_a_ values in MeCN - 32.63 and 32.58, respectively. Replacing one of the hydrogens in their amino groups with a phenyl group has a drastically different impact on their p*K*_a_ values. (2-NO_2_–Ph)(Ph)NH is by 0.84 p*K*_a_ units stronger acid than 2-NO_2_-aniline, but (4-NO_2_–Ph)(Ph)NH is by 3.71 units (4.05 units in DMSO) stronger acid than 4-NO_2_-aniline. This difference is likely caused by the stabilizing intramolecular hydrogen bond (IMHB) in the neutral (2-NO_2_–Ph)(Ph)NH molecule, thus making it less acidic than (4-NO_2_–Ph)(Ph)NH where no IMHB is possible. The p*K*_a_ of 4-NO_2_-1-naphthalenamine is almost identical to the p*K*_a_ of (4-NO_2_–Ph)(Ph)NH.

In MeCN, the p*K*_a_ of 4-NO_2_-aniline is by 0.53 p*K*_a_ units lower than the p*K*_a_ of 4-CF_3_SO_2_-aniline. The same trend can be observed in DMSO, where 4-NO_2_-aniline is a stronger acid than 4-CF_3_SO_2_-aniline by 0.9 p*K*_a_ units. To compare the electron-withdrawing nature of both substituents, the resonance and field inductive effect substituent constants can be used. These demonstrate that the SO_2_CF_3_ substituent (σ_F_ = 0.83, σ_R_ = 0.26) is a stronger resonance and induction acceptor than the NO_2_ substituent (σ_F_ = 0.64, σ_R_ = 0.16),^43^ at odds with the above-described acidity order. The inversion of acidity order of NO_2_ and SO_2_CF_3_ derivatives, relative to the order of substituent constants, has been described previously for other compounds and seems to depend on the solvent. In the case of the compound pairs Ph–CH_2_–NO_2_ and Ph–CH_2_–SO_2_CF_3_, the nitro-substituted compound is a stronger acid in DMSO.^44^ Goumont et al.,^45^ on the other hand, have demonstrated that the relative strength of the acidifying effect of the NO_2_ and SO_2_CF_3_ groups can be different in water and DMSO. They have shown on the example of NO_2_–CH_2_–COOEt and CF_3_SO_2_–CH_2_–COOEt that in water, the former is a stronger acid and in DMSO the latter is a stronger acid.^45^

### The Upper Limit of the Acidity Scale in MeCN

In the present work, the MeCN acidity scale was expanded by 0.94 units to a p*K*_a_ value of 33.51 (2,3,5,6-Cl_4_-aniline). Initial experiments have shown that theoretically it would be possible to expand the MeCN acidity scale even further toward higher p*K*_a_ values but there are some experimental limits. First, the anions of the studied acids need to be stable. All the NH acids studied in this work met that requirement. However, this was not valid for two CH acids whose p*K*_a_ is expected to be in the studied region. Specifically, it was observed from their UV–vis spectra that the anions of phenylacetonitrile and 4-NO_2_-toluene were not stable under the conditions used for p*K*_a_ determinations.

Second, a base is needed that is strong enough to deprotonate the acids of interest but not too strong to decompose the solvent. Previously the phosphazene bases *t*–Bu-N=P_1_(pyrr)_3_ [p*K*_aH_(MeCN) = 28.42]^15^ and Et-N=P_2_(dma)_5_ [p*K*_aH_(MeCN) = 32.94]^46^ have been used. Neither of them is sufficiently basic to fully deprotonate acids with p*K*_a_ values higher than approximately 32.5. Also Et-N=P_2_(pyrr)_5_ has been used as a deprotonating compound^46^ in MeCN, but due to its commercial unavailability and its only marginally higher p*K*_a_ value than Et-N=P_2_(dma)_5_, it was not considered. *t*-Bu-N=P_4_(dma)_9_ would be a suitable deprotonating base in terms of basicity and spectral properties but P_3_ and higher phosphazenes cause the self-condensation of acetonitrile and the formation of 4-NH_2_-2,6-Me_2_-pyrimidine.^47^ Thus, the phosphazene base HN=P_1_(tmg)_3_ was used in the present work. The p*K*_aH_ (p*K*_a_ of the conjugate acid) value of HN=P_1_(tmg)_3_ in MeCN has been estimated to be 37.2.^46^ This work marks the first time HN=P_1_(tmg)_3_ has been used as a basic titrant to deprotonate acids for the relative p*K*_a_ determinations in MeCN. Although HN=P_1_(tmg)_3_ is strong enough to deprotonate very weak acids and is stable as a free base in MeCN, it has a downside. Differently from the other mentioned strong bases, the protonated form of HN=P_1_(tmg)_3_ absorbs at wavelengths up to 290 nm (Figure S2 in the SI), meaning that it is a suitable titrant for acids that absorb at longer wavelengths, thus essentially limiting its usage to acids which have in their structure chromophores conjugated to the acidity center, e.g. nitro-substituted aromatic amines. This limitation would be absent in p*K*_a_ determinations with NMR, meaning that HN=P_1_(tmg)_3_ could be used in such p*K*_a_ determinations in the future. The phosphazene base *t*–Bu-N=P_1_(pyrr)_3_ has already been successfully used in p*K*_a_ determinations with NMR.^48^

## Conclusion

As a result of this work, the weak acid region of the MeCN acidity scale (from p*K*_a_ 29 upward), which previously contained only five acids, has been populated by 31 new compounds and extended to the p*K*_a_ value of 33.5. Altogether, 37 new p*K*_a_ values have been determined, which were among the highest experimental p*K*_a_ values of neutral NH acids in MeCN that have been reported until now. Several of the previously reported p*K*_a_ values have been revised. The experimental challenges encountered when measuring the p*K*_a_ values of very weak acids in MeCN were described and analyzed. The relations between the structure and acidity of a number of weak NH acids–nitrogen heterocycles and aromatic amines–have been discussed.

The newly determined p*K*_a_ values in MeCN were used in correlation with acidity data in DMSO to assess the quality of simple p*K*_a_ conversion equations from the literature between p*K*_a_ values in MeCN and DMSO. It was found that these equations yield p*K*_a_ values with an RMSE of prediction of 0.5, which is accurate enough for many applications. On the basis of the available data and correlation and conversion equations, some p*K*_a_ values have been predicted in MeCN. Together with computational gas-phase acidities of more than 40 compounds, this work presents over 90 new experimental or predicted acidity values—p*K*_a_ values in MeCN and computational acidity values in the gas phase.

## Experimental Section

### p*K*_a_ Determination Method

The p*K*_a_ values in MeCN were determined using the previously developed spectrophotometric titration methodology. This methodology is based on the measurement of relative acidities (Δp*K*_a_) of two acids (HA and HB). The studied equilibrium is the following:
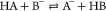
8

The logarithm of the equilibrium constant of the reaction shown in eq 8 is the difference in p*K*_a_ values of compounds HB and HA, i.e. Δp*K*_a_, as defined in eq 9.
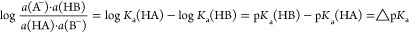
9

Methanesulfonic acid (CAS No. 75–75–2) was used as the acidic titrant during all relative acidity measurements. Phosphazene base P_2_-Et (CAS No. 165535–45–5) was used as the basic titrant for deprotonating weak acids with p*K*_a_ values under 31. Acids with p*K*_a_ values over 31 were deprotonated using the phosphazene base HN=P_1_(tmg)_3_ (CAS No. 874220–27–6). For some measurements Et-N=P_2_(pyrr)_5_ (CAS No. 874220–47–0) was used as the basic titrant. The concentrations of the acidic and basic titrant solutions were (2 × 10^–3^ – 6 × 10^–3^) mol L^–1^.

One Δp*K*_a_ measurement series consisted of three experimental steps. As the first step, a solution of acid HA in MeCN with a concentration of (1 × 10^–5^ – 2 × 10^–4^) mol L^–1^ was prepared into a 1 cm optical path length quartz cuvette and its UV–vis spectrum was registered. Then, a drop of an acidic titrant was added to the cuvette with a 100 μL Hamilton gastight syringe and a new spectrum was registered. This was done to make sure acid HA was in its neutral form in the solution. After that, a basic titrant was stepwise added until all of HA in the solution was converted to its anion A^–^. This was evidenced by the absence of changes in the absorbance spectrum of the solution after further addition of the titrant. This step yielded the UV–vis spectra of the fully protonated (HA) and deprotonated (A^–^) forms of the acid. In total, 5–10 spectra were registered. After the UV–vis spectrum of the fully deprotonated form A^–^ was recorded, an acidic titrant was stepwise added to the solution to verify the reversibility of deprotonation of HA. Although only the spectra of neutral and anionic forms were used in the calculation of the result, the spectra of the partially deprotonated HA were important for diagnostic reasons. These spectra gave information on whether the compound was pure or the presence of possible side processes (e.g., if it was decomposing during the measurement).

As the second step of the Δp*K*_a_ measurement, the same operations were done with acid HB.

As the third step, a mixture containing both HA and HB was prepared, and the same titration procedure was applied. If it was found during the individual titrations that HA and HB were already initially in their neutral forms, no acidic titrant was added, and the mixture of HA and HB was only titrated with the basic titrant. During the titration of the mixture, 15–30 spectra were recorded. It was important to have more spectra of the mixture where HA and HB are partially deprotonated than during the titration of HA and HB individually. The reason is that a Δp*K*_a_ value was calculated for every solution formed, which in turn was used to assign the final Δp*K*_a_ value for the pair of acids.

After mathematically treating the spectral data obtained from the titration of the mixture, as well as the spectra of the solutions of the individual species HA, A^–^, HB and B^–^ at multiple wavelengths using multilinear regression analysis, the dissociation levels α_HA_ = [A^–^]/([A^–^] + [HA]) and α_HB_ = [B^–^]/([B^–^] + [HB]) of both acids in all the mixtures formed during titration were obtained and were then in turn used to calculate the Δp*K*_a_ values of HA and HB according to eq 10:
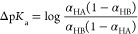
10

A more detailed description of the derivation of equations used to calculate the results of experimental Δp*K*_a_ determinations is provided in the Supporting Information (SI). The UV–vis spectra of all the acids studied in this paper can be found in the SI (Figures S4–S51).

Every weak acid studied in this paper has relative acidity measurements done against at least three other acids. The agreement between these three measurements can be visualized from Table 1 by comparing the directly measured Δp*K*_a_ values with the differences of the assigned p*K*_a_ values of the respective acids.

### Computational Method

The geometry optimization and vibrational frequency calculations were carried out at DFT BP86/def2-TZVPP level of theory with DFT-D3(BJ) dispersion correction (software: Turbomole V7.8^49^ and V7.7^50^). The absence of imaginary frequencies in the spectra was taken as proof that local energy minimum was reached. For structures with many possible conformers the most stable one was identified and its geometry was used in the further calculations.

The complete basis set (CBS) energy values were determined based on the procedure developed by Helgaker et al.^51^ For each structure, DLPNO–CCSD(T)^52^ single-point calculations were carried out using basis sets cc-pVDZ, cc-pVTZ, and cc-pVQZ (software: ORCA^53^ versions 5.0.4 and 6.0.0). For most compounds, the CBS energy value was found as an intercept of energy vs X^–3^ plot, where X = 2 for cc-pVDZ, X = 3 for cc-pVTZ and X = 4 for cc-pVQZ. For compound *t*Bu_4_Box_2_CH_2_, due to its size, the CBS energy was found from cc-pVDZ and cc-pVTZ calculations via two parameter expression with integer exponent 4.^54,55^

Gas-phase acidity (GA) values were computed as follows:
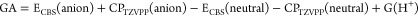
11where E_CBS_ is CBS energy computed as described above, CP_TZVPP_ is chemical potential obtained from the TZVPP calculation using the freeh module in Turbomole, and G(H^+^) is Gibbs energy of the proton (−6.275 kcal mol^–1^). Chemical potential is equal to H – TS, where H is enthalpy, T is temperature and S is entropy.

For comparison, GA values of several compounds were calculated with G4MP2^56^ method (software: Gaussian 16 Rev A.03^57^). The G4MP2 results were very well correlated with CBS results:
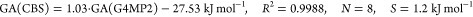
12

G4MP2 is one of the most accurate of the relatively computationally affordable methods for GA calculation.^19^ Our results suggest that for the studied compounds it does not fall short of the used combination of DLPNO–CCSD(T) and BP86/TZVPP.

The optimized geometries of the lowest-energy conformers and tabulated results of individual single-point calculations are available in the SI.

### Instruments

An Agilent Cary 60 spectrophotometer (scanning speed 600 nm/min) connected with optical fiber cables to an external cell compartment inside a commercial MBraun Unilab glovebox filled with 99.999% pure argon (5.0, Linde Gas) was used for the p*K*_a_ determinations in MeCN. This setup ensured that the moisture and oxygen contents inside the glovebox were usually under 1 ppm and always under 10 ppm during all titrations. The external cell compartment did not have a built-in thermostat but its temperature was monitored and was in the range of (24.5 ± 1.5) °C.

### Chemicals

#### Solvent

Acetonitrile (Romil 190 SpS far UV/gradient quality) was used as solvent after drying with molecular sieves (3 Å) for at least 12 h, which lowered the water content to under 6 ppm. The water content of the solvent was monitored using coulometric Karl Fischer titration. For all measurements involving aromatic amines, additionally purified MeCN was used. After drying with 3 Å molecular sieves, distillation over CaH_2_ under argon was applied. The water content after the distillation process was found to be under 10 ppm and the solvent was stored in the argon glovebox. No molecular sieves were used for further drying the solvent in order to keep it as pure as possible.

#### Studied Compounds

The following commercially obtained chemicals were used without further purification: 2,3,5,6–Cl_4_–aniline (Dr. Ehrenstorfer GmbH, 99.0%), 4–CF_3_SO_2_–aniline (Apollo Scientific, 97%), 2,3,4,5,6–Cl_5_–aniline (Fluka, 99.1%), indole (Aldrich, ≥ 99%), N–Me–4–NO_2_–aniline (Sigma-Aldrich, 97%), (2–NO_2_–C_6_H_4_)(Ph)NH (Aldrich, 98%), 2–MeO–carbazole (BLD Pharmatech, 99.94%), 2–NH_2_–4–NO_2_–aniline (Aldrich, 98%), 5–Cl–2–NO_2_–aniline (Fluka, 99%), 1,3–diphenylurea (Aldrich, 98%), (2–NO_2_–C_6_H_4_)(4–Cl–C_6_H_4_)NH (BLD Pharmatech, 97%), 2–Cl–6–NO_2_–aniline (BLD Pharmatech, 97%), 7–azaindole (Thermo Scientific, 98%), 4–azaindole (Thermo Scientific, 97%), 2–Cl–4–NO_2_–aniline (Sigma-Aldrich, 99%), benzo[*c*]carbazole (BLD Pharmatech, 97%), benzo[*a*]carbazole (BLD Pharmatech, 98%), 5–azaindole (Thermo Scientific, 98%), (5–Cl–2–NO_2_–C_6_H_3_)(Ph)NH (Alfa Aesar, 98%), indazole (Aldrich, 98%), 6–azaindole (Thermo Scientific, ≥ 97%), 2,3–Cl_2_–6–NO_2_–aniline (BLD Pharmatech, 97%), norharman (Acros Organics, 98%), 2,4–Cl_2_–6–NO_2_–aniline (BLD Pharmatech, 98%), 2,5–Cl_2_–4–NO_2_–aniline (BLD Pharmatech, 98%), (4–NO_2_–C_6_H_4_)(Ph)NH (Sigma-Aldrich, 99%), 4–NO_2_–1–Naphthalenamine (BLD Pharmatech, 98.53%), 2,6–Cl_2_–4–NO_2_–aniline (BLD Pharmatech, 98.32%), benzimidazole (Aldrich, 98%), benzotriazole (Reakhim, “pure for analysis”). The following chemicals were kind gifts and they were used as received: 3–Cl–4–NO_2_–aniline (a kind gift from Peeter Talts, University of Tartu) and 4–Cl–2–NO_2_–aniline (a kind gift from the late prof. Ilmar Koppel, University of Tartu). The following chemicals were of the same origin as in previous publications and they were used without further purification: 7–NO_2_-indole,^4^*t*Bu_4_Box_2_CH_2_,^58^ 4–CN–2,3,5,6–F_4_–aniline,^59^ 4–NH_2_–C_5_F_4_N,^59^ 9–C_6_F_5_–fluorene,^60^ C_6_H_5_–CH_2_–SO_2_CF_3_,^6^ (4–Me–C_6_F_4_)(C_6_H_5_)CHCN,^60^ 2,4–(NO_2_)_2_–aniline,^61^ 4–NO_2_–aniline,^62^ 2–NO_2_–aniline.^62^ The purity of the chemicals was assessed while observing their UV–vis spectra during titrations and the compounds were declared pure enough if no unknown absorbance changes were seen and if the isosbestic points (if present) were sharp. Carbazole (Aldrich) was recrystallized from ethanol, and 3–MeCOO-indole (Chemapol) was recrystallized twice from 40% methanol before usage. Indolo[2,3-*a*]carbazole was the same as used in a previous publication^65^ and it was additionally recrystallized from ethanol.

#### Titrants

Methanesulfonic acid (Aldrich, ≥ 99%) was used to prepare the acidic titrant solution. Phosphazene base P_2_-Et (Sigma-Aldrich, ≥ 98%), Et-N=P_2_(pyrr)_5_ (same as used in a previous publication^63^) or HN=P_1_(tmg)_3_ (prepared for this work) were used to prepare the basic titrant solution.

#### HN=P_1_(tmg)_3_

HN=P_1_(tmg)_3_·HBF_3_ (2.02 mmol; 963 mg), KHMDS (2.06 mmol; 411 mg) and hexane (12 mL) were added to an oven-dried (140 °C) ACE pressure tube inside a glovebox. The reaction mixture was stirred overnight at 70 °C outside the glovebox under argon. The mixture was then filtered in a glovebox and hexane was removed in vacuo. The raw product was recrystallized from hexane at −30 °C to give HN=P_1_(tmg)_3_ as colorless crystals (514 mg, 65%). Some of the substrate did not react. It is likely that a finer ground powder compared to what was used is preferred since the starting compound is not soluble in hexane. Or longer reaction time should be used. The obtained spectral data corresponded to that previously reported.^63^

## Data Availability

The data underlying this study are available in the published article and its Supporting Information.

## Supporting Information Available

Optimized gas-phase geometries as a set of XYZ files (ZIP)
Computational energies in the gas phase (XLSX)
Theoretical background of the relative acidity determination method, p*K*_a_ measurement results of benzotriazole, computational GA values of reference acids, UV–vis titration spectra of all studied compounds, UV–vis spectrum of HN=P_1_(tmg)_3_, acetonitrile for p*K*_a_ determinations (PDF)
