# Supplementary material for: The Acidity of Weak NH Acids: Expanding the pKa Scale in Acetonitrile
Source: ACS Org Inorg Au. 2025 Mar 13;5(2):144–55. doi: 10.1021/acsorginorgau.4c00095 (PMC11969276; doi:10.1021/acsorginorgau.4c00095)
Supplement: Supplementary file 3 — gg4c00095_si_003.pdf [file gg4c00095_si_003.pdf]

Supporting Information

The Acidity of Weak NH Acids: Expanding the pK_a Scale in Acetonitrile

Märt Lõkov [a*], Carmen Kesküla [a], Sofja Tshepelevitsh [a], Marta-Lisette Pikma [a], Jaan Saame [a], Dmitri Trubitsõn [b], Tõnis Kanger [b], and Ivo Leito [a]

[a]*Institute of Chemistry, University of Tartu, Tartu 50411, Estonia*

[b]*Department of Chemistry & Biotechnology, Tallinn University of Technology, 12618 Tallinn, Estonia*

Table of Contents

Calculation method for relative acidity S2

pK_a measurements of benzotriazole S8

Additional computational GA values S8

  Reference acids S8

  Isomers of 1,2,3-triazole and 1,2,3-benzotriazole S9

Acetonitrile for pK_a determinations S9

HN=P_1(tmg)$_3$ UV-Vis spectrum S10

Unsuccessful pK_a determinations S11

References S12

UV-Vis titration spectra S13

Calculation method for relative acidity

To derive the calculation method for relative acidity determination of acids HA and HB, first the acidities of both acids need to be expressed. According to the Brønsted-Lowry definition, the acidity of a neutral acid HA is defined by its dissociation reaction:

$$\mathrm{HA} \xrightleftharpoons{K_a^{\mathrm{HA}}} \mathrm{H^+ + A^-} \tag{1}$$

The acidity of HA is expressed as the dissociation (acidity) constant K_a^{HA} of eq (1):

$$K_a^{\mathrm{HA}} = \frac{a(\mathrm{H^+})\cdot a(\mathrm{A^-})}{a(\mathrm{HA})} \tag{2}$$

The same is valid for acid HB. Its dissociation reaction is:

$$\mathrm{HB} \xrightleftharpoons{K_a^{\mathrm{HB}}} \mathrm{H^+ + B^-} \tag{3}$$

The acidity of HB is expressed as the dissociation constant K_a^{HB} of eq 3 according to the following equation:

$$K_a^{\mathrm{HB}} = \frac{a(\mathrm{H^+})\cdot a(\mathrm{B^-})}{a(\mathrm{HB})} \tag{4}$$

In eq 2 and eq 4 $a(\mathrm{X})$ denotes the activities of the species inside the brackets. The degree of dissociation α_{HA} of acid HA is defined by eq 5.

$$\alpha_{\mathrm{HA}} = \frac{[\mathrm{A^-}]}{[\mathrm{A^-}]+[\mathrm{HA}]} \tag{5}$$

The degree of dissociation α_{HB} of acid HB is defined by eq 6.

$$\alpha_{\mathrm{HB}} = \frac{[\mathrm{B^-}]}{[\mathrm{B^-}]+[\mathrm{HB}]} \tag{6}$$

In eq 5 and eq 6 the square brackets denote the equilibrium concentrations of the corresponding species. The analytical concentrations of acids HA and HB are defined as follows:

$$C_{\mathrm{HA}} = [\mathrm{A^-}] + [\mathrm{HA}] \tag{7}$$

$$C_{\mathrm{HB}} = [\mathrm{B^-}] + [\mathrm{HB}] \tag{8}$$

Equations 5 and 6 can be combined with equations 7 and 8 to form the following equations:

$$\alpha_{\mathrm{HA}} = \frac{[\mathrm{A}^-]}{C_{\mathrm{HA}}} \tag{9}$$

$$\alpha_{\mathrm{HB}} = \frac{[\mathrm{B}^-]}{C_{\mathrm{HB}}} \tag{10}$$

During relative acidity measurements the following equilibrium between the acids HA and HB is studied:

$$\mathrm{HA} + \mathrm{B}^- \xleftrightarrow{K} \mathrm{A}^- + \mathrm{HB} \tag{11}$$

The equilibrium constant K in eq 11 is the quotient of the acidity constants of HA and HB from eq 2 and eq 4.

$$K = \frac{a(\mathrm{A}^-)\cdot a(\mathrm{HB})}{a(\mathrm{HA})\cdot a(\mathrm{B}^-)} = \frac{K_\mathrm{a}^{\mathrm{HA}}}{K_\mathrm{a}^{\mathrm{HB}}} \tag{12}$$

The logarithm of the equilibrium constant K from eq 12 is the difference of acidity constants $\mathrm{p}K_\mathrm{a}^{\mathrm{HB}}$ (eq 4) and $\mathrm{p}K_\mathrm{a}^{\mathrm{HA}}$ (eq 2), also known as the relative acidity of HB and HA or the $\Delta\mathrm{p}K_\mathrm{a}$ value.

$$\log K = \log\frac{a(\mathrm{A}^-)\cdot a(\mathrm{HB})}{a(\mathrm{HA})\cdot a(\mathrm{B}^-)} = \log\frac{K_\mathrm{a}^{\mathrm{HA}}}{K_\mathrm{a}^{\mathrm{HB}}} = \log K_\mathrm{a}^{\mathrm{HA}} - \log K_\mathrm{a}^{\mathrm{HB}} = \mathrm{p}K_\mathrm{a}^{\mathrm{HB}} - \mathrm{p}K_\mathrm{a}^{\mathrm{HA}} = \Delta\mathrm{p}K_\mathrm{a} \tag{13}$$

Instead of activities, eq 12 can be written using equilibrium concentrations and activity coefficients f.

$$K = \frac{f_{\mathrm{A}^-}[\mathrm{A}^-] f_{\mathrm{HB}}[\mathrm{HB}]}{f_{\mathrm{HB}}[\mathrm{HA}] f_{\mathrm{B}^-}[\mathrm{B}^-]} \tag{14}$$

According to the Hammett assumption,[1] the ratios of the activity coefficients of the anions and neutral forms are the same for all acids as shown in eq 15.

$$\frac{f_{\mathrm{X}^-}}{f_{\mathrm{HX}}} = const \Rightarrow \frac{f_{\mathrm{A}^-}}{f_{\mathrm{HA}}} = \frac{f_{\mathrm{B}^-}}{f_{\mathrm{HB}}} \tag{15}$$

Using this assumption, the ratio of activities in eq 14 can be replaced with ratios of concentrations. After replacing the activity coefficients according to the Hammett assumption and using equations 7, 8, 9 and 10, eq 14 can be rewritten the following way to connect the equilibrium constant K with the degrees of dissociation α_{HA} and α_{HB}:

$$K = \frac{[\mathrm{A}^-][\mathrm{HB}]}{[\mathrm{HA}][\mathrm{B}^-]} = \frac{[\mathrm{A}^-](C_{\mathrm{HB}}-[\mathrm{B}^-])}{(C_{\mathrm{HA}}-[\mathrm{A}^-])[\mathrm{B}^-]} = \frac{\frac{[\mathrm{A}^-]}{C_{\mathrm{HA}}}\cdot\frac{C_{\mathrm{HB}}-[\mathrm{B}^-]}{C_{\mathrm{HB}}}}{\frac{C_{\mathrm{HA}}-[\mathrm{A}^-]}{C_{\mathrm{HA}}}\cdot\frac{[\mathrm{B}^-]}{C_{\mathrm{HB}}}} = \frac{\alpha_{\mathrm{HA}}(1-\alpha_{\mathrm{HB}})}{(1-\alpha_{\mathrm{HA}})\alpha_{\mathrm{HB}}} \tag{16}$$

Equation 17 shows that the difference in acidity (ΔpK_a) of acids HB and HA can be calculated using only the degrees of dissociation (α_{HB} and α_{HA}).

$$\Delta pK_a = \log\frac{[A^-][HB]}{[HA][B^-]} = \log\frac{\alpha_{HA}(1-\alpha_{HB})}{(1-\alpha_{HA})\alpha_{HB}} \quad (17)$$

The degrees of dissociation (α_{HB} and α_{HA}) can be calculated using only spectral data and the Beer-Lambert law, without the need for weighing data or measuring the activity of the H^+ ion in solution (in our case the weighing data is only used to account for dilution during titration, see below).

$$A^\lambda = \log\frac{I_0^\lambda}{I^\lambda} = \varepsilon^\lambda l[X] \quad (18)$$

In eq 18 A^λ and ε^λ denote the absorbance and molar absorption coefficient of species X at wavelength λ. The square brackets denote equilibrium concentration. I_0^λ is the initial intensity of light at wavelength λ, and I^λ is its intensity after passing through the solution with an absorption path length l.

The absorbance of a solution containing the mixture of partially deprotonated HA and HB at a given wavelength λ is affected by all forms of the acids present in the solution, and according to the Beer-Lambert law, the total absorbance can be written as follows:

$$A^\lambda = \varepsilon_{A^-}^\lambda l[A^-] + \varepsilon_{HA}^\lambda l[HA] + \varepsilon_{B^-}^\lambda l[B^-] + \varepsilon_{HB}^\lambda l[HB] \quad (19)$$

Using equations 7, 8, 9 and 10, the equilibrium concentrations $[A^-]$, [HA], $[B^-]$ and [HA] in eq 19 can be replaced:

$$A^\lambda = \varepsilon_{A^-}^\lambda l\alpha_{HA}C_{HA} + \varepsilon_{HA}^\lambda l(1-\alpha_{HA})C_{HA} + \varepsilon_{B^-}^\lambda l\alpha_{HB}C_{HB} + \varepsilon_{HB}^\lambda l(1-\alpha_{HB})C_{HB} \quad (20)$$

After opening the brackets in eq 20 the following is obtained:

$$A^\lambda = \varepsilon_{A^-}^\lambda l\alpha_{HA}C_{HA} + \varepsilon_{HA}^\lambda lC_{HA} - \varepsilon_{HA}^\lambda l\alpha_{HA}C_{HA} + \varepsilon_{B^-}^\lambda l\alpha_{HB}C_{HB} + \varepsilon_{HB}^\lambda lC_{HB} - \varepsilon_{HB}^\lambda l\alpha_{HB}C_{HB} \quad (21)$$

Equation 21 is rearranged to yield eq 22.

$$A^\lambda - \varepsilon_{HA}^\lambda lC_{HA} - \varepsilon_{HB}^\lambda lC_{HB} = \alpha_{HA}C_{HA}l(\varepsilon_{A^-}^\lambda - \varepsilon_{HA}^\lambda) + \alpha_{HB}C_{HB}l(\varepsilon_{B^-}^\lambda - \varepsilon_{HB}^\lambda) \quad (22)$$

To eliminate the analytical concentrations of HA and HB and the molar absorption coefficients from the equation, spectral data obtained in the individual titrations of HA and HB need to be used. During the individual titration of HA, the spectra of its neutral form and

fully deprotonated form A⁻ are obtained and from these spectra the absorbances of both forms $A^{\lambda}_{\mathrm{A}^-}$ and $A^{\lambda}_{\mathrm{HA}}$ at a given wavelength λ can be defined.

$$A^{\lambda}_{\mathrm{A}^-} = \varepsilon^{\lambda}_{\mathrm{A}^-} l C^{0}_{\mathrm{HA}} \tag{23}$$

$$A^{\lambda}_{\mathrm{HA}} = \varepsilon^{\lambda}_{\mathrm{HA}} l C^{0}_{\mathrm{HA}} \tag{24}$$

The same can be done for HB and B⁻.

$$A^{\lambda}_{\mathrm{B}^-} = \varepsilon^{\lambda}_{\mathrm{B}^-} l C^{0}_{\mathrm{HB}} \tag{25}$$

$$A^{\lambda}_{\mathrm{HB}} = \varepsilon^{\lambda}_{\mathrm{HB}} l C^{0}_{\mathrm{HB}} \tag{26}$$

In equations 23, 24, 25 and 26 C^{0}_{HA} and C^{0}_{HB} are the analytical concentrations of HA and HB during the individual titrations of both acids.

Next, the relative concentrations C^{rel}_{HA} and C^{rel}_{HB} are defined.

$$C^{rel}_{\mathrm{HA}} = \frac{C_{\mathrm{HA}}}{C^{0}_{\mathrm{HA}}} \tag{27}$$

$$C^{rel}_{\mathrm{HB}} = \frac{C_{\mathrm{HB}}}{C^{0}_{\mathrm{HB}}} \tag{28}$$

As shown by equations 27 and 28, the relative concentrations are the ratios of analytical concentrations of both studied acids during the HA and HB mixture titration and their respective individual titrations.

C^{rel}_{HA} and C^{rel}_{HB} values do not depend on the wavelength chosen for their computation, as long as the respective ε^{λ} values are different from zero. A wavelength range where both HA and HB contribute more or less equally to the total absorbance is chosen for the calculation of C^{rel}_{HA} and C^{rel}_{HB}. The relative concentrations C^{rel}_{HA} and C^{rel}_{HB} can be calculated from the spectrum of the mixture of the fully deprotonated HA and HB and from the individual spectra of HA and HB in fully deprotonated forms by using the least-squares minimization of *SSD* over a wavelength range $\lambda_1 - \lambda_n$.

$$SSD = \sum_{i=1}^{n} \left[A^{\lambda_i} - \left(C^{rel}_{\mathrm{HA}} A^{\lambda_i}_{\mathrm{A}^-} + C^{rel}_{\mathrm{HB}} A^{\lambda_i}_{\mathrm{B}^-}\right)\right]^2 \rightarrow min \tag{29}$$

The wavelength range in eq 29 is chosen so that HA and HB have a similar contribution to the overall absorbance and preferably the absorbance maxima of HA and HB (or their anions A⁻ and B⁻) are part of the wavelength range.

Substituting the concentrations of the species, optical path lengths and molar absorption coefficients from equations 23, 24, 25, 26, 27 and 28, into eq 22 one arrives at eq 30.

$$A^{\lambda} - A^{\lambda}_{\mathrm{HA}} C^{rel}_{\mathrm{HA}} - A^{\lambda}_{\mathrm{HB}} C^{rel}_{\mathrm{HB}} = \alpha_{\mathrm{HA}} \left(A^{\lambda}_{\mathrm{A}^{-}} C^{rel}_{\mathrm{HA}} - A^{\lambda}_{\mathrm{HA}} C^{rel}_{\mathrm{HA}} \right) + \alpha_{\mathrm{HB}} \left(A^{\lambda}_{\mathrm{B}^{-}} C^{rel}_{\mathrm{HB}} - A^{\lambda}_{\mathrm{HB}} C^{rel}_{\mathrm{HB}} \right) \quad (30)$$

After rearranging eq 30, a multiple linear regression equation with one dependent (y) and two independent (x_1 and x_2) variables is obtained:

$$\underbrace{A^{\lambda} - A^{\lambda}_{\mathrm{HA}} C^{rel}_{\mathrm{HA}} - A^{\lambda}_{\mathrm{HB}} C^{rel}_{\mathrm{HB}}}_{y} = \underbrace{\alpha_{\mathrm{HA}} C^{rel}_{\mathrm{HA}}}_{a} \underbrace{\left(A^{\lambda}_{\mathrm{A}^{-}} - A^{\lambda}_{\mathrm{HA}} \right)}_{x_1} + \underbrace{\alpha_{\mathrm{HB}} C^{rel}_{\mathrm{HB}}}_{b} \underbrace{\left(A^{\lambda}_{\mathrm{B}^{-}} - A^{\lambda}_{\mathrm{HB}} \right)}_{x_2} \quad (31)$$

In eq 31, the variables x_1 and x_2 are found directly from absorbance data of the neutral and fully deprotonated forms of acids HA and HB obtained from their individual titrations. The regression coefficients a and b are found for the spectrum of each mixture (i.e. et each titration point) by using the least squares minimization of the sum of squares *SSD* over a wavelength range $\lambda_1 - \lambda_n$ as shown in eq 32.

$$SSD = \sum_{i=1}^{n} \left\{ A^{\lambda_i} - A^{\lambda_i}_{\mathrm{HA}} C^{rel}_{\mathrm{HA}} - A^{\lambda_i}_{\mathrm{HB}} C^{rel}_{\mathrm{HB}} - \left[\alpha_{\mathrm{HA}} C^{rel}_{\mathrm{HA}} \left(A^{\lambda_i}_{\mathrm{A}^{-}} - A^{\lambda_i}_{\mathrm{HA}} \right) + \alpha_{\mathrm{HB}} C^{rel}_{\mathrm{HB}} \left(A^{\lambda_i}_{\mathrm{B}^{-}} - A^{\lambda_i}_{\mathrm{HB}} \right) \right] \right\}^2 \rightarrow min \quad (32)$$

Eq 31 is solved for every mixture formed during the stepwise titration of the solution containing HA and HB using the least squares minimization method in eq 32.

In the Beer-Lambert law, there is no intercept; therefore, the calculated intercept value should be equal to 0 and eq 31 represents a linear regression equation without an intercept. However, occasionally spectral disturbances can cause the intercept to become statistically significant. In such cases intercept can be brought into the equation as an additional term (b_0) as it enables to partially correct for such disturbances. In such a case the coefficients a and b can be determined from regression equation with intercept, eq 33:

$$\underbrace{A^{\lambda} - A^{\lambda}_{\mathrm{HA}} C^{rel}_{\mathrm{HA}} - A^{\lambda}_{\mathrm{HB}} C^{rel}_{\mathrm{HB}}}_{y} = \underbrace{\alpha_{\mathrm{HA}} C^{rel}_{\mathrm{HA}}}_{a} \underbrace{\left(A^{\lambda}_{\mathrm{A}^{-}} - A^{\lambda}_{\mathrm{HA}} \right)}_{x_1} + \underbrace{\alpha_{\mathrm{HB}} C^{rel}_{\mathrm{HB}}}_{b} \underbrace{\left(A^{\lambda}_{\mathrm{B}^{-}} - A^{\lambda}_{\mathrm{HB}} \right)}_{x_2} + b_0 \quad (33)$$

From eq 33 the regression coefficients a and b are found by using the least squares minimization of the sum of squares *SSD* over a wavelength range as shown in eq 34.

$$SSD = \sum_{\lambda} \left\{ A^{\lambda} - A^{\lambda}_{\mathrm{HA}} C^{rel}_{\mathrm{HA}} - A^{\lambda}_{\mathrm{HB}} C^{rel}_{\mathrm{HB}} - \left[\alpha_{\mathrm{HA}} C^{rel}_{\mathrm{HA}} \left(A^{\lambda}_{\mathrm{A}^{-}} - A^{\lambda}_{\mathrm{HA}} \right) + \alpha_{\mathrm{HB}} C^{rel}_{\mathrm{HB}} \left(A^{\lambda}_{\mathrm{B}^{-}} - A^{\lambda}_{\mathrm{HB}} \right) + b_0 \right] \right\}^2 \rightarrow min \quad (34)$$

Regression analysis is always carried out both ways - by solving eq 31 and eq 33. The obtained a and b values are then used to calculate the degrees of dissociation of HA and HB in the mixture.

$$\alpha_{\mathrm{HA}} = \frac{a}{c_{\mathrm{HA}}^{rel}} = \frac{a}{a_{\mathrm{A}^-}} \tag{35}$$

$$\alpha_{\mathrm{HB}} = \frac{b}{c_{\mathrm{HB}}^{rel}} = \frac{b}{b_{\mathrm{B}^-}} \tag{36}$$

In practice, α_{HB} and α_{HB} are calculated using the following equations:

$$\alpha_{\mathrm{HA}} = \frac{a - a_{\mathrm{HA}}}{a_{\mathrm{A}^-} - a_{\mathrm{HA}}} \tag{37}$$

$$\alpha_{\mathrm{HB}} = \frac{b - b_{\mathrm{HB}}}{b_{\mathrm{B}^-} - b_{\mathrm{HB}}} \tag{38}$$

In equations 35–38 a_{A}- and b_{B}- are the regression coefficients obtained from the spectra where HA and HB are in their anionic (fully deprotonated) forms A^- and B^-; a_{HA} and b_{HB} are the regression coefficients calculated from the spectra of the neutral forms HA and HB. Theoretically, a_{HA} and b_{HB} in equations 37 and 38 should equal 0.

The above-described derivation is only valid if the analytical concentrations of HA and HB are constant in every recorded spectrum. In practice, the concentrations decrease after each addition of a titrant solution used to deprotonate HA and HB because of the increase in solution volume inside the cuvette. To account for the decrease in concentration of HA and HB, every absorbance value in a recorded spectrum is multiplied by a volume correction factor cf_{Vol}.

$$cf_{Vol} = \frac{(m_{init} + m_{added})}{m_{init}} \tag{39}$$

In equation 39, m_{init} is the initial mass of the solution inside the cuvette before the addition of any titrants, and m_{added} is the total mass of the added titrant solution before the recording of each spectrum.

The obtained degrees of dissociation are used in eq 17 to calculate the ΔpK_a value for the acid pair HA and HB. Equation 31 and eq 33 are solved for every mixture with different degrees of dissociation formed during the titration of the mixture. Therefore, a ΔpK_a value can be calculated for every recorded spectrum of the mixture of acids HA and HB. An average ΔpK_a value assigned to the acid pair HA and HB (and presented in the "ladder" in the main text) is calculated from ΔpK_a values that correspond to α values between 0.1 and 0.9.

pK_a measurements of benzotriazole

In addition to the pK_a values of the very weak acids shown in **Table 1** of the main text, the pK_a value of benzotriazole was determined. The results of the individual relative pK_a measurements against three different reference compounds are presented in **Table S1**.

Table S1. Benzotriazole pK_a measurement results in MeCN.

Reference acid (RA)	pK_a(RA)	ΔpK_a	pK_a	Assigned pK_a
3,5-$(CF_3)_2$-C_6H_3-CH_2-SO_2CF_3	22.84	-0.13	22.97	**22.98**
(4-Me-C_6F_4)(C_6F_5)CHCN	21.94	-1.04	22.98	
4-CN-C_6H_4-CH_2- SO_2CF_3	23.19	0.22	22.97	

Additional computational GA values

Reference acids

The computational gas-phase acidities (GA) of the reference acids (acids with previously known pK_a(MeCN) values) used to determine the pK_a values of the compounds from **Table 1** of the main text are presented in **Table S2**.

Table S2. Experimental and computational GA values of reference acids.[a]

Name	CAS No	pK_a(MeCN)	GA(calc) [kJ mol^{-1}]	GA(exp) [kJ mol^{-1}]
4-CN-2,3,5,6-F_4-Aniline	17823-38-0	28.78	1378	1365[2]
C_6H_5-CH_2-SO_2CF_3	4855-02-1	27.59	1388	1251[b,3]
4-CN-C_6H_4-CH_2-SO_2CF_3	73622-52-3	23.19	1336	
(4-Me-C_6F_4)(C_6H_5)CHCN	58432-62-5	26.98	1384	1373[4]
(4-Me-C_6F_4)(C_6F_5)CHCN	52345-34-3	21.94	1333	1323[4]
9-C_6F_5-Fluorene	73482-93-6	28.14	1382	
3,5-$(CF_3)_2$-C_6H_3-CH_2-SO_2CF_3	434957-15-0	22.84	1334	1310.8[5]
t$Bu_4Box_2CH_2$	2128672-84-2	28.85	1384	

[a]All pK_a(MeCN) and GA(calc) values are from this work. [b]Stated source of the value is personal communication with R.W. Taft from August 1991. Probably erroneous value because it cannot be lower than the GA of $(CF_3SO_2)_2CH$-C_6H_5 (1255 kJ/mol)[2].

Isomers of 1,2,3-triazole and 1,2,3-benzotriazole

Table S3. The computational and experimental GA values of some triazole and benzotriazole isomers.

**Name**	**CAS No**	**GA(calc) [kJ mol^{-1}]**	**GA(exp) [kJ mol^{-1}]**
1,2,4-triazole	288-88-0	1419	1410[a3]
2*H*-1,2,3-triazole	288-35-7	1427	
2*H*-1,2,3-benzotriazole	273-02-9	1380	

[a]Stated source of the value is personal communication with R.W. Taft from August 1991. The value is probably erroneous.

Acetonitrile for pK_a determinations

For the majority of relative acidity measurements to build the pK_a scale of acids in MeCN, acetonitrile produced by Romil (190 SpS far UV/gradient quality) has been used without further purification.[6] The only treatment that has been done was drying the solvent on 3 Å molecular sieves to ensure a water content under 10 ppm. During the pK_a measurements of nitro-substituted anilines, it was observed that during the deprotonation, a side process occurred that altered the UV-Vis spectra and caused the loss of sharpness of the isosbestic points. An example with 4-NO_2-aniline is presented in Figure S1. If distilled MeCN was used, the UV-Vis spectra registered during the titration looked normal. This unknown side process only appeared in the spectra of nitro-substituted anilines and was absent in 2,3,5,6-Cl_4-aniline and 2,3,4,5,6-Cl_5-aniline. This was confirmed by measuring the relative acidity of this compound pair in both the distilled MeCN and the undistilled MeCN. The spectra visually looked the same, and the result was essentially the same (ΔpK_a differed by 0.04). Additionally, the pair *t*$Bu_4Box_2CH_2$ and 4-CN-2,3,5,6-F_4-aniline, previously measured[6] in undistilled MeCN, was remeasured using distilled MeCN. Again, essentially, the same result (ΔpK_a differed by 0.02) was obtained. This confirmed that the unknown side process was present only in the case of nitro-substituted anilide anions.

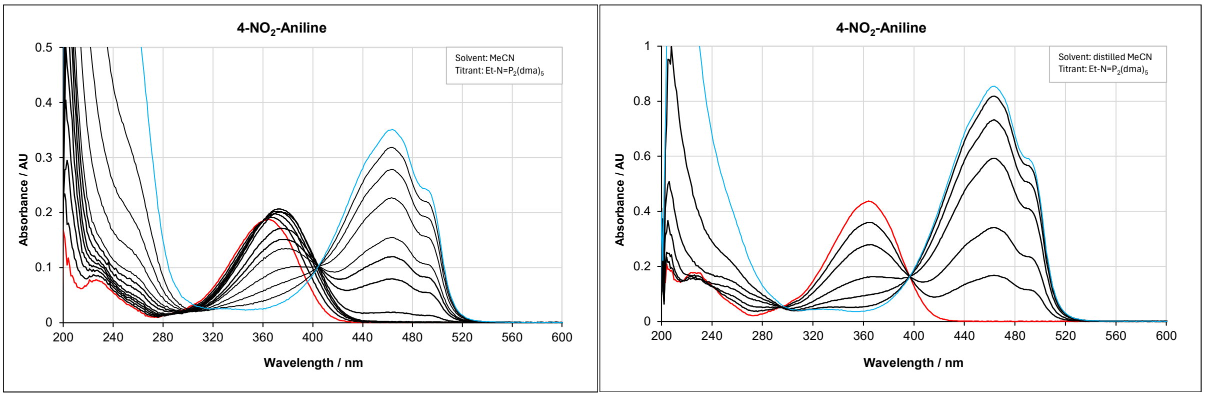

Figure S1. Comparison of the UV-Vis deprotonation spectra of 4-NO_2-aniline in non-distilled MeCN (dried with molecular sieves) (left) and distilled MeCN (right). The rise in absorbance under 320 nm is caused by the excess of the titrant used for deprotonation.

HN=P_1(tmg)$_3$ UV-Vis spectrum

Figure S2 shows the UV-Vis titration spectra of HN=P_1(tmg)$_3$ ($7.3\cdot10^{-3}$ mol L^{-1}) with methanesulfonic acid. The spectra show that HN=P_1(tmg)$_3$ cannot be used as a basic titrant for the pK_a determinations of compounds which have absorbances only under 290 nm when using the pK_a determination method described in this paper.

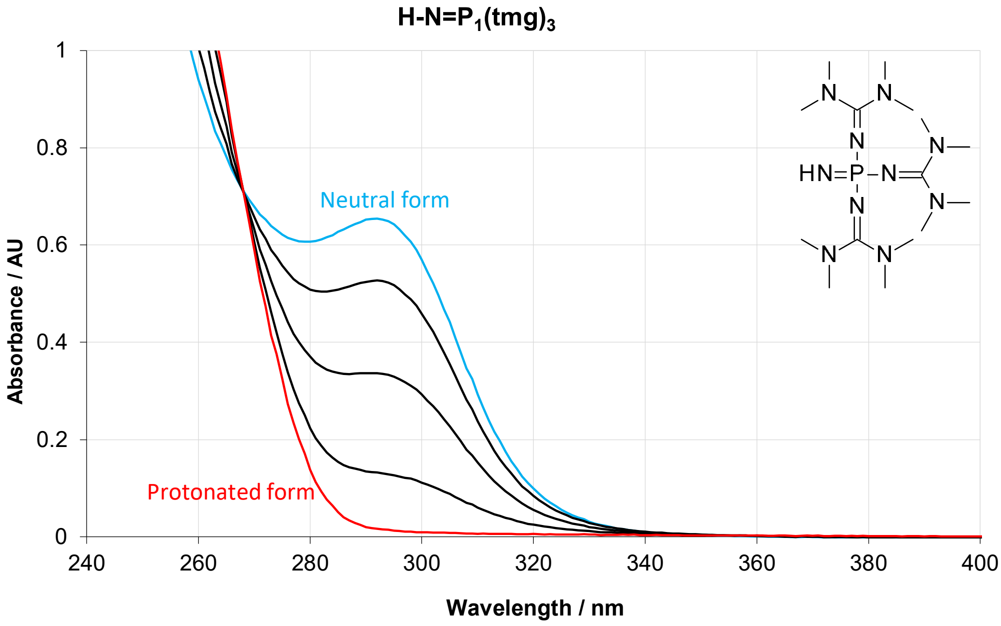

Figure S2. UV-Vis spectra of protonated (red) and neutral (blue) forms of HN=P_1(tmg)$_3$. Black spectra are partially protonated forms.

Unsuccessful pK_a determinations

To further expand the pK_a scale of acids in MeCN an attempt was made to determine the pK_a values of two CH acids C_6H_5-CH_2-CN (phenylacetonitrile) and 4-NO_2-toluene. This attempt failed because during the deprotonation of these compounds at one point the anion started to decompose. **Figure S3** a) shows the deprotonation spectra of phenylacetonitrile. The red spectrum is the spectrum of the neutral, black spectra are spectra of the compounds at different degrees of dissociation. The yellow spectra with solid lines show that during the addition of the basic titrant at one point the absorbance started to decrease which could refer to the decomposition of the compound or the presence of a side processes. The spectra with dashed yellow lines are spectra are measured after 5 minutes without an addition of the basic titrant showing that the anion is not stable in time. **Figure S3** b) shows the deprotonation of 4-NO_2-toluene. The yellow spectrum is recorded 5 minutes after the last addition of the basic titrant. The absorbance increased, which could mean that the deprotonation in this example is slow. After another 5 minutes of wait the absorbance slightly decreased. In conclusion, no clear titration endpoint could be determined for this compound.

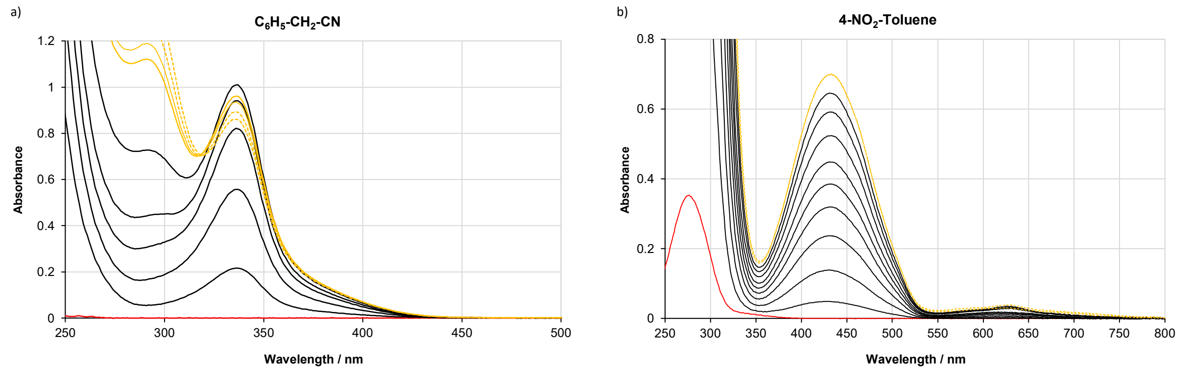

Figure S3. UV-Vis spectra of a) C_6H_5-CH_2-CN and b) 4-NO_2-toluene.

References

(1) Hammett, L. P.; Deyrup, A. J. A Series of Simple Basic Indicators. I. The Acidity Functions of Mixtures of Sulfuric and Perchloric Acids with Water. *J. Am. Chem. Soc.* **1932**, *54* (7), 2721–2739. https://doi.org/10.1021/ja01346a015.

(2) Koppel, I. A.; Taft, R. W.; Anvia, F.; Zhu, S.-Z.; Hu, L.-Q.; Sung, K.-S.; DesMarteau, D. D.; Yagupolskii, L. M.; Yagupolskii, Y. L. The Gas-Phase Acidities of Very Strong Neutral Bronsted Acids. *J. Am. Chem. Soc.* **1994**, *116* (7), 3047–3057. https://doi.org/10.1021/ja00086a038.

(3) *NIST Chemistry WebBook, NIST Standard Reference Database 69*; Linstrom, P. J., Mallard, W. G., Eds.; National Institute of Standards and Technology: Gaithersburg MD, 20899, https://doi.org/10.18434/T4D303.

(4) Koppel, I. A.; Koppel, J.; Pihl, V.; Leito, I.; Mishima, M.; Vlasov, V. M.; Yagupolskii, L. M.; Taft, (The Late) Robert W. Comparison of Brønsted Acidities of Neutral CH Acids in Gas Phase and Dimethyl Sulfoxide. *J. Chem. Soc., Perkin Trans. 2* **2000**, No. 6, 1125–1133. https://doi.org/10.1039/b001792m.

(5) Zhang, M.; Badal, M. M. R.; Koppel, I. A.; Mishima, M. Gas-Phase Acidities of α- and α,α-SO2CF3-Substituted Toluenes. Varying Resonance Demand in the Electron-Rich System. *Bulletin of the Chemical Society of Japan* **2013**, *86* (7), 813–820. https://doi.org/10.1246/bcsj.20130052.

(6) Kütt, A.; Tshepelevitsh, S.; Saame, J.; Lõkov, M.; Kaljurand, I.; Selberg, S.; Leito, I. Strengths of Acids in Acetonitrile. *Eur. J. Org. Chem.* **2021**, *2021* (9), 1407–1419. https://doi.org/10.1002/ejoc.202001649.

UV-Vis titration spectra

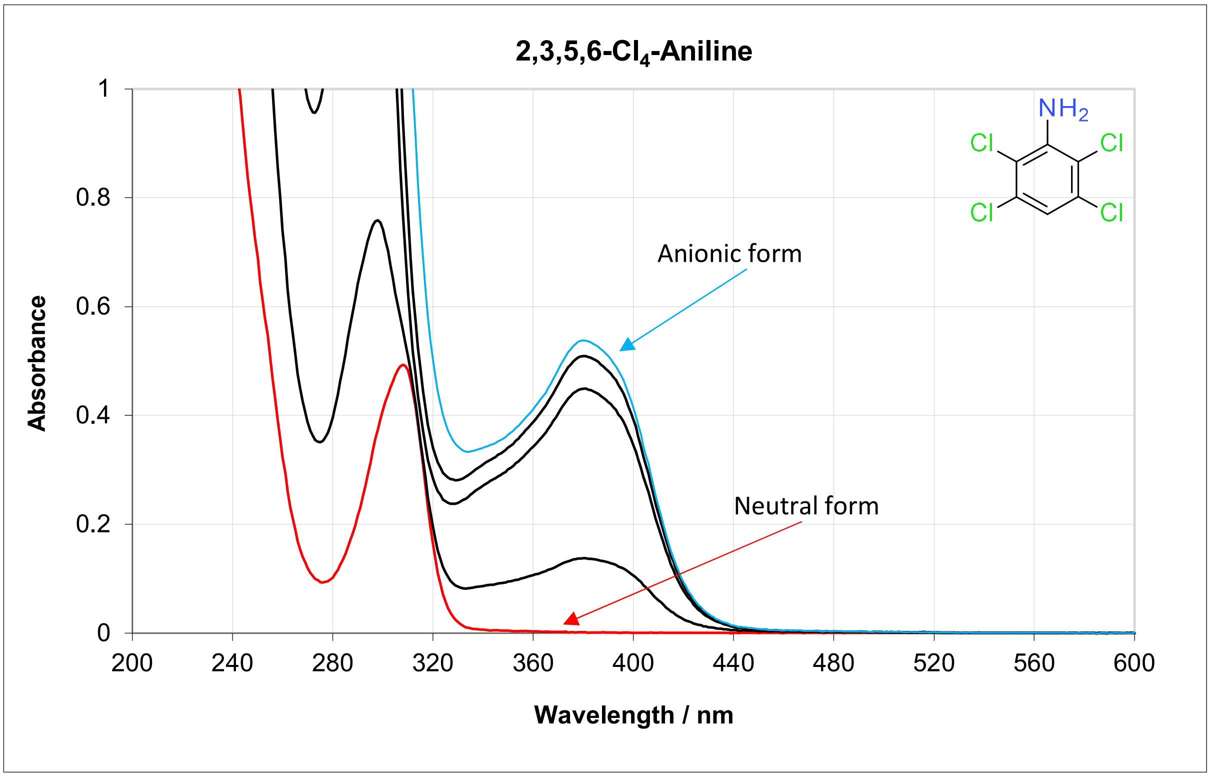

Figure S4. UV-Vis deprotonation spectra of 2,3,5,6-Cl_4-aniline in MeCN.

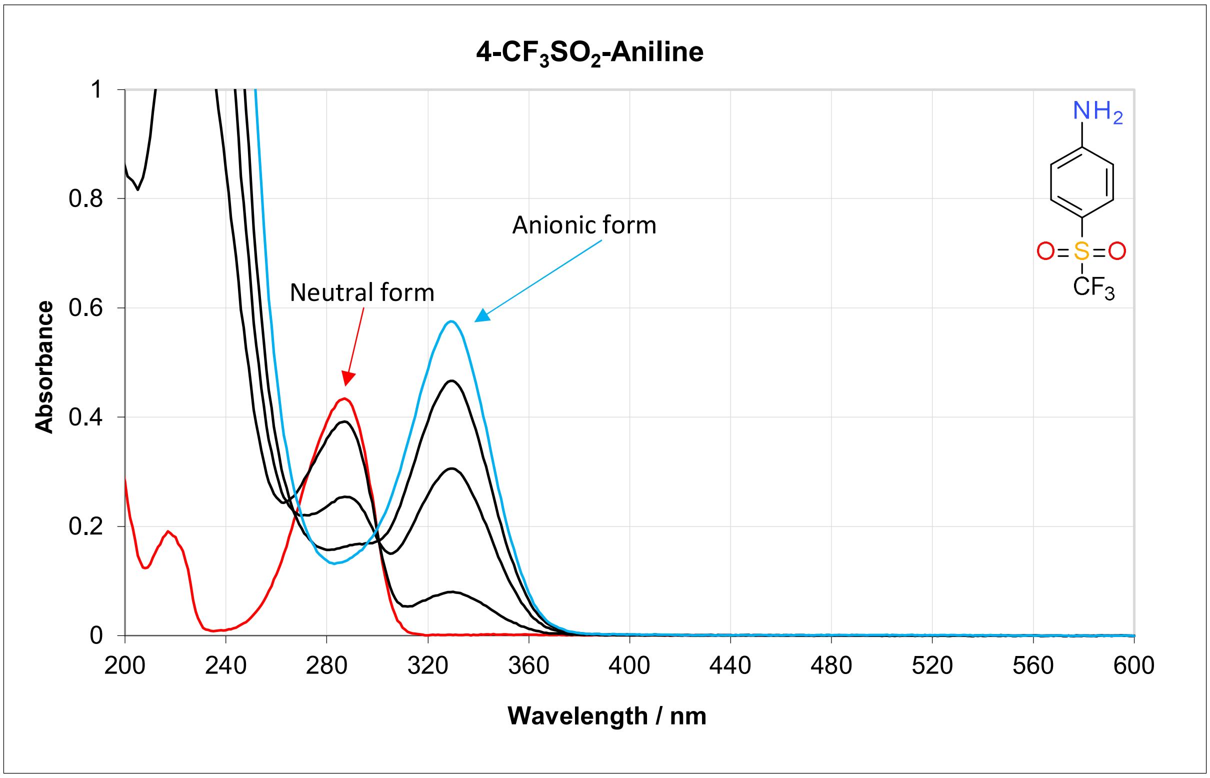

Figure S5. UV-Vis deprotonation spectra of 4-CF_3SO_2-aniline in MeCN.

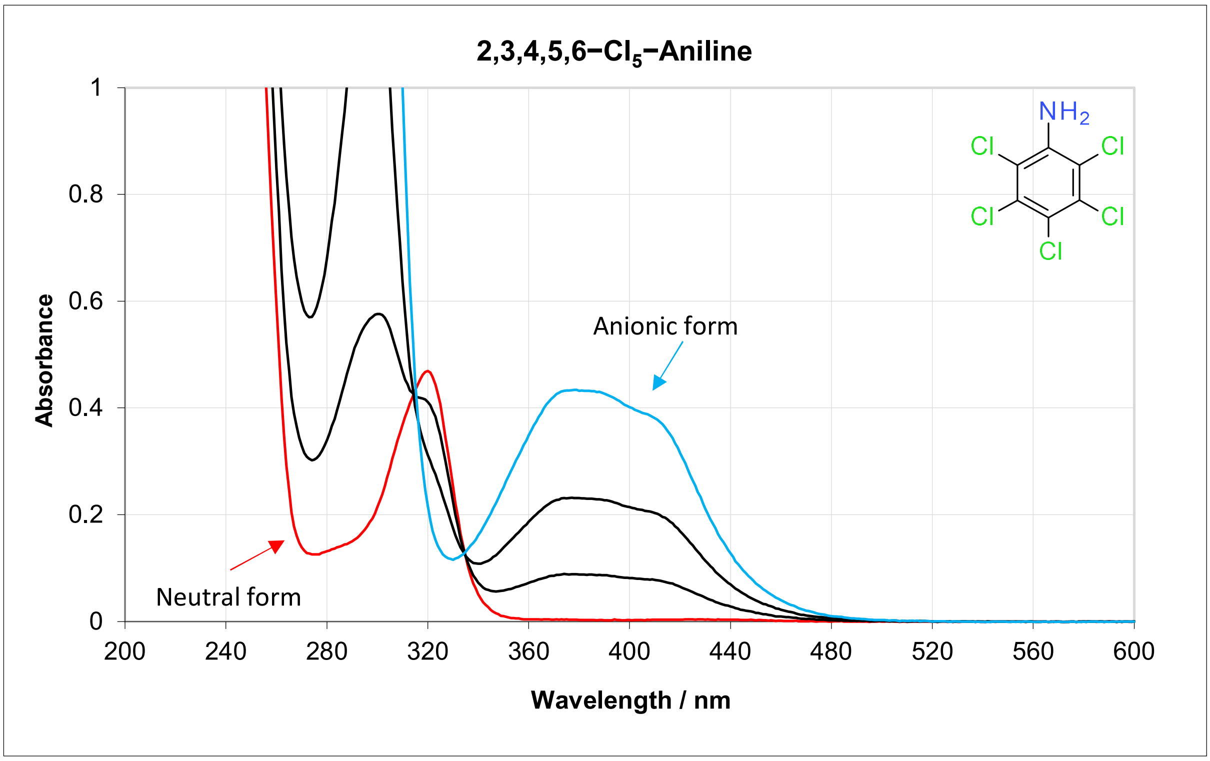

Figure S6. UV-Vis deprotonation spectra of 2,3,4,5,6-Cl_5-aniline in MeCN.

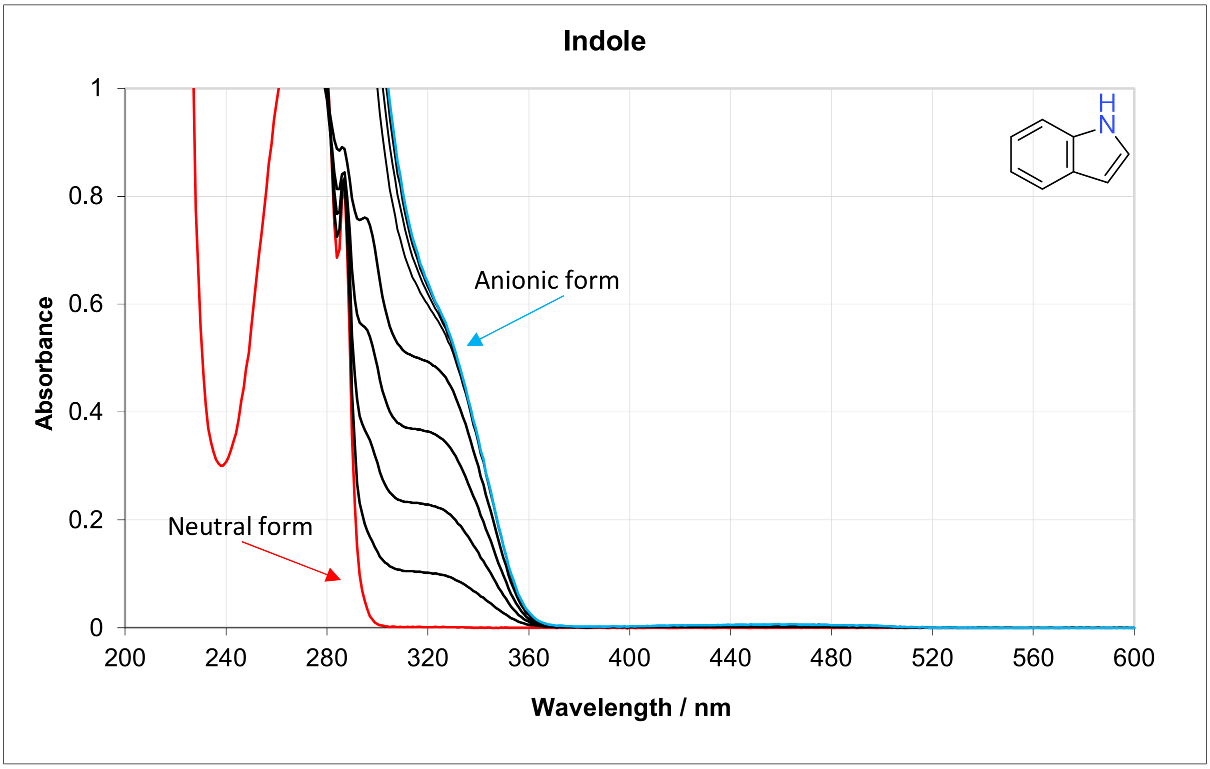

Figure S7. UV-Vis deprotonation spectra of indole in MeCN.

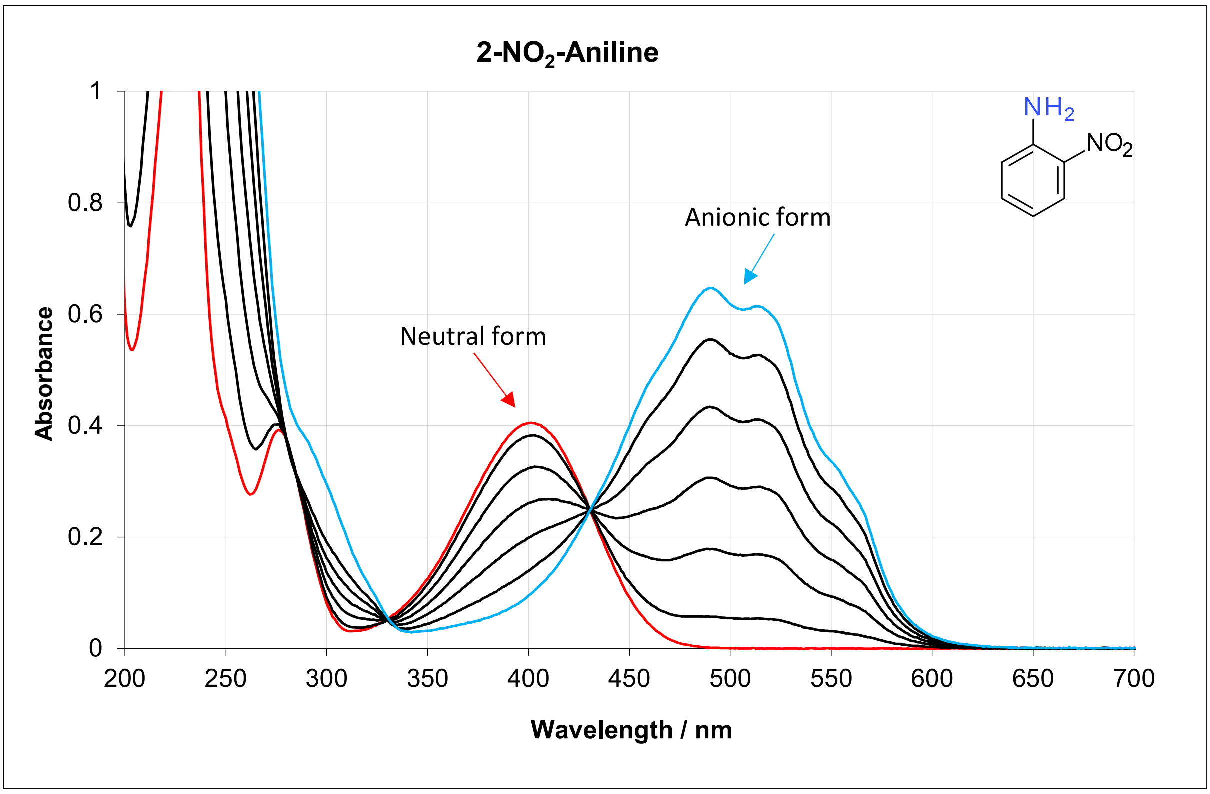

Figure S8. UV-Vis deprotonation spectra of 2-NO_2-aniline in MeCN.

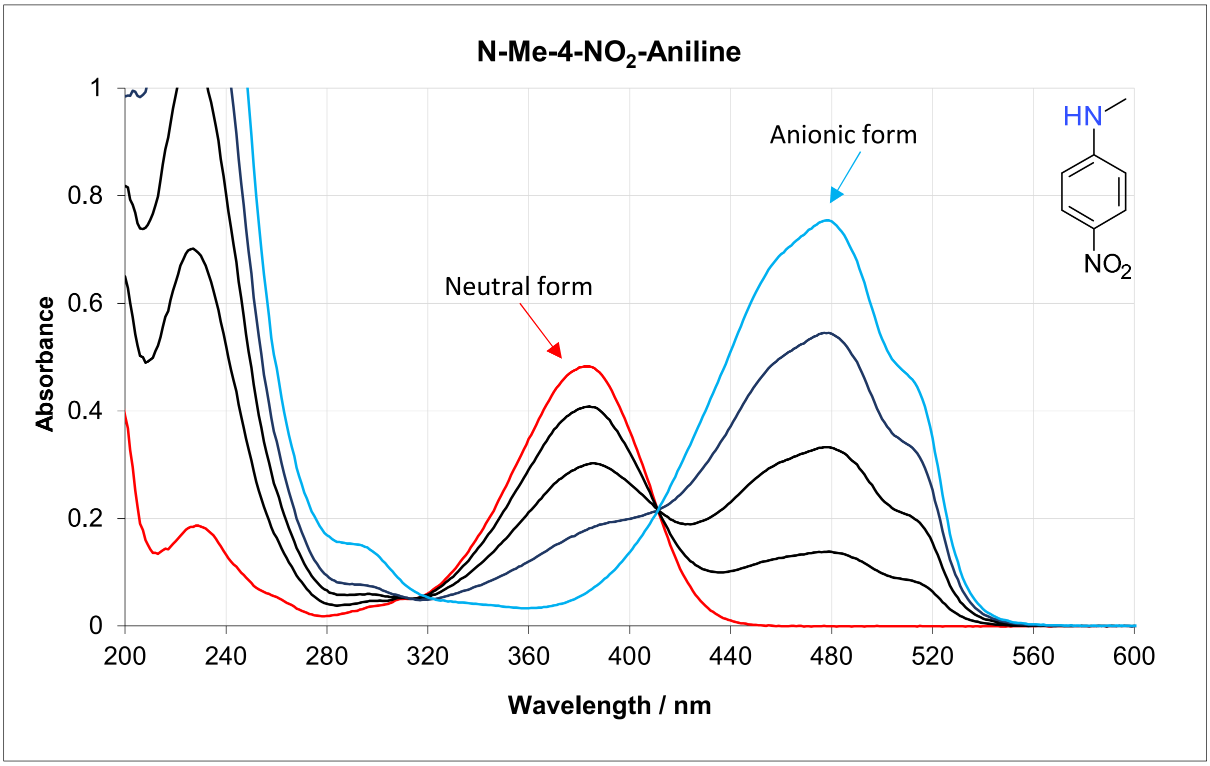

Figure S9. UV-Vis deprotonation spectra of N-Me-4-NO_2-aniline in MeCN.

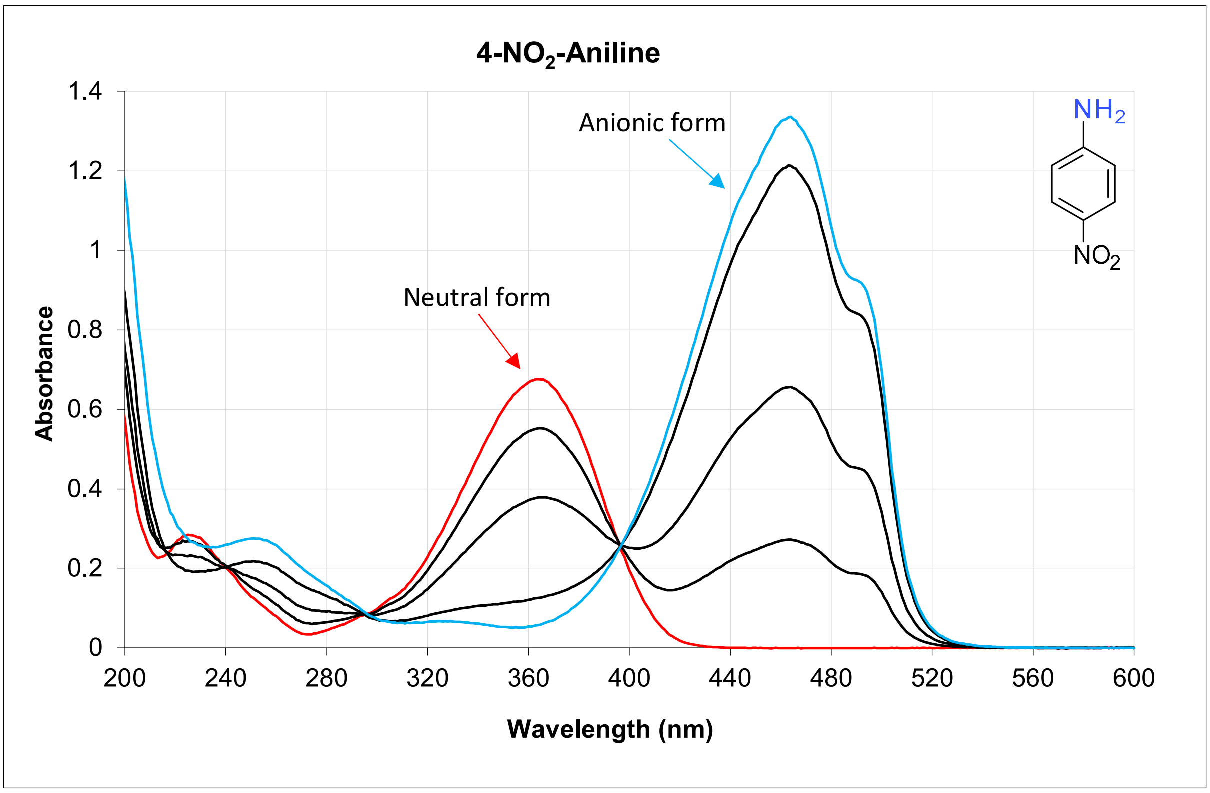

Figure S10. UV-Vis deprotonation spectra of 4-NO_2-aniline in MeCN.

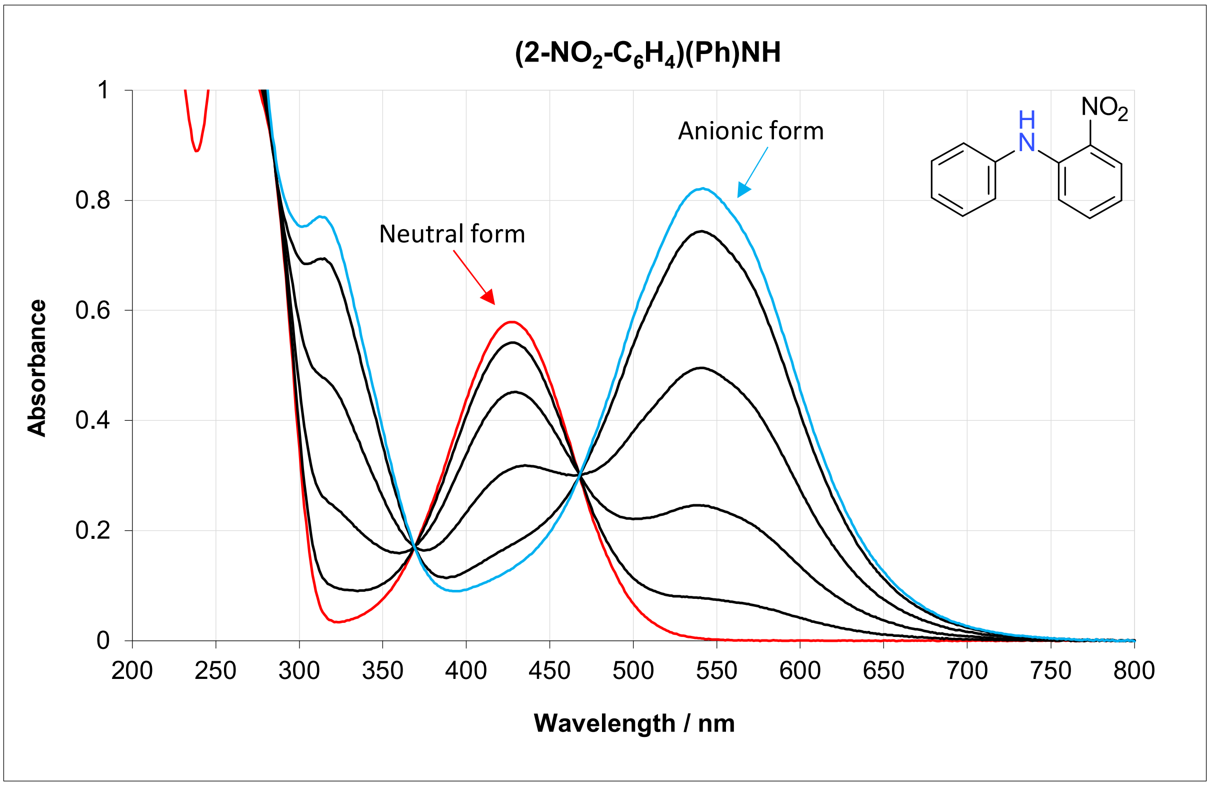

Figure S11. UV-Vis deprotonation spectra of (2-NO_2-C_6H_4)(Ph)NH in MeCN.

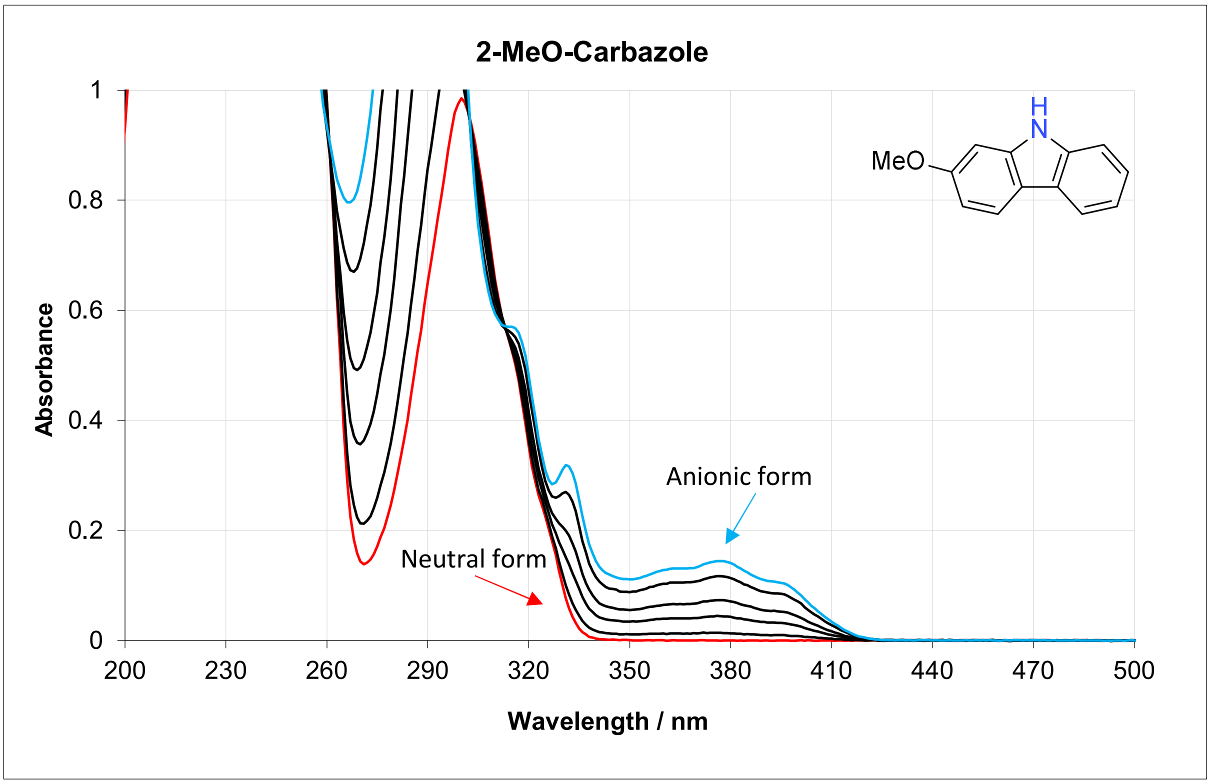

Figure S12. UV-Vis deprotonation spectra of 2-MeO-carbazole in MeCN.

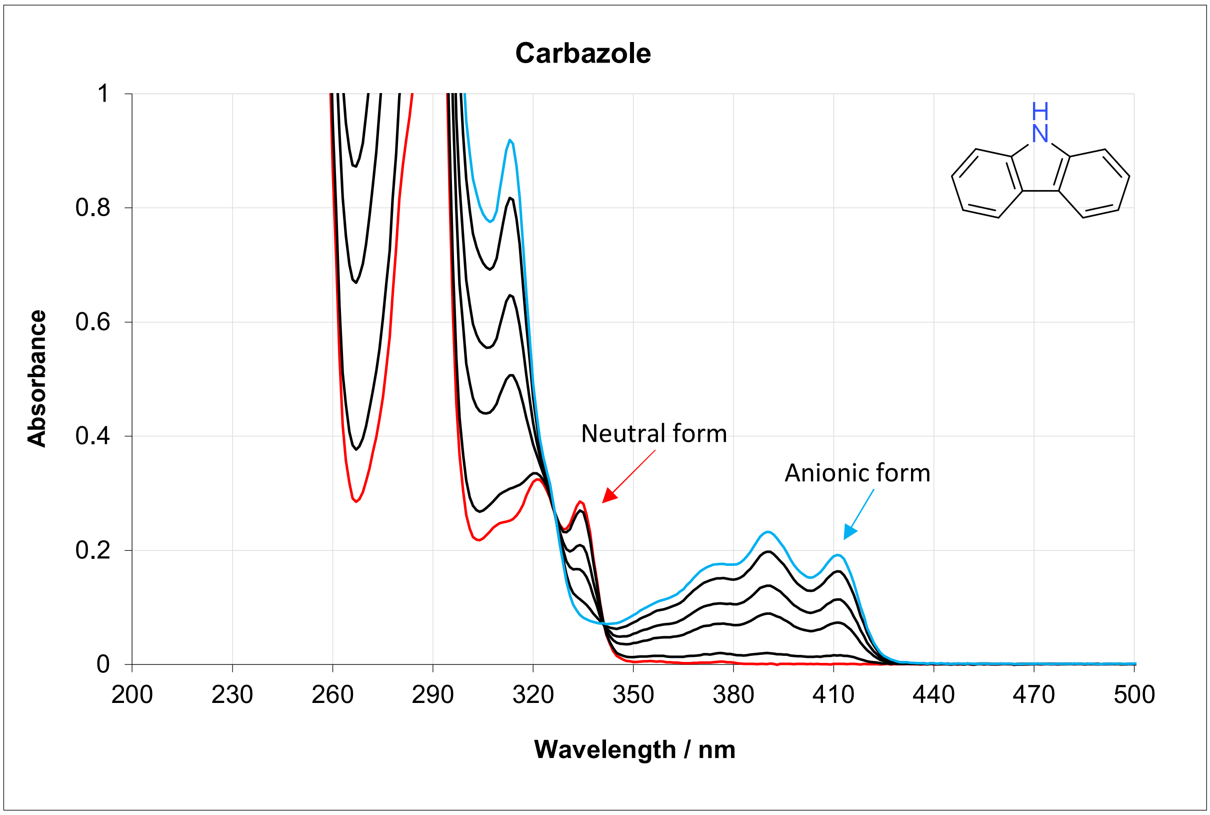

Figure S13. UV-Vis deprotonation spectra of carbazole in MeCN.

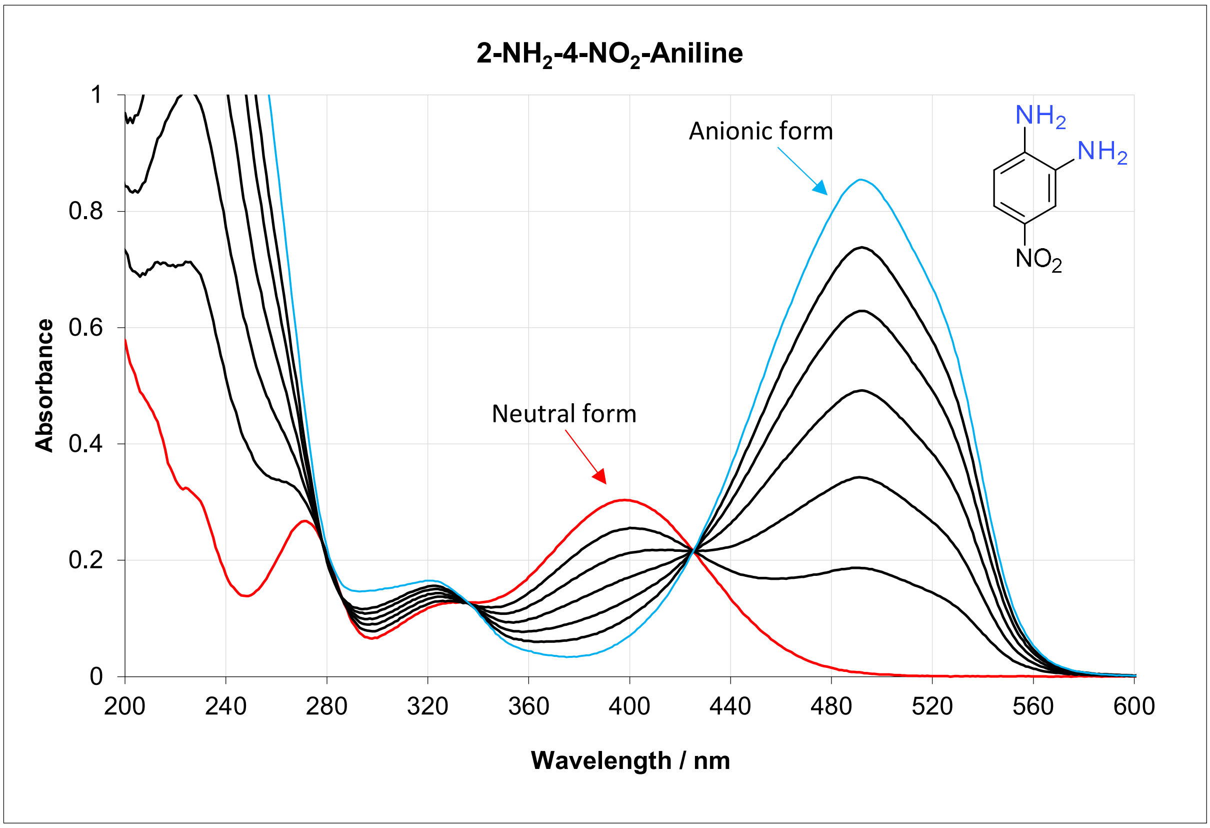

Figure S14. UV-Vis deprotonation spectra of 2-NH$_2$-4-NO$_2$-aniline in MeCN.

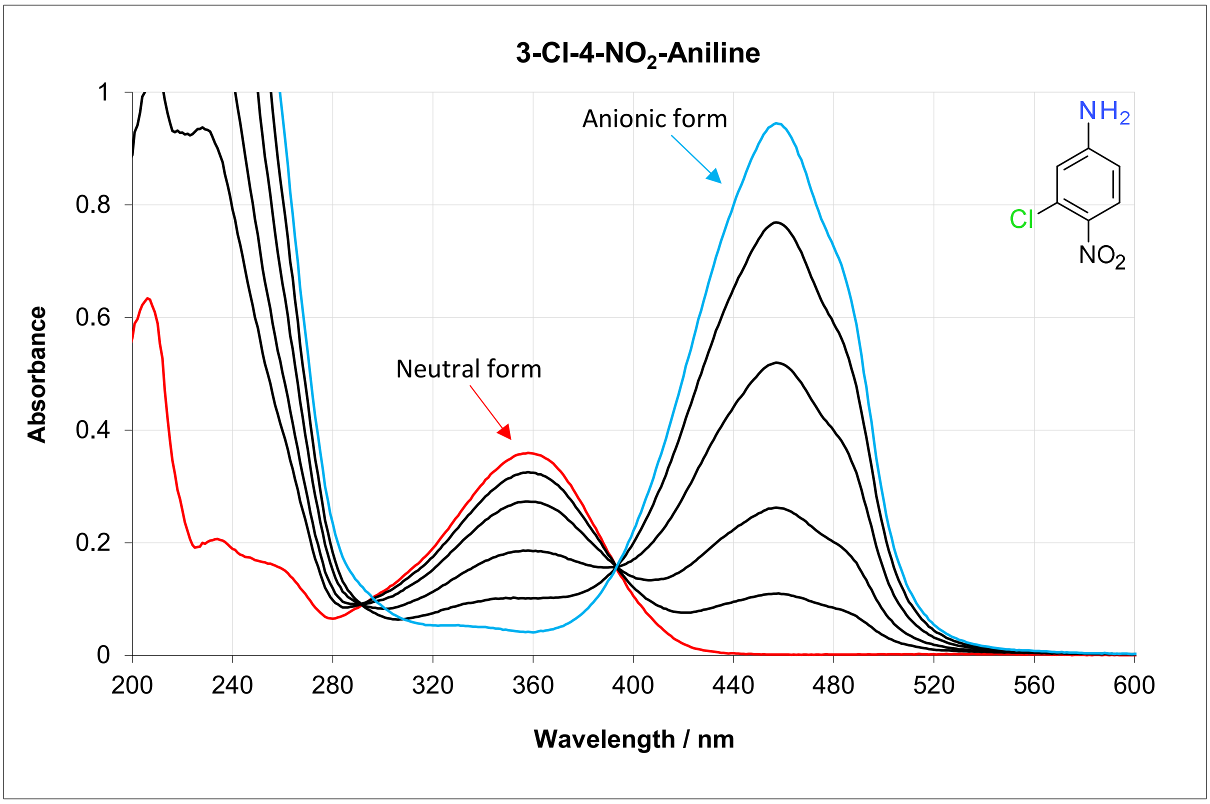

Figure S15. UV-Vis deprotonation spectra of 3-Cl-4-NO$_2$-aniline in MeCN.

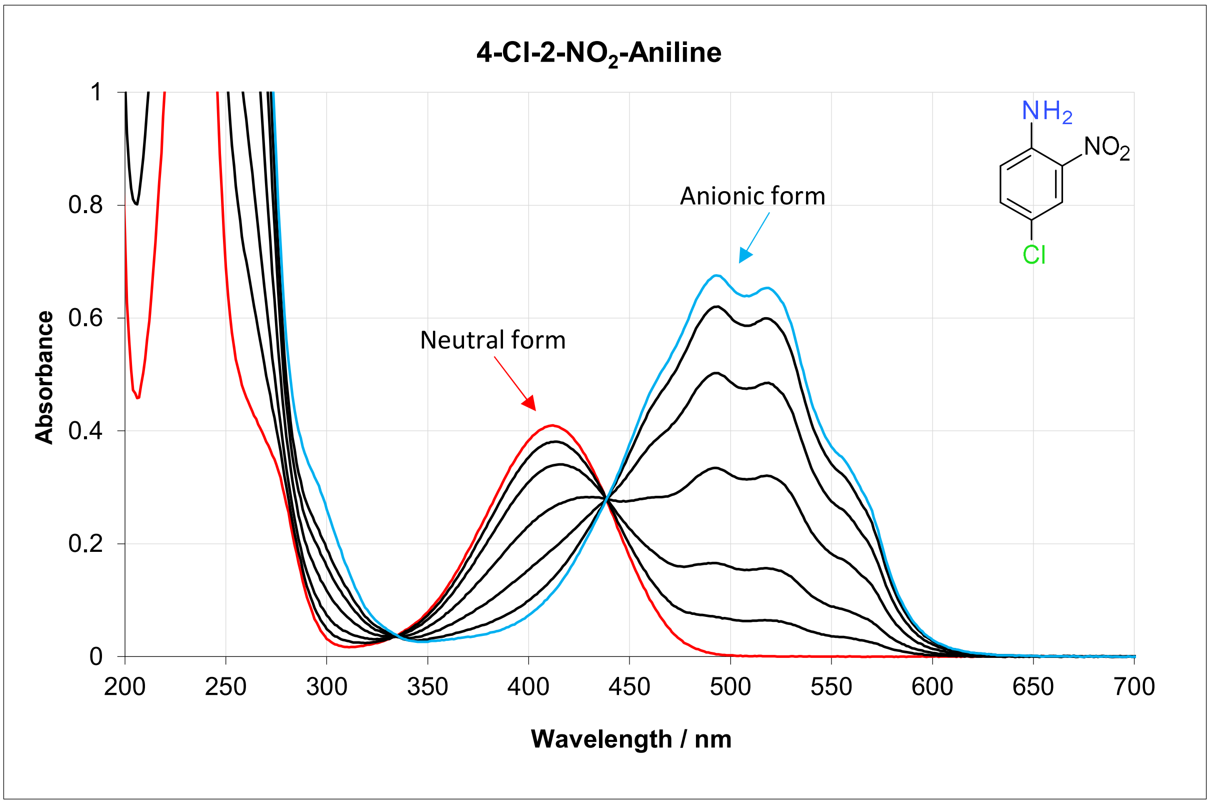

Figure S16. UV-Vis deprotonation spectra of 4-Cl-2-NO_2-aniline in MeCN.

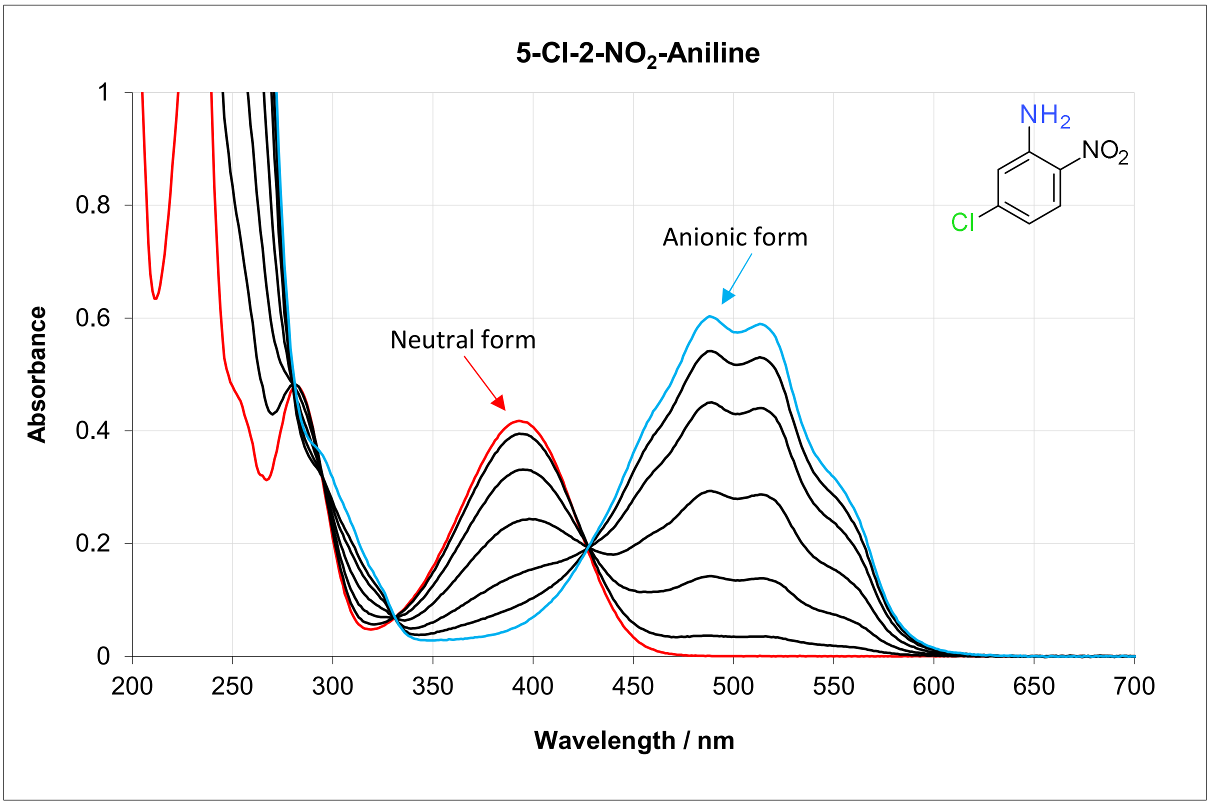

Figure S17. UV-Vis deprotonation spectra of 5-Cl-2-NO_2-aniline in MeCN.

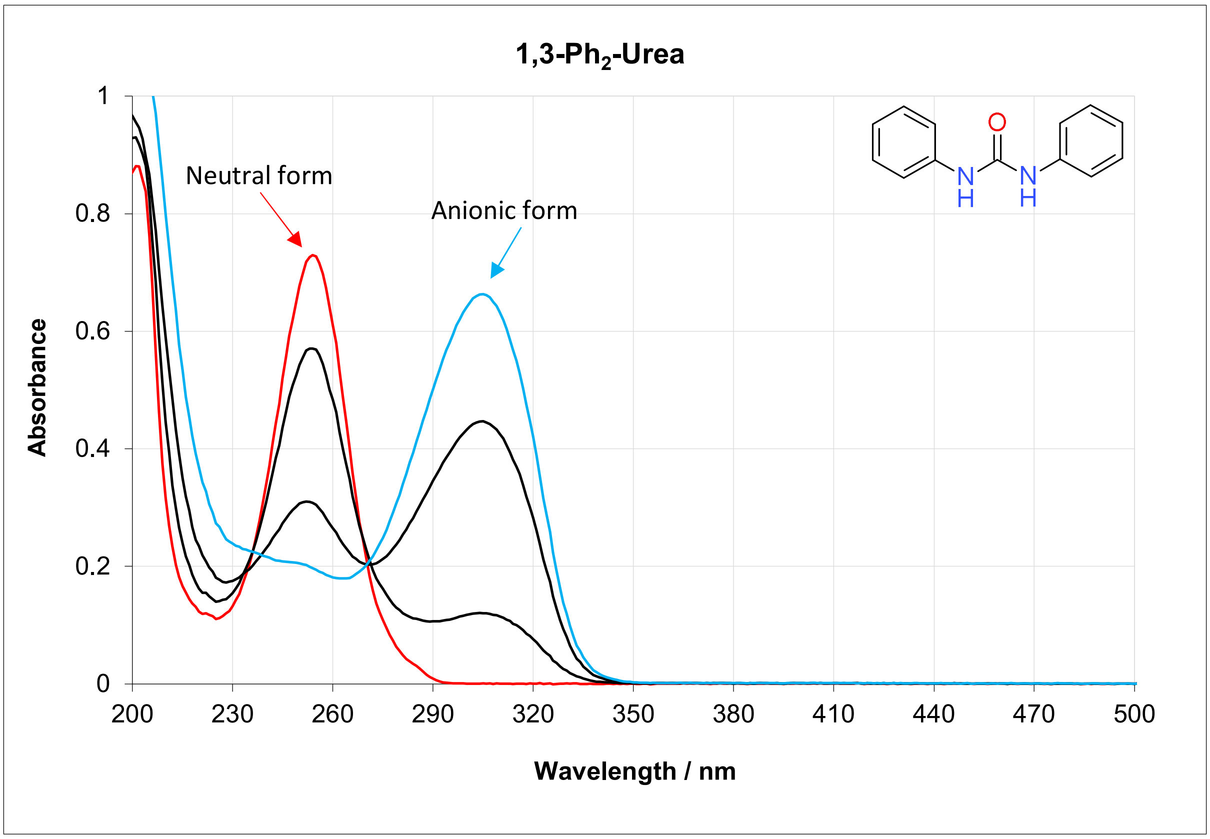

Figure S18. UV-Vis deprotonation spectra of 1,3-Ph$_2$-urea in MeCN.

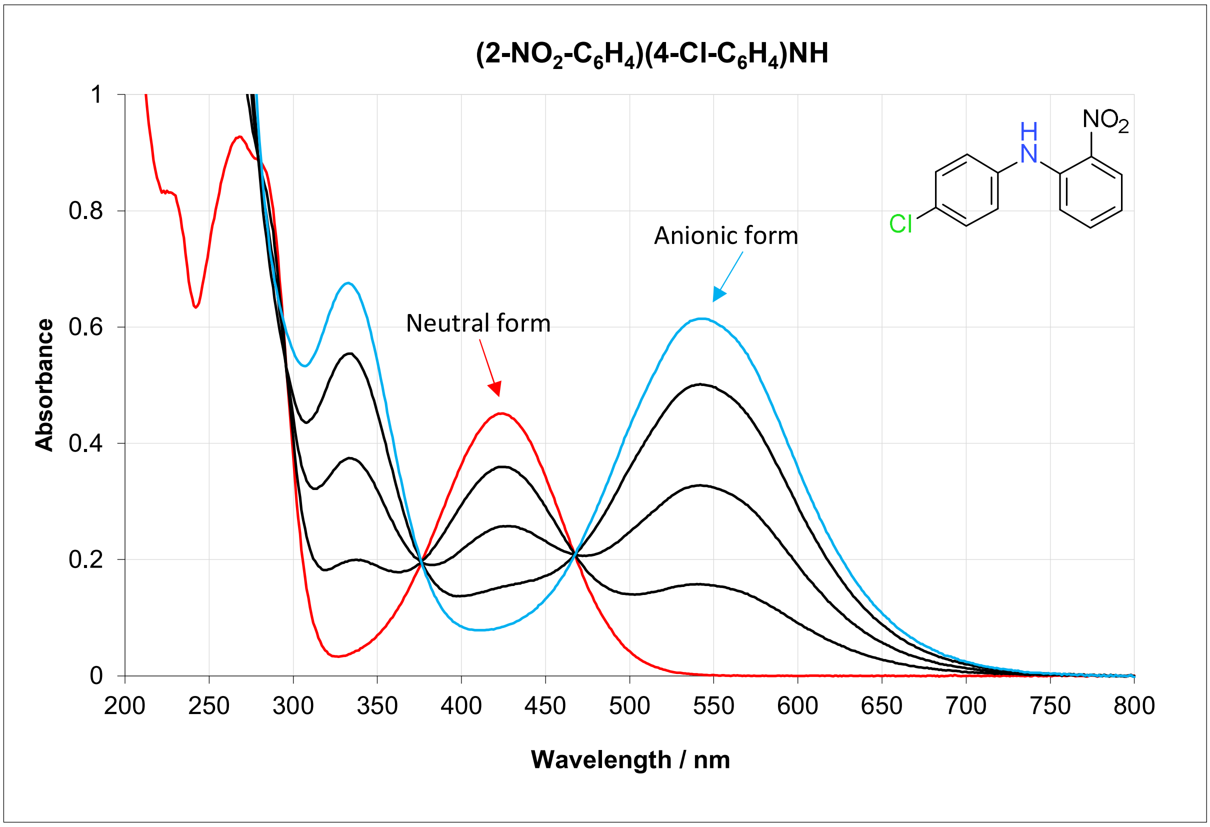

Figure S19. UV-Vis deprotonation spectra of (2-NO$_2$-C$_6$H$_4$)(4-Cl-C$_6$H$_4$)NH in MeCN.

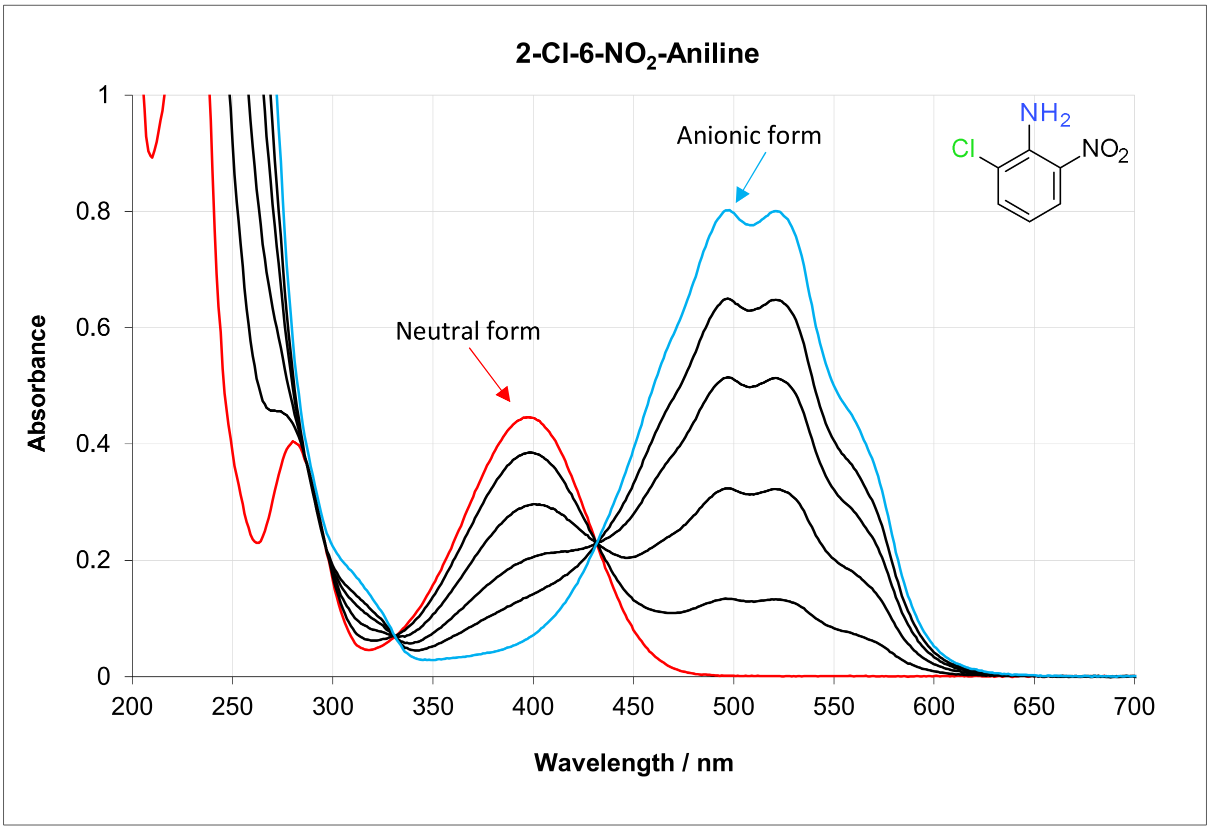

Figure S20. UV-Vis deprotonation spectra of 2-Cl-6-NO_2-aniline in MeCN.

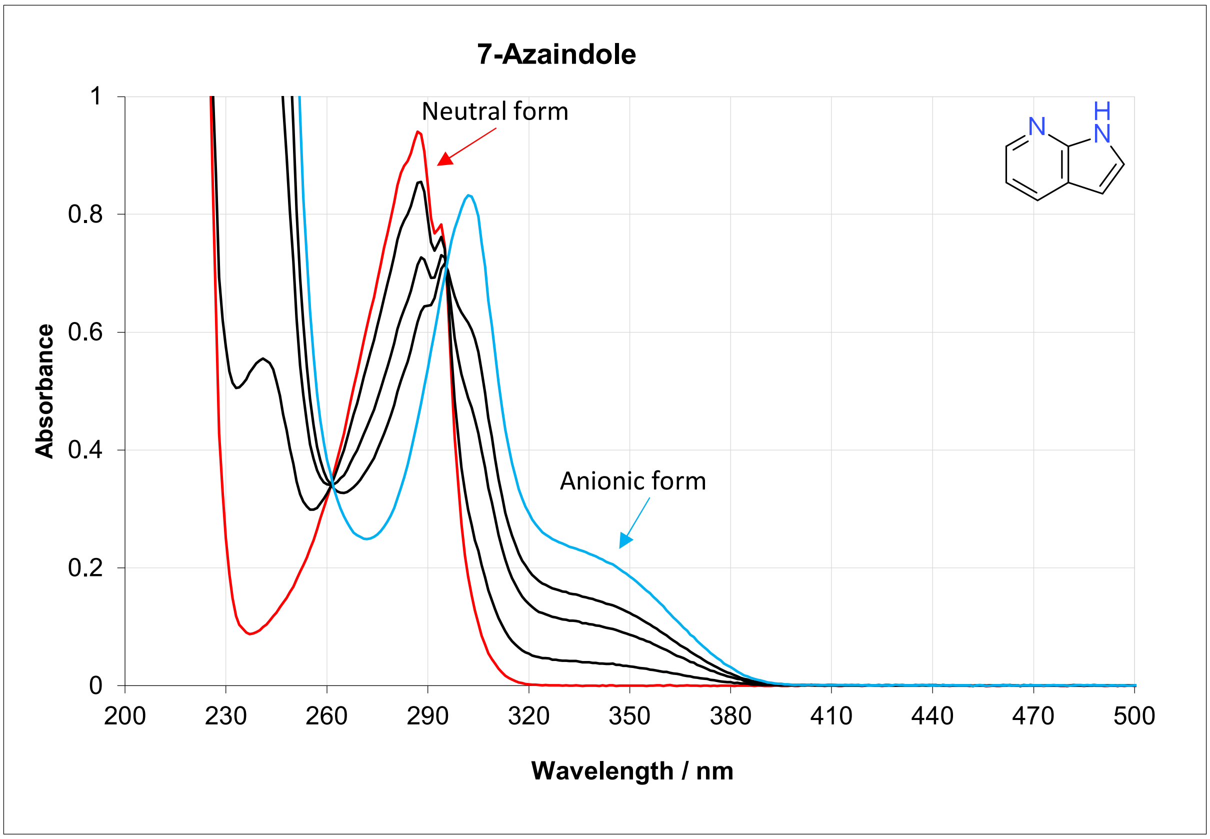

Figure S21. UV-Vis deprotonation spectra of 7-azaindole in MeCN.

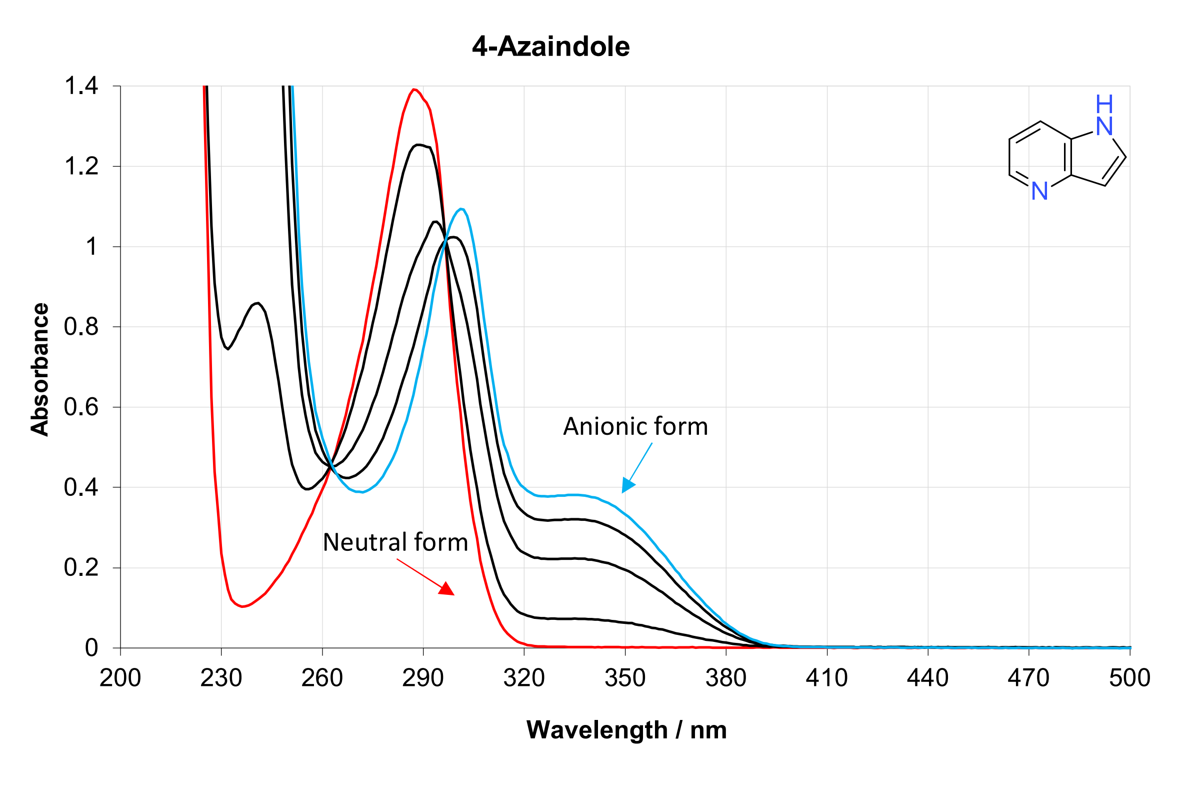

Figure S22. UV-Vis deprotonation spectra of 4-azaindole in MeCN.

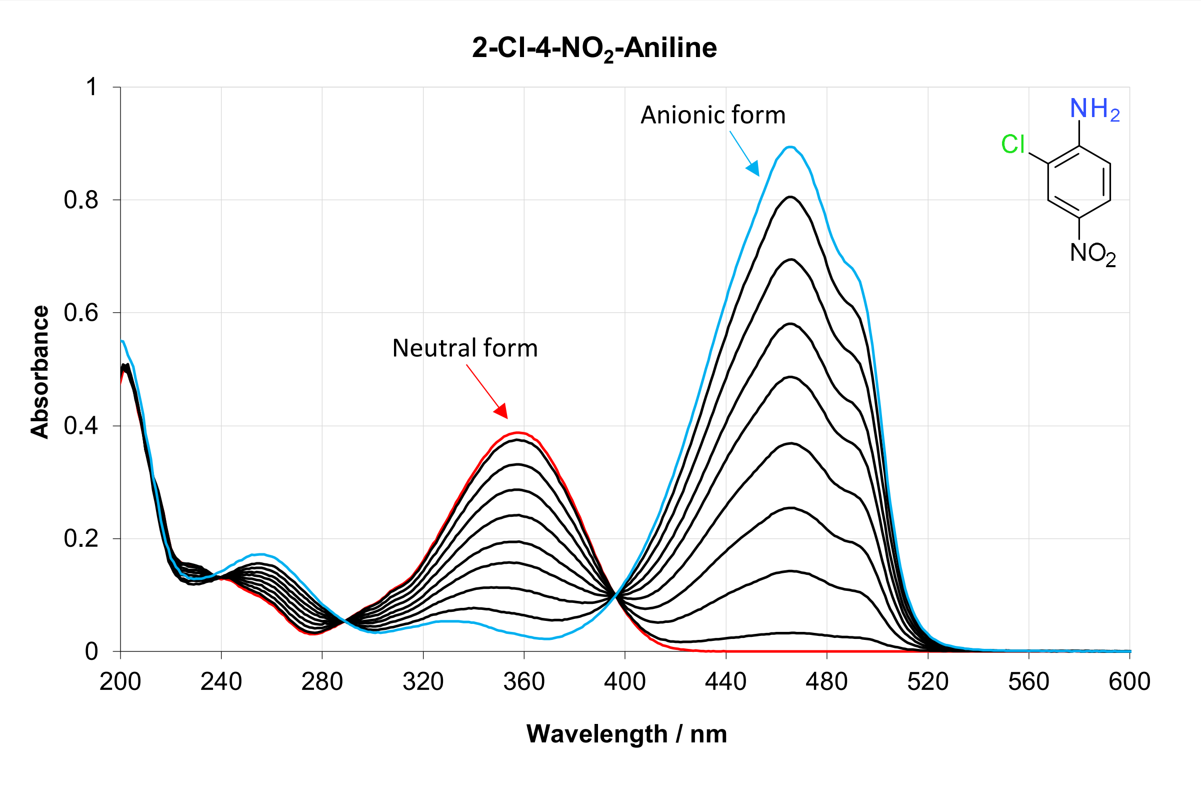

Figure S23. UV-Vis deprotonation spectra of 2-Cl-4-NO_2-aniline in MeCN.

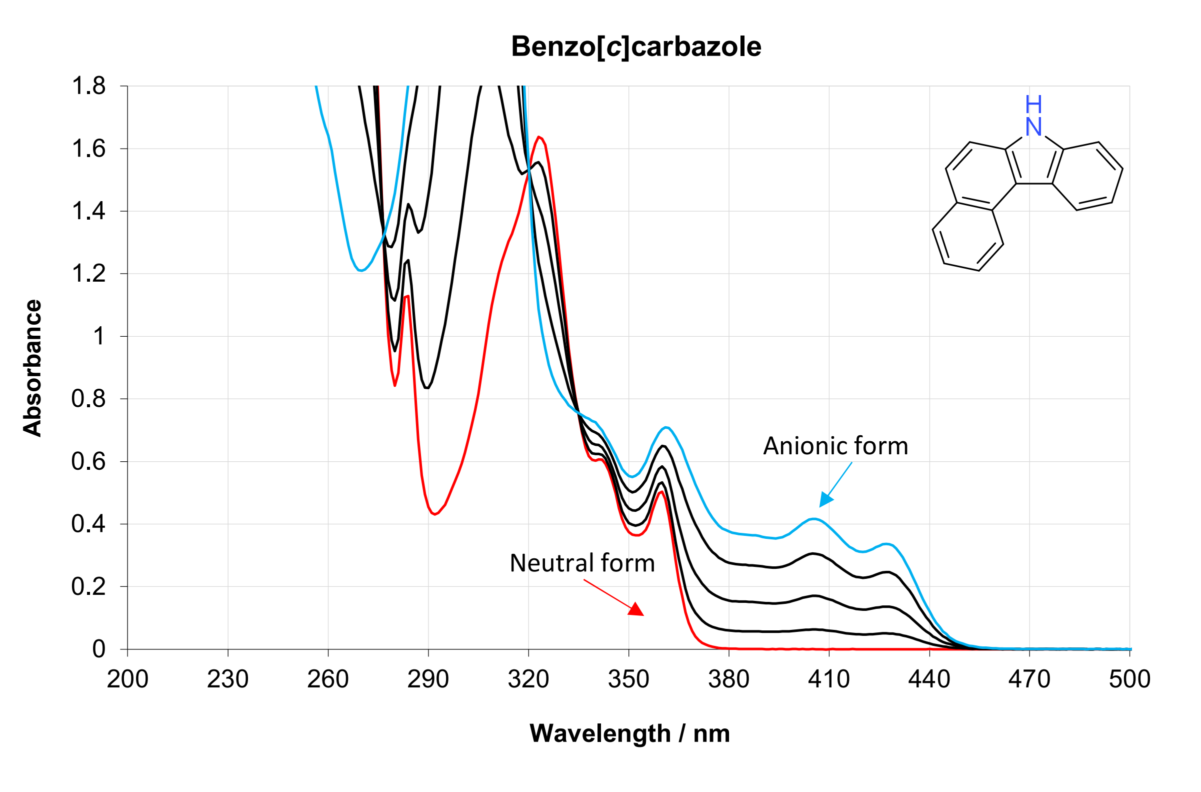

Figure S24. UV-Vis deprotonation spectra of benzo[c]carbazole in MeCN.

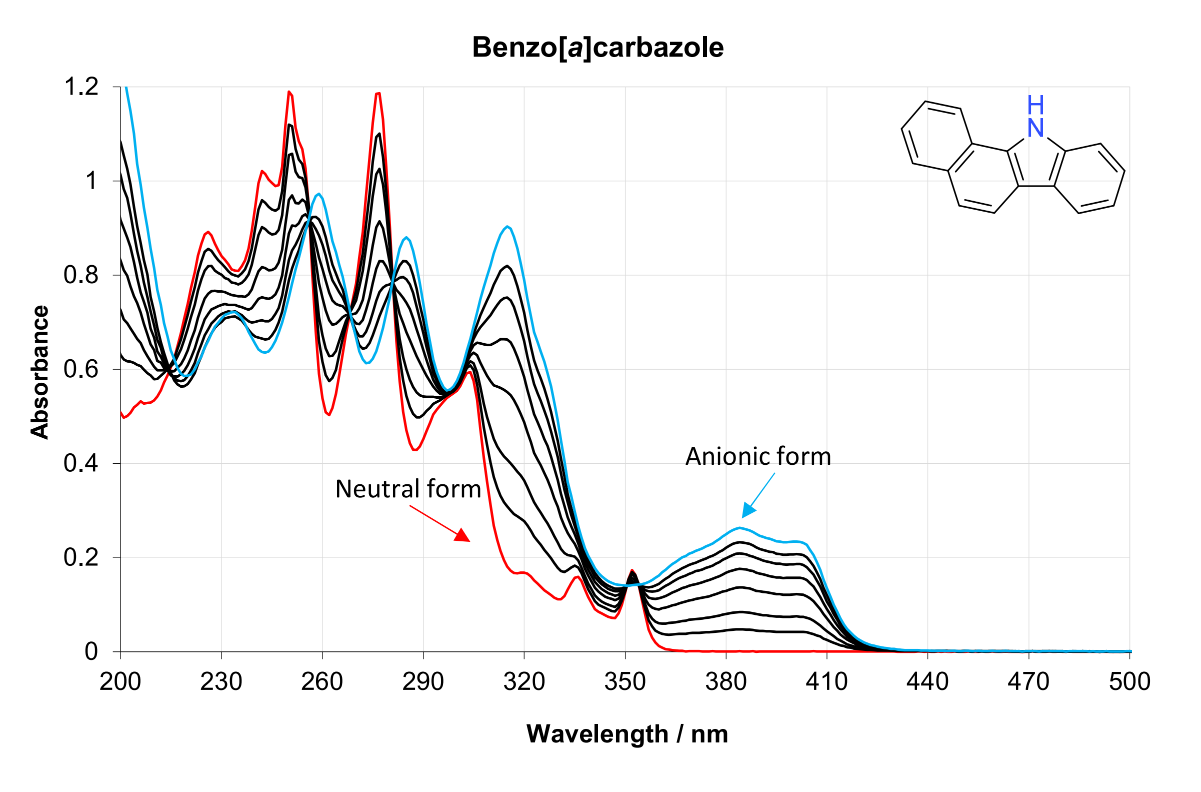

Figure S25. UV-Vis deprotonation spectra of benzo[a]carbazole in MeCN.

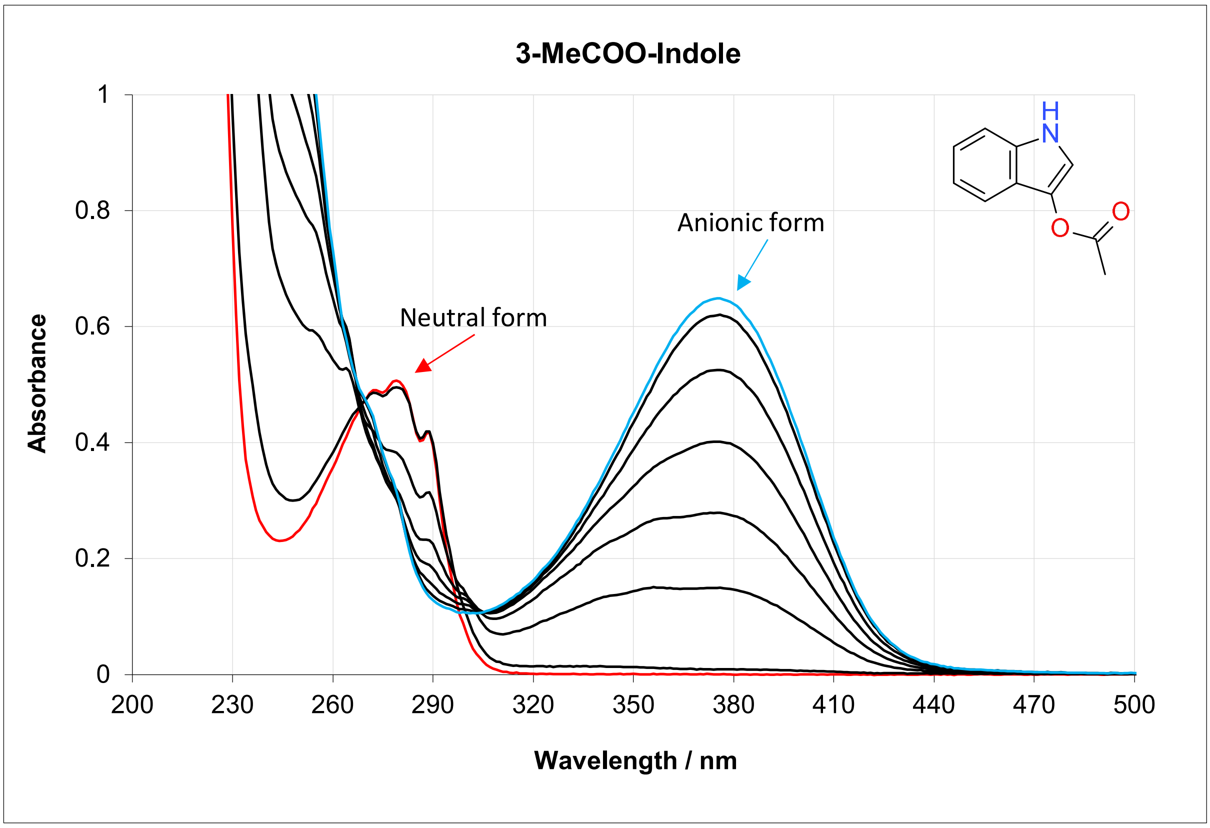

Figure S26. UV-Vis deprotonation spectra of 3-MeCOO-indole in MeCN.

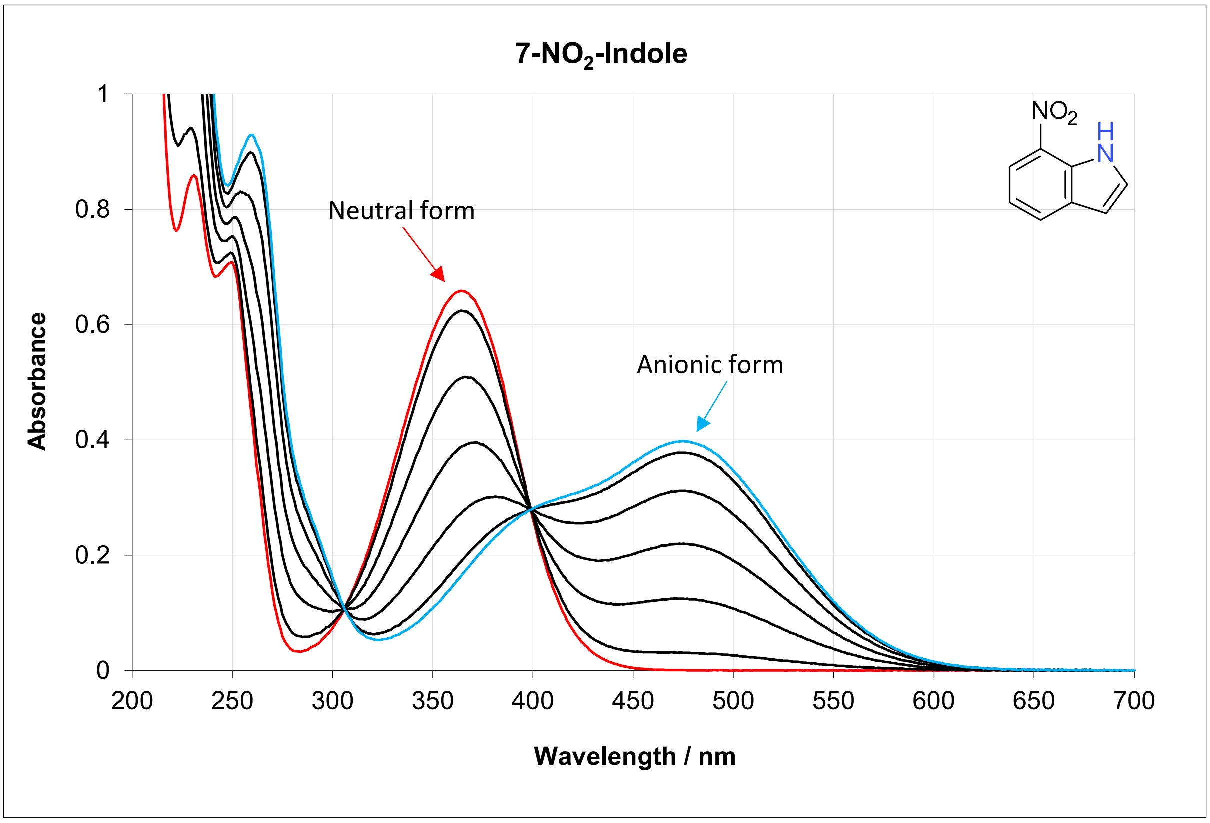

Figure S27. UV-Vis deprotonation spectra of 7-NO_2-indole in MeCN.

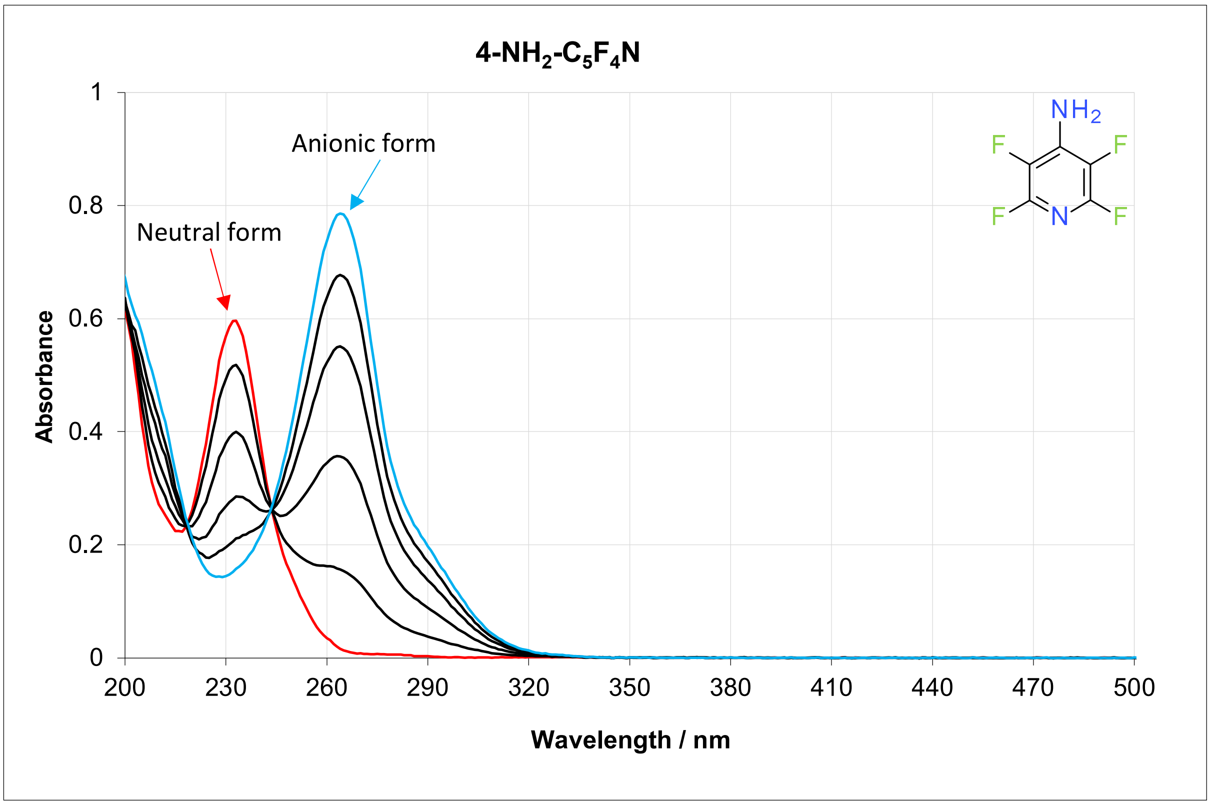

Figure S28. UV-Vis deprotonation spectra of 4-NH$_2$-C$_5$H$_4$N in MeCN.

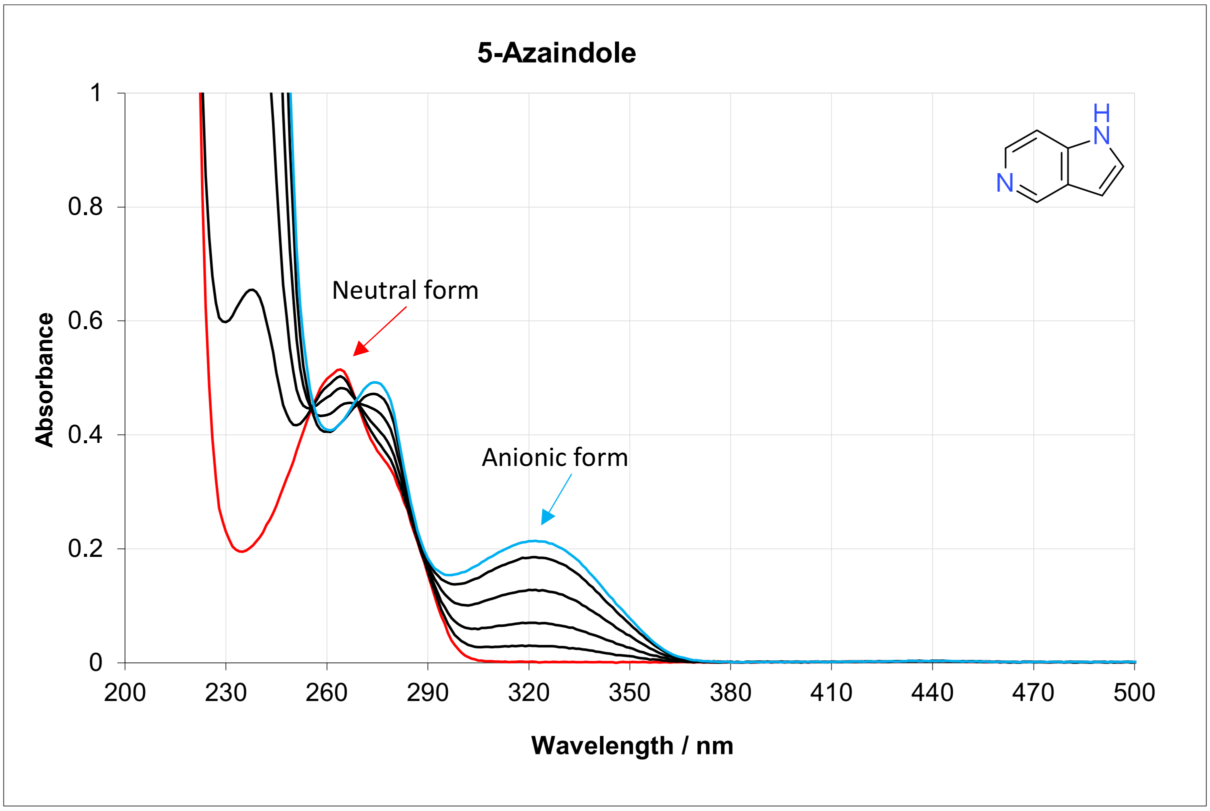

Figure S29. UV-Vis deprotonation spectra of 5-azaindole in MeCN.

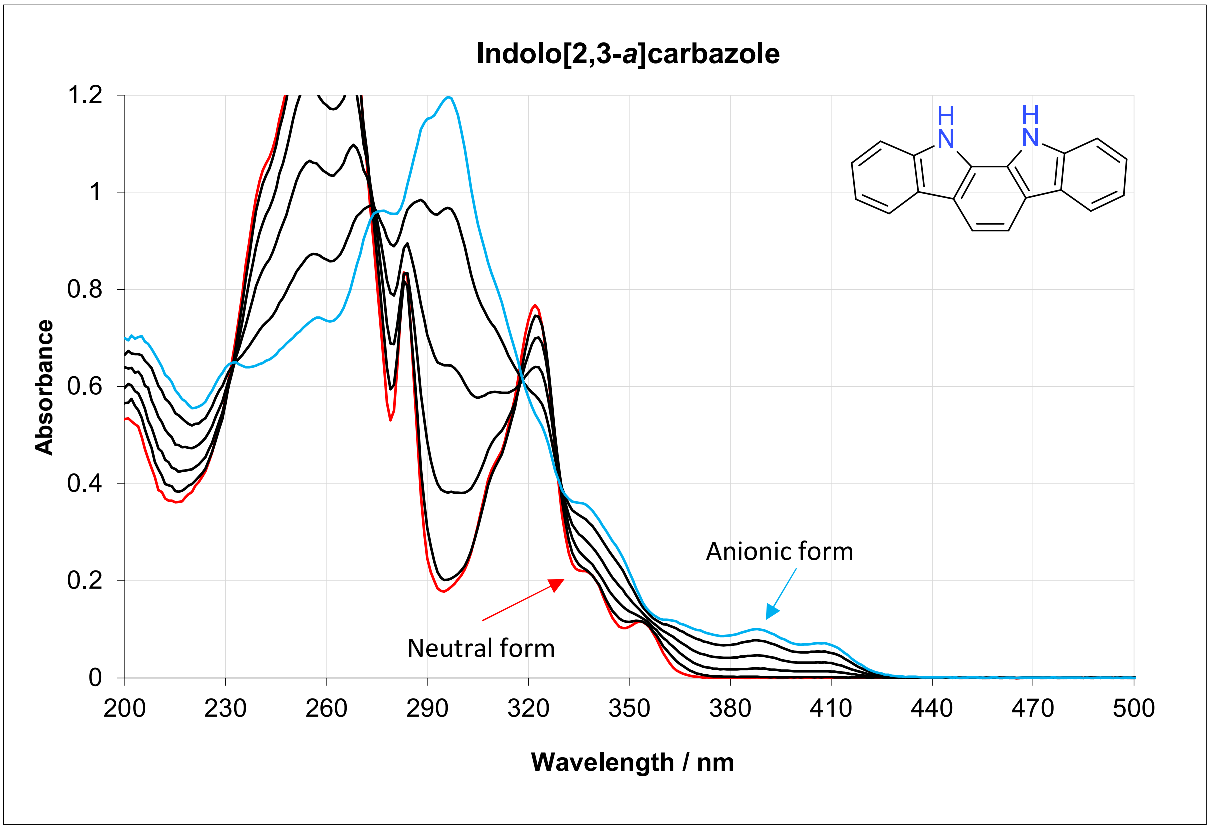

Figure S30. UV-Vis deprotonation spectra of indolo[2,3-a]carbazole in MeCN.

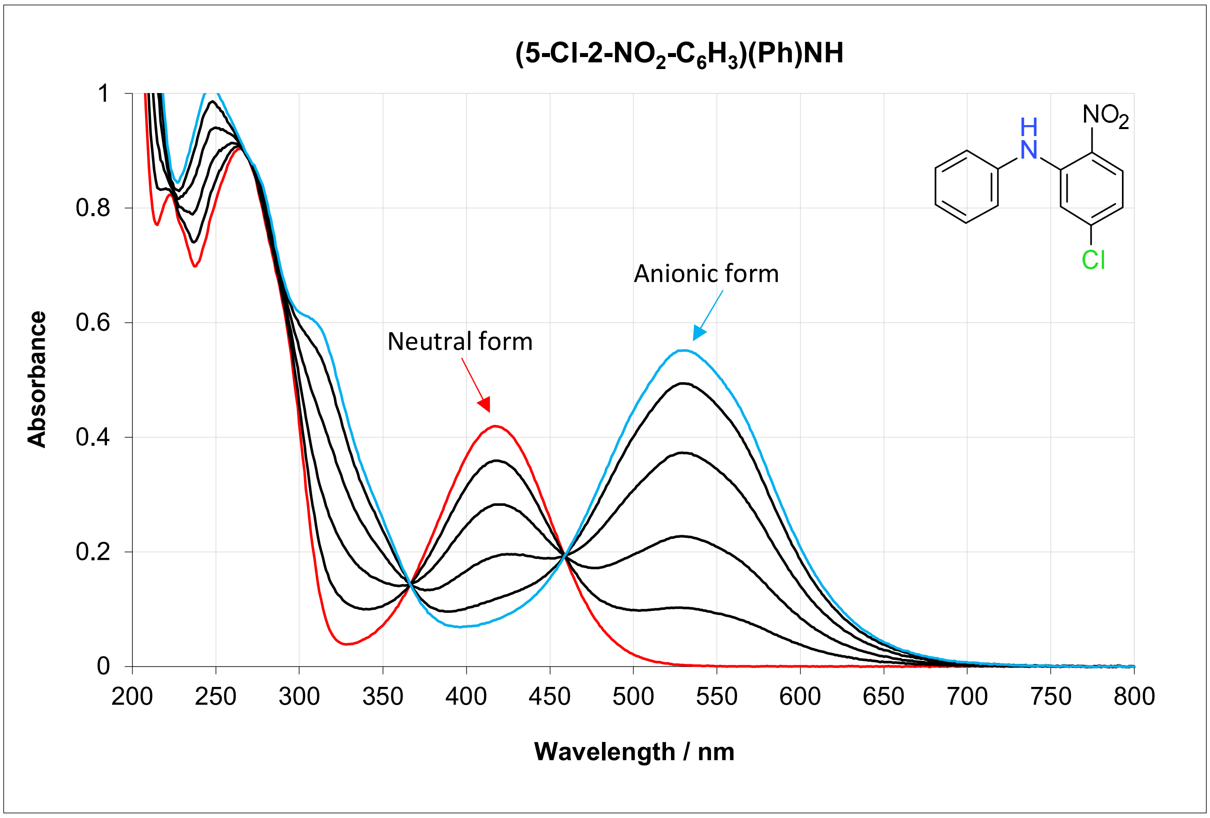

Figure S31. UV-Vis deprotonation spectra of (5-Cl-2-NO$_2$-C$_6$H$_3$)(Ph)NH in MeCN.

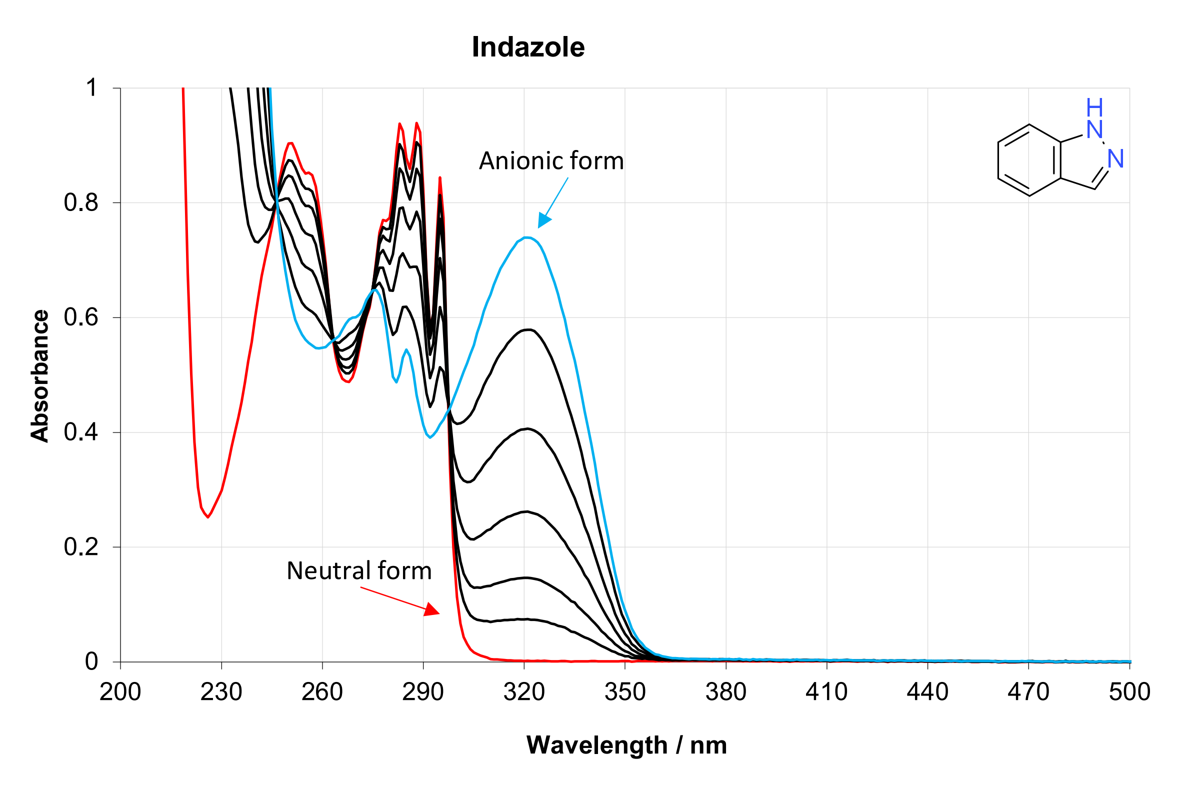

Figure S32. UV-Vis deprotonation spectra of indazole in MeCN.

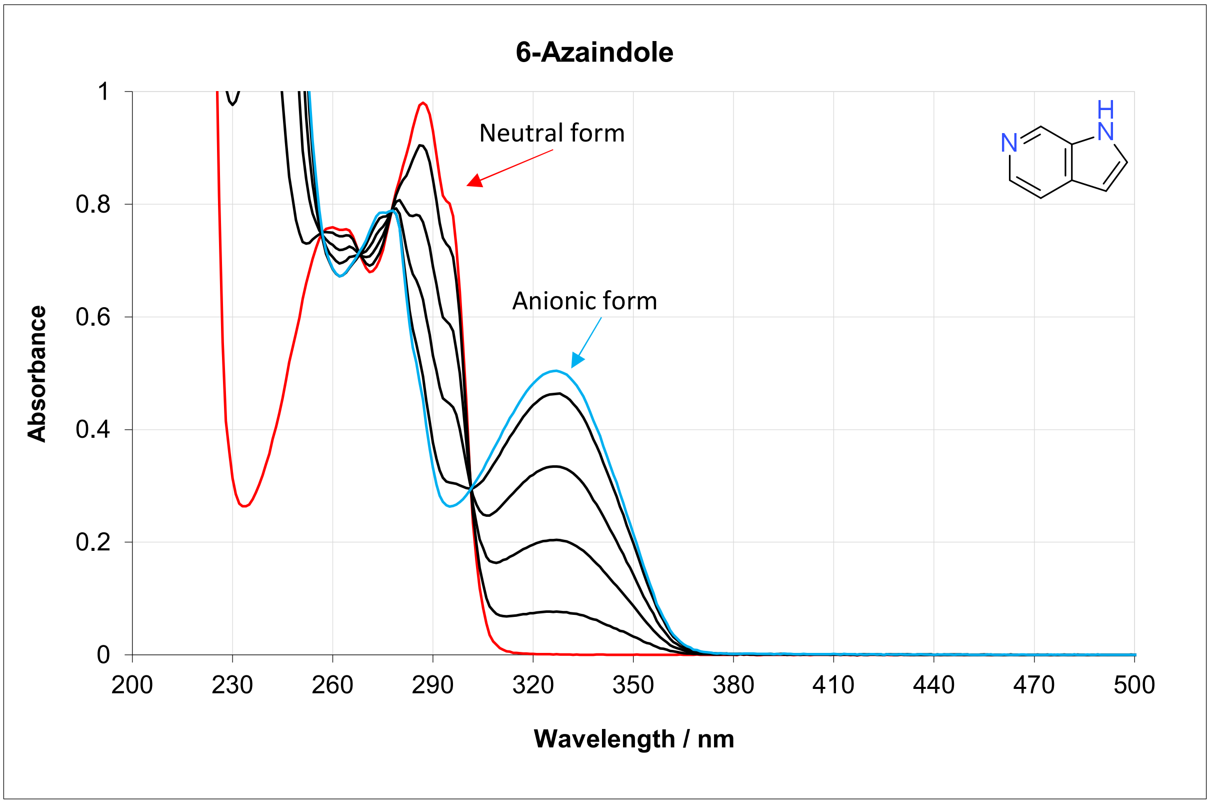

Figure S33. UV-Vis deprotonation spectra of 6-azaindole in MeCN.

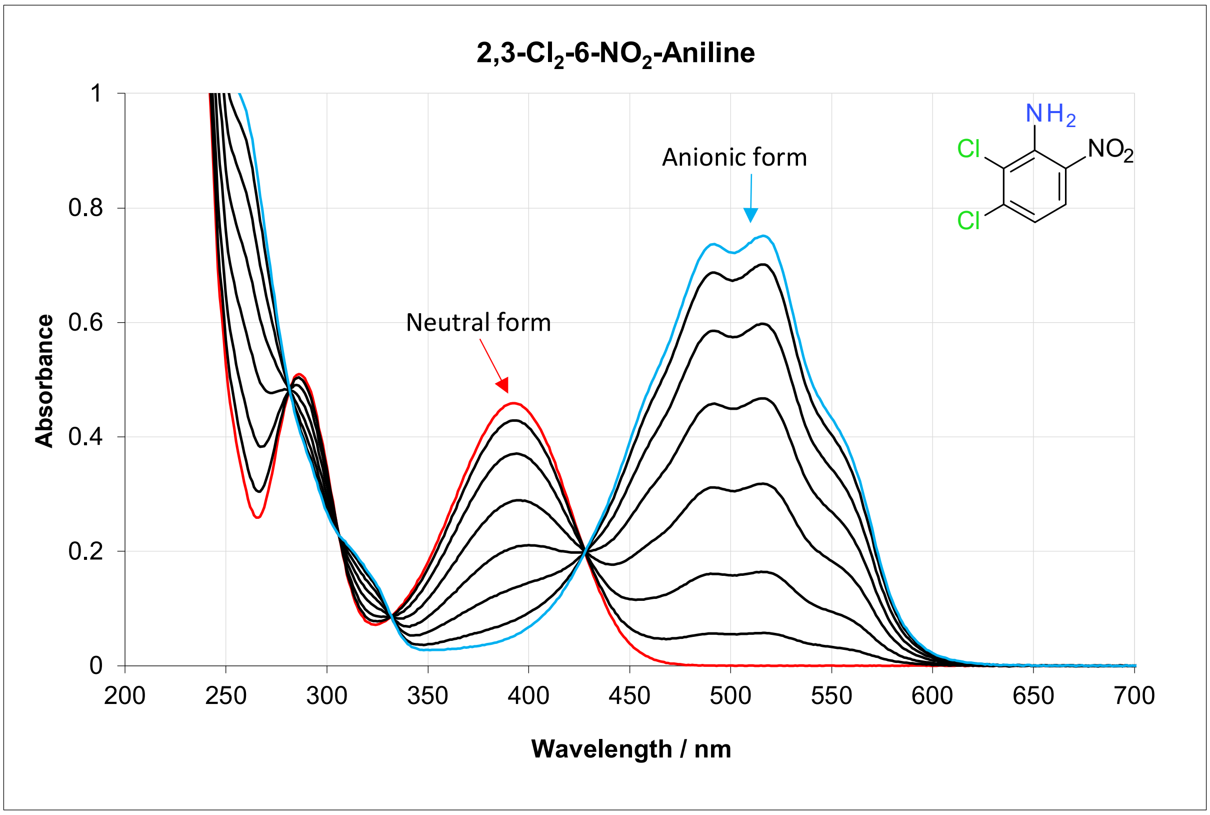

Figure S34. UV-Vis deprotonation spectra of 2,3-Cl_2-6-NO_2-aniline in MeCN.

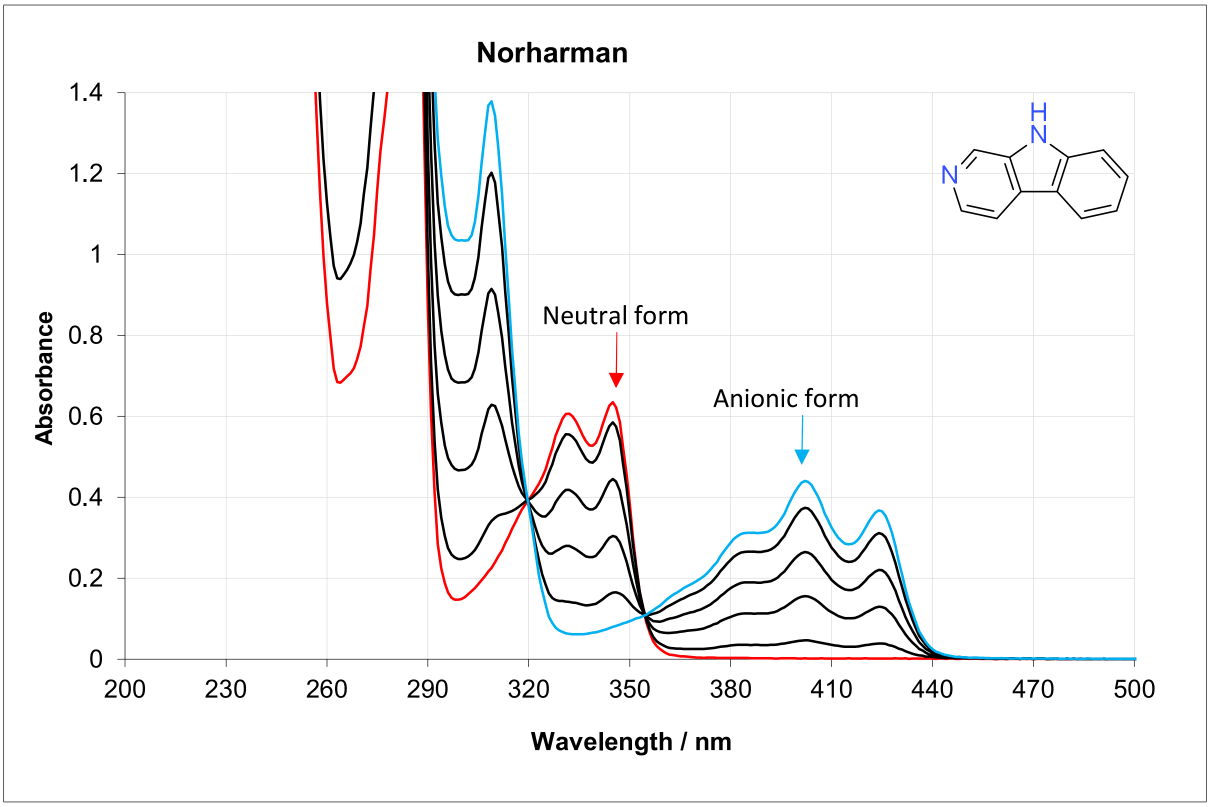

Figure S35. UV-Vis deprotonation spectra of norharman in MeCN.

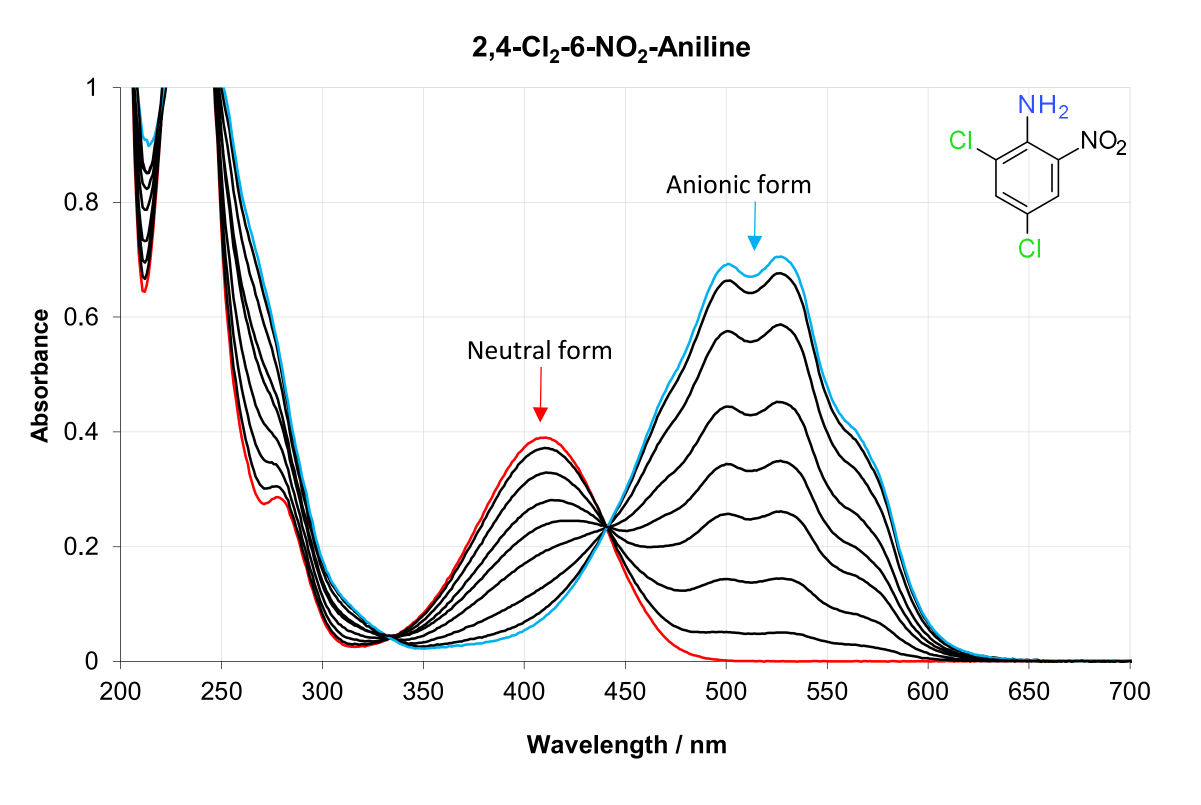

Figure S36. UV-Vis deprotonation spectra of 2,4-Cl_2-6-NO_2-aniline in MeCN.

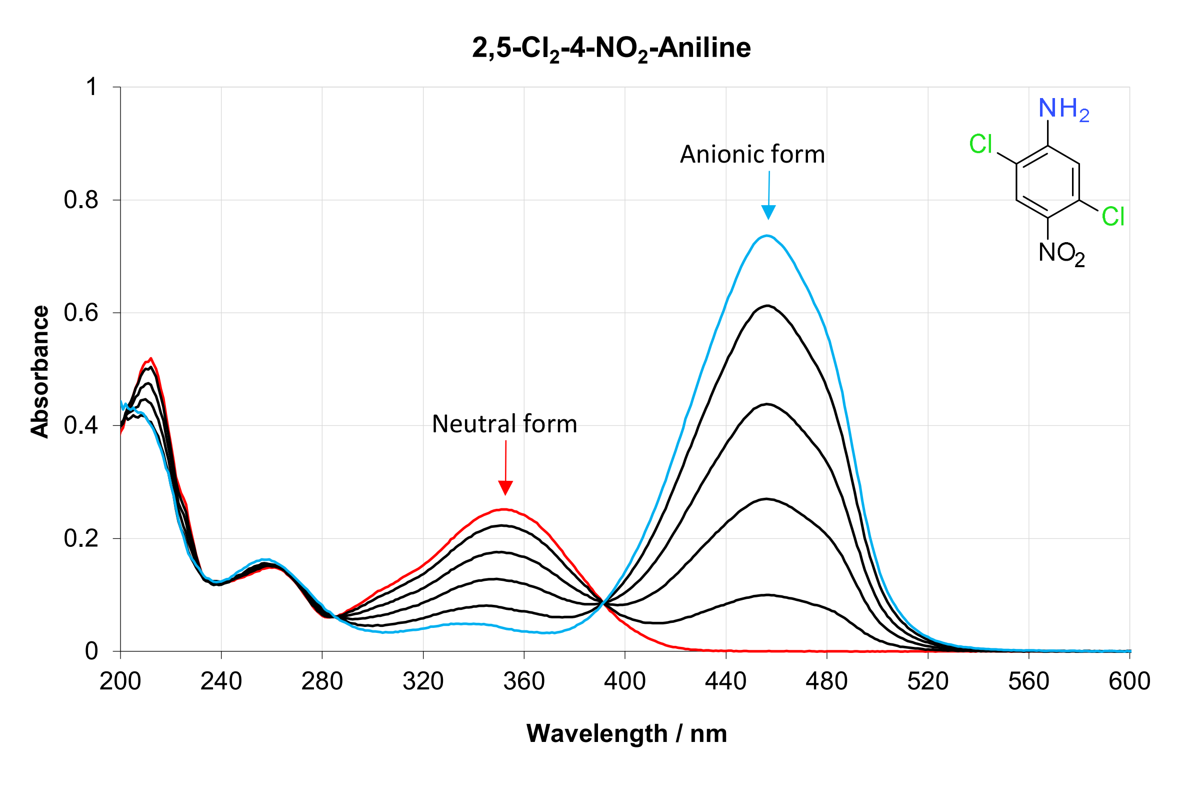

Figure S37. UV-Vis deprotonation spectra of 2,5-Cl_2-4-NO_2-aniline in MeCN.

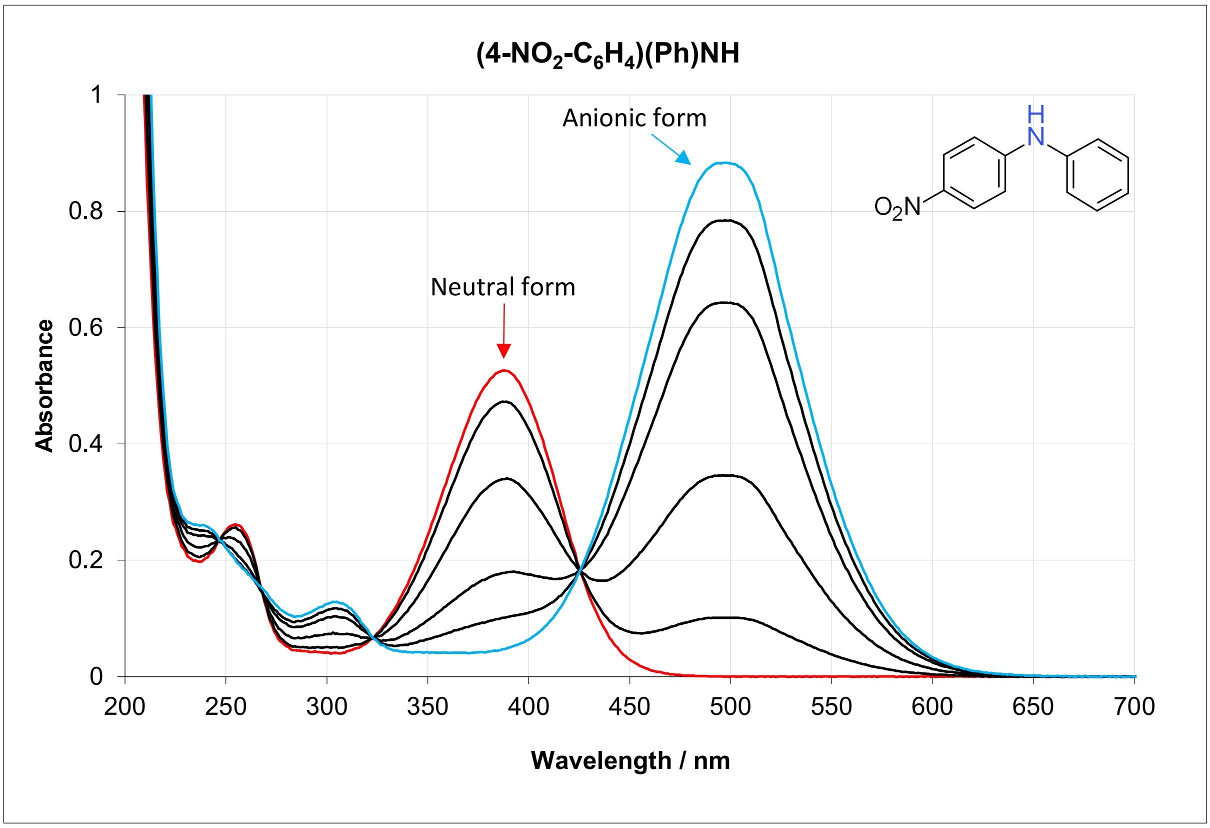

Figure S38. UV-Vis deprotonation spectra of (4-NO_2-C_6H_4)(Ph)NH in MeCN.

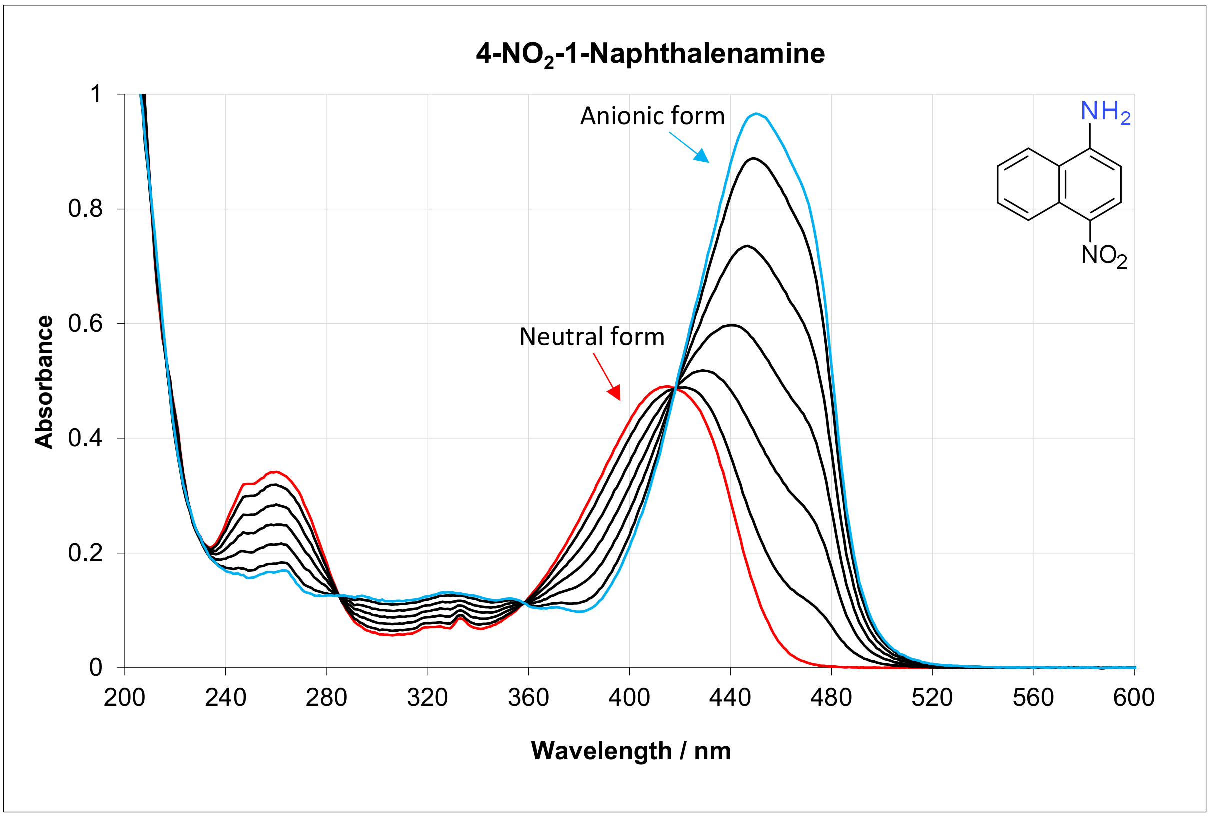

Figure S39. UV-Vis deprotonation spectra of 4-NO_2-1-naphthalenamine in MeCN.

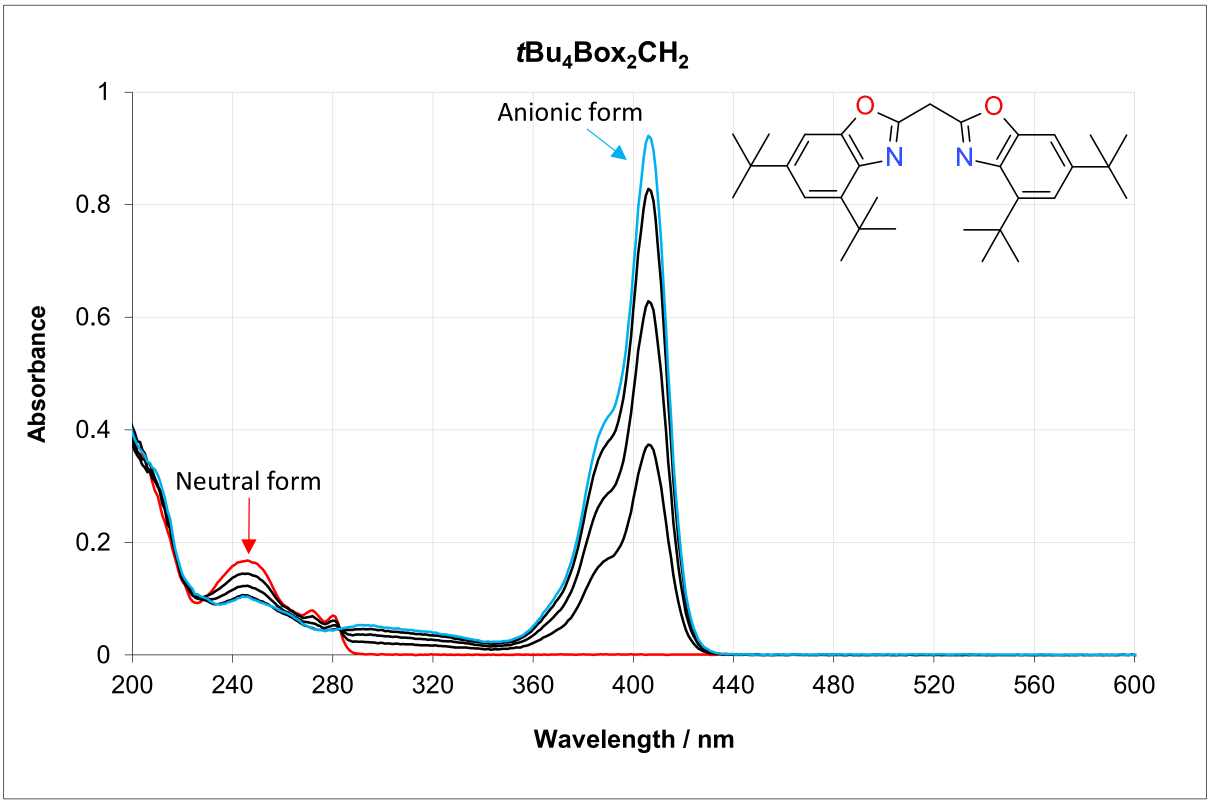

Figure S40. UV-Vis deprotonation spectra of *t*$Bu_4Box_2CH_2$ in MeCN.

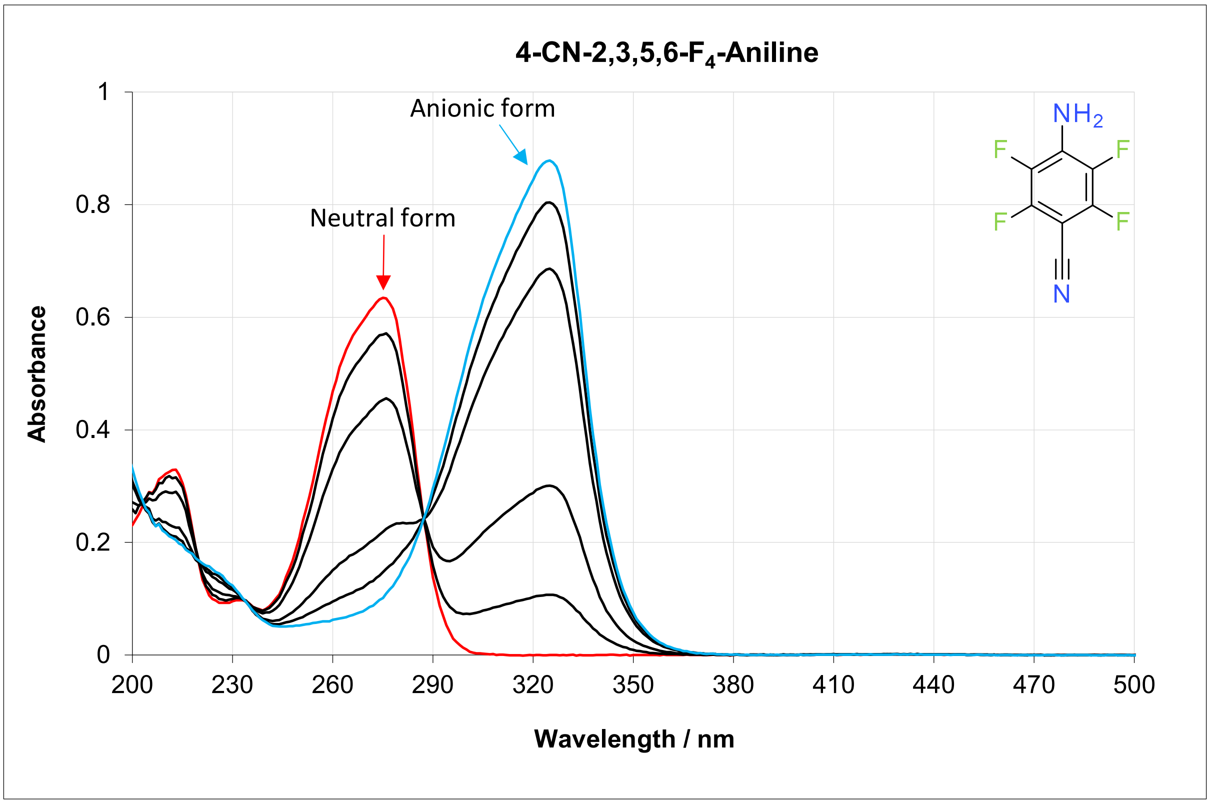

Figure S41. UV-Vis deprotonation spectra of 4-CN-2,3,5,6-F_4-aniline in MeCN.

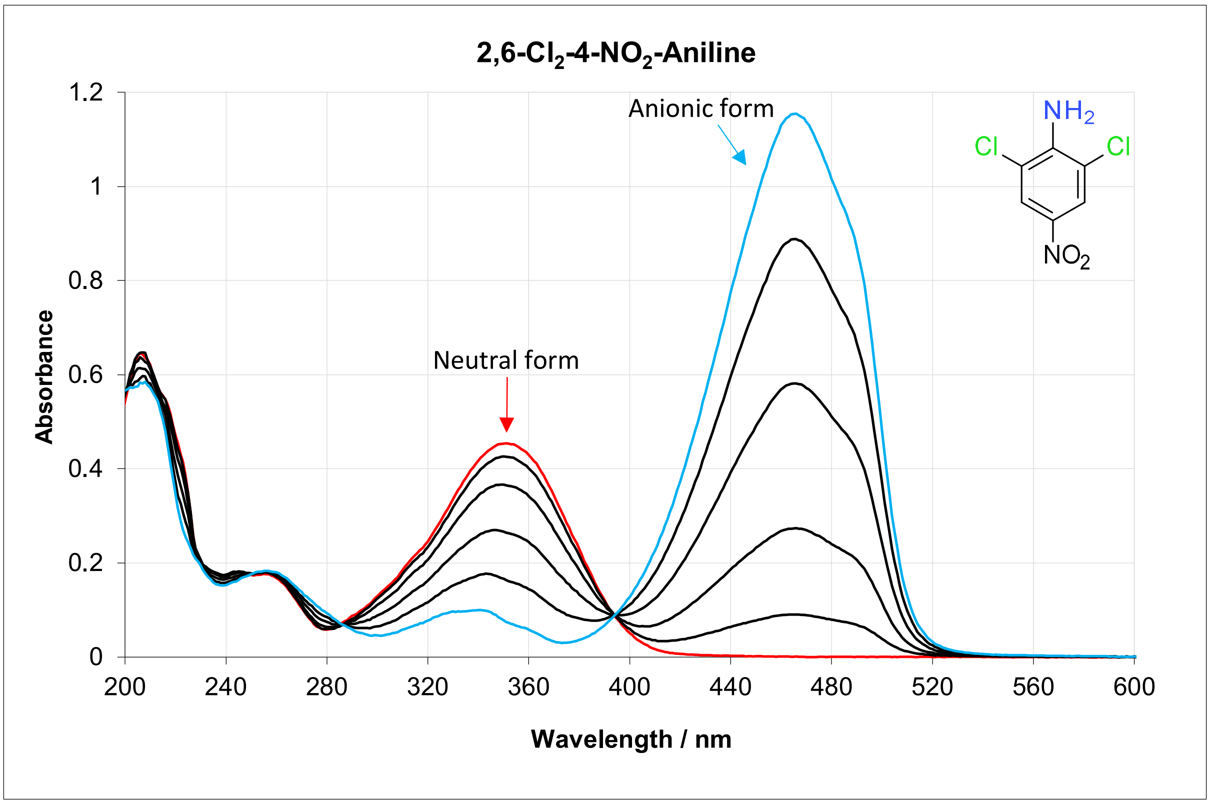

Figure S42. UV-Vis deprotonation spectra of 2,6-Cl_2-4-NO_2-aniline in MeCN.

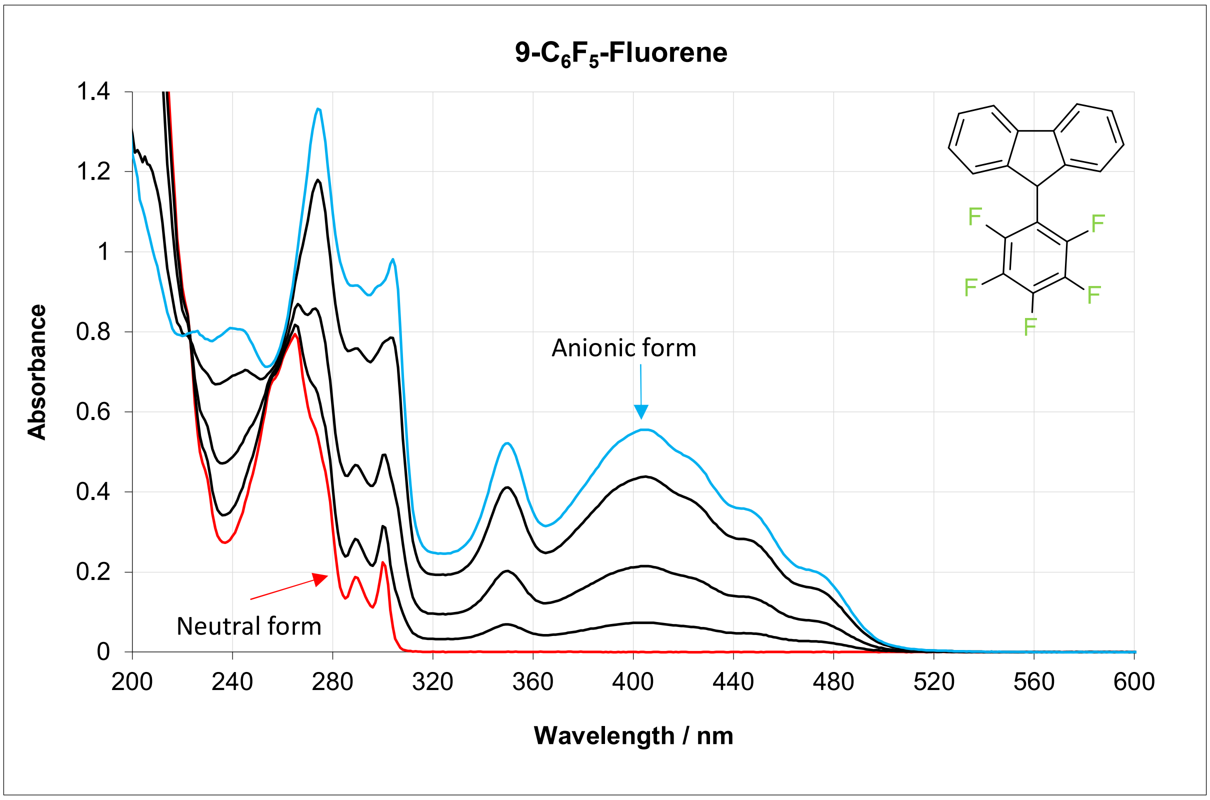

Figure S43. UV-Vis deprotonation spectra of 9-C_6F_5-fluorene in MeCN.

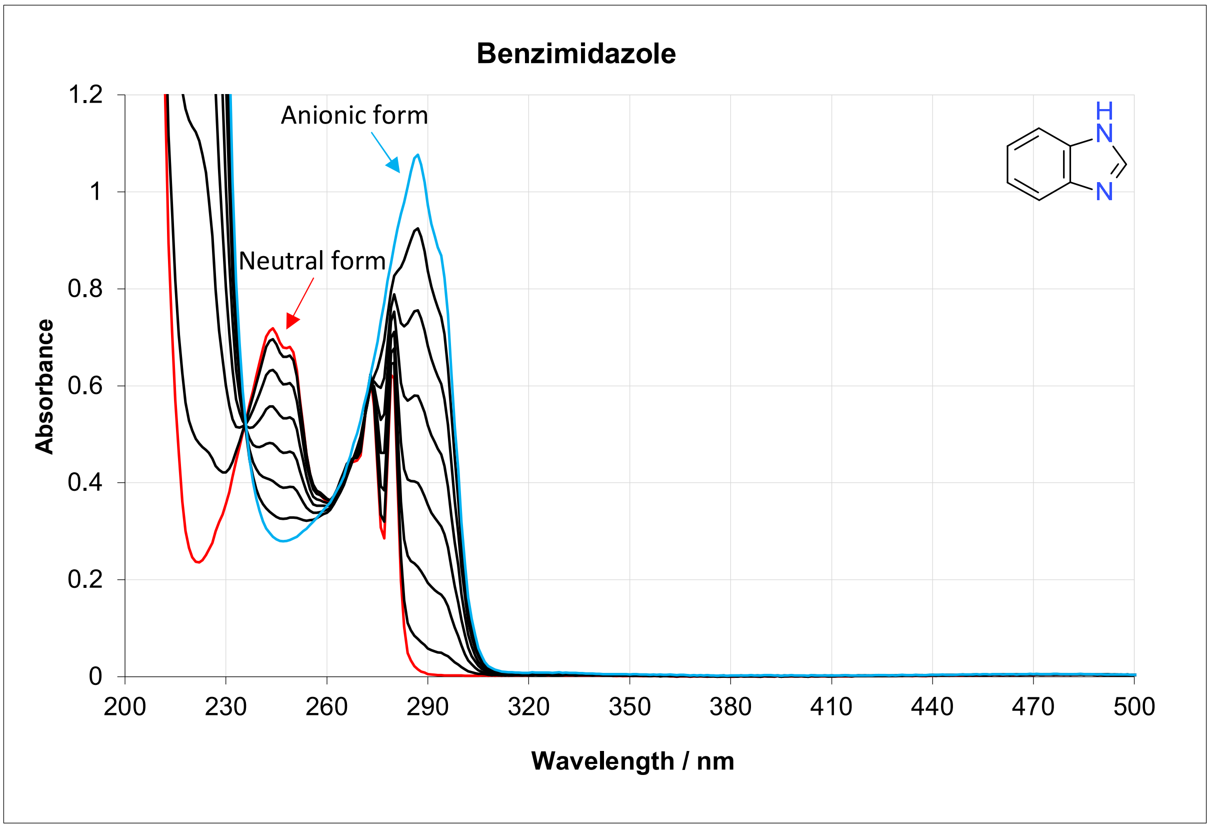

Figure S44. UV-Vis deprotonation spectra of benzimidazole in MeCN.

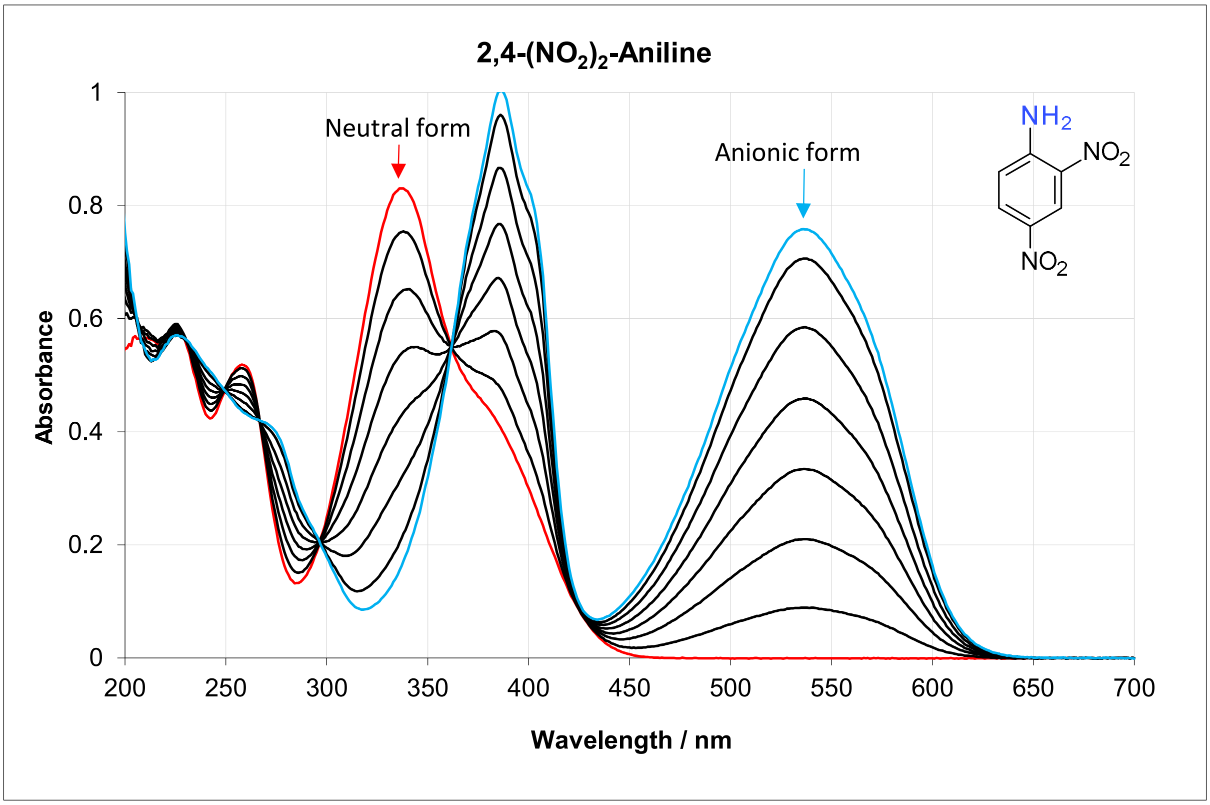

Figure S45. UV-Vis deprotonation spectra of 2,4-$(NO_2)_2$-aniline in MeCN.

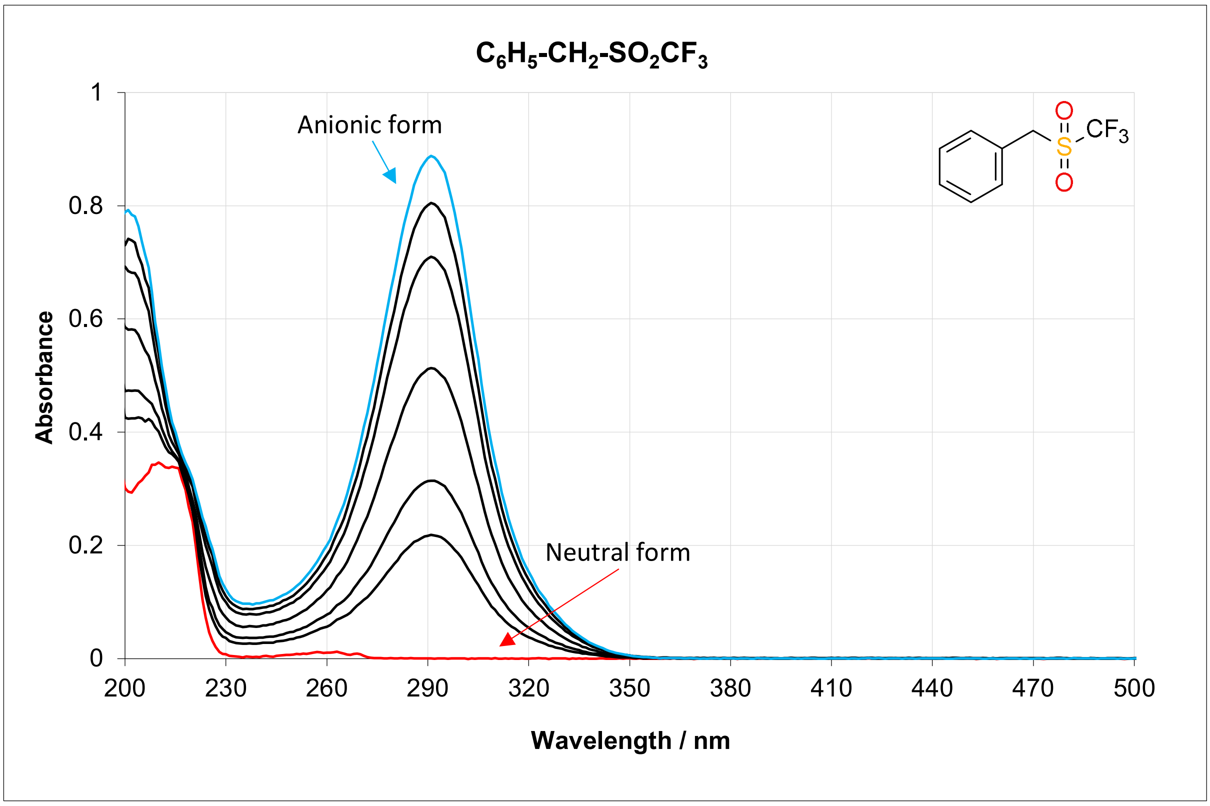

Figure S46. UV-Vis deprotonation spectra of C_6H_5-CH_2-SO_2CF_3 in MeCN.

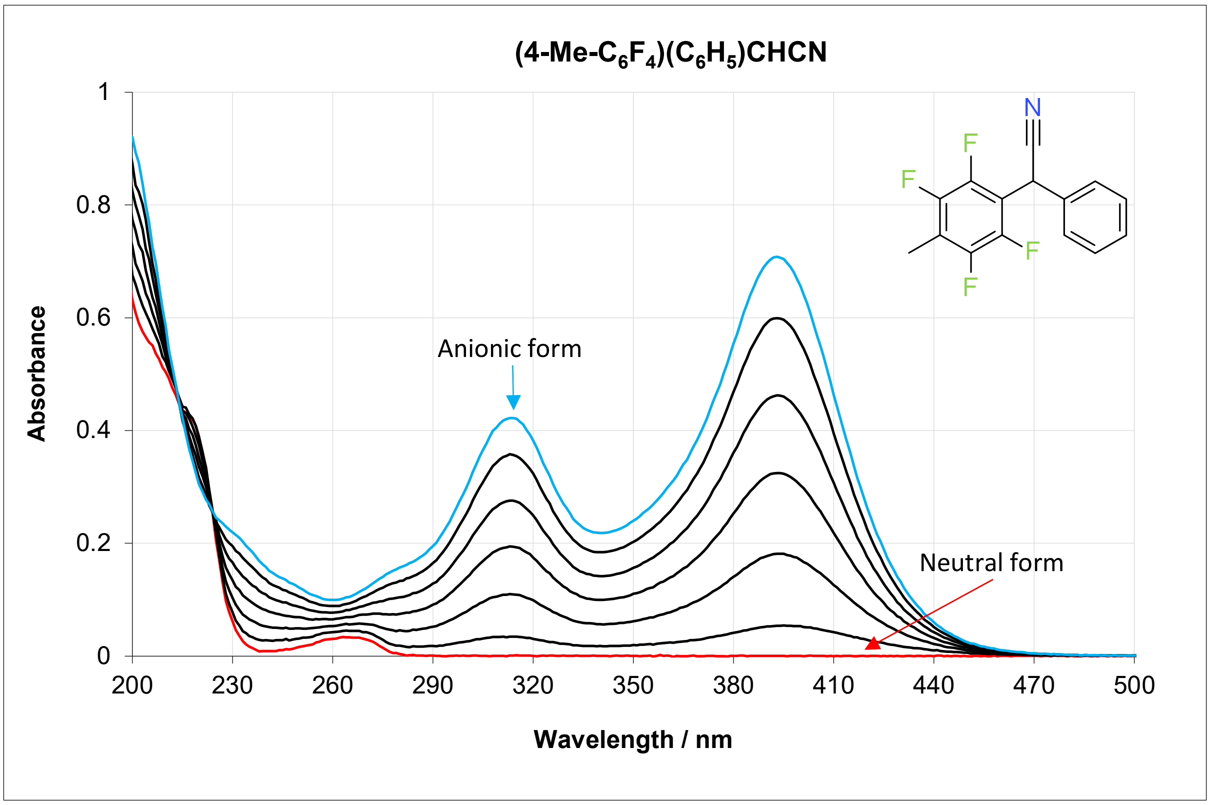

Figure S47. UV-Vis deprotonation spectra of (4-Me-C_6F_4)(C_6H_5) in MeCN.

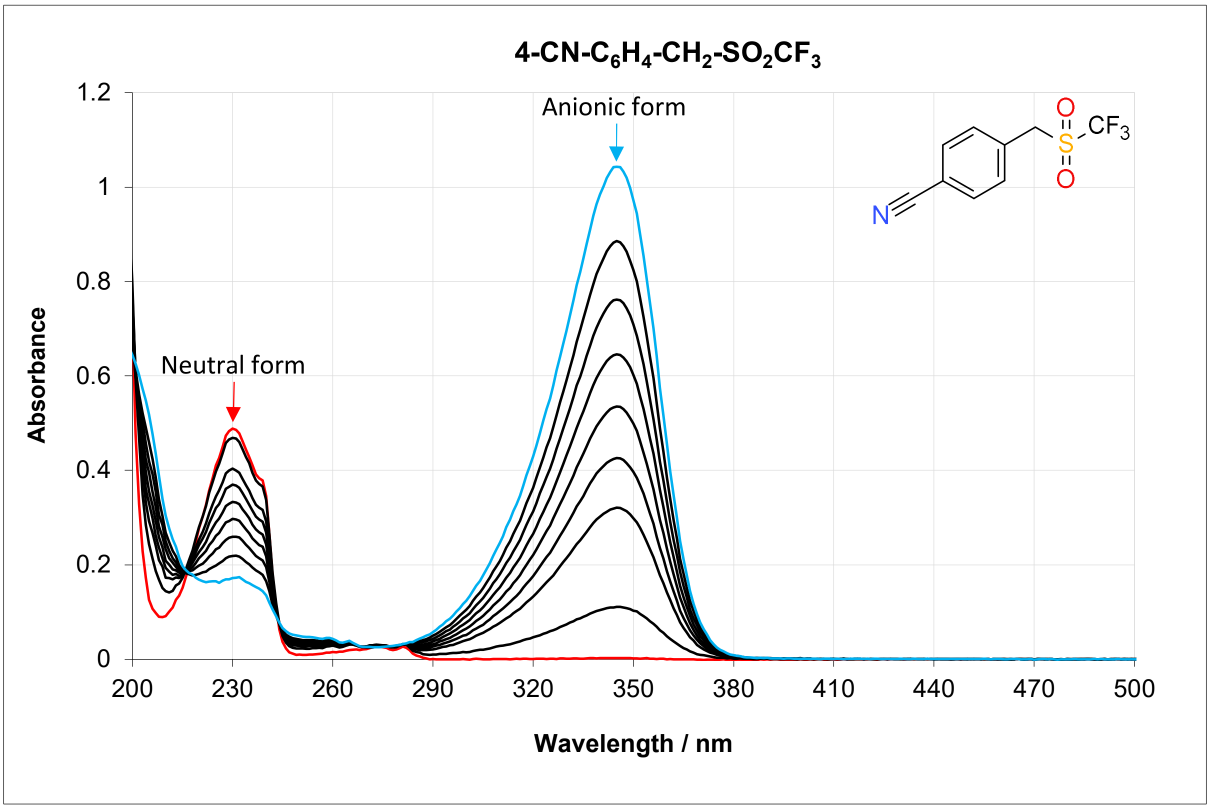

Figure S48. UV-Vis deprotonation spectra of 4-CN-C_6H_4-CH_2-SO_2CF_3 in MeCN.

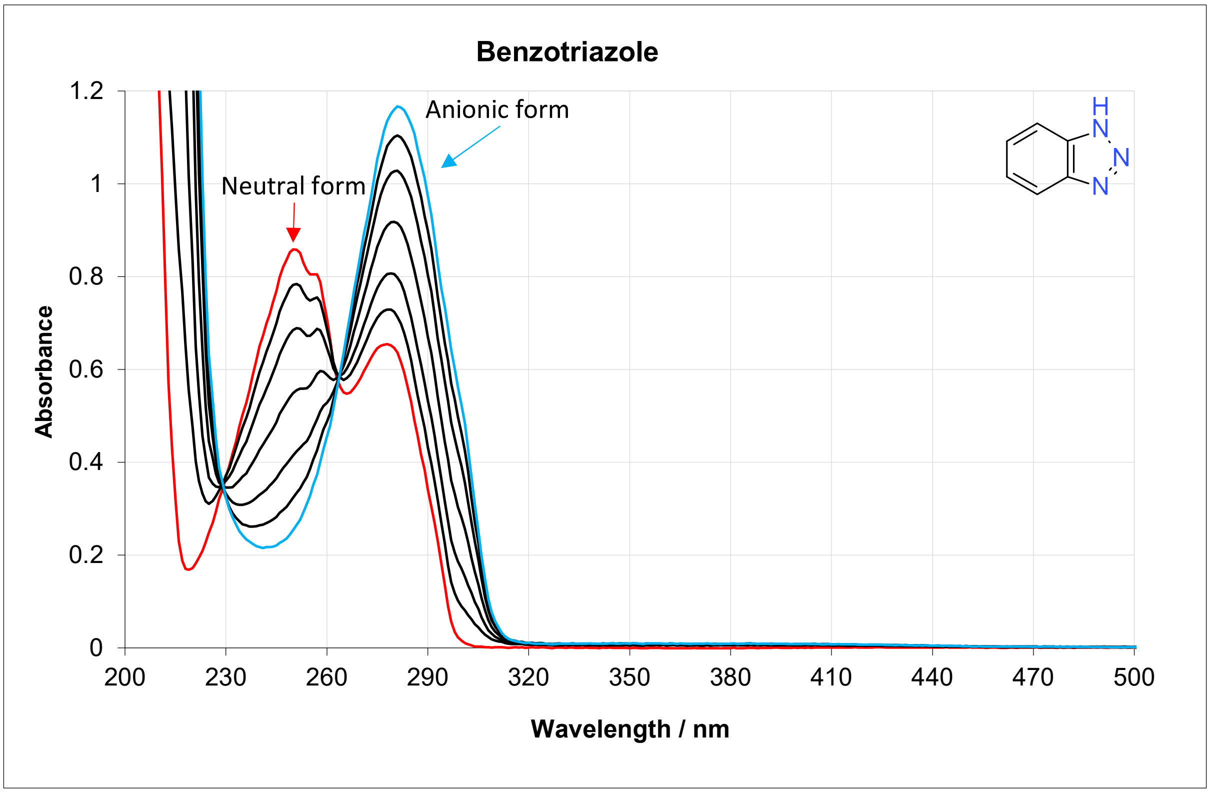

Figure S49. UV-Vis deprotonation spectra of benzotriazole in MeCN.

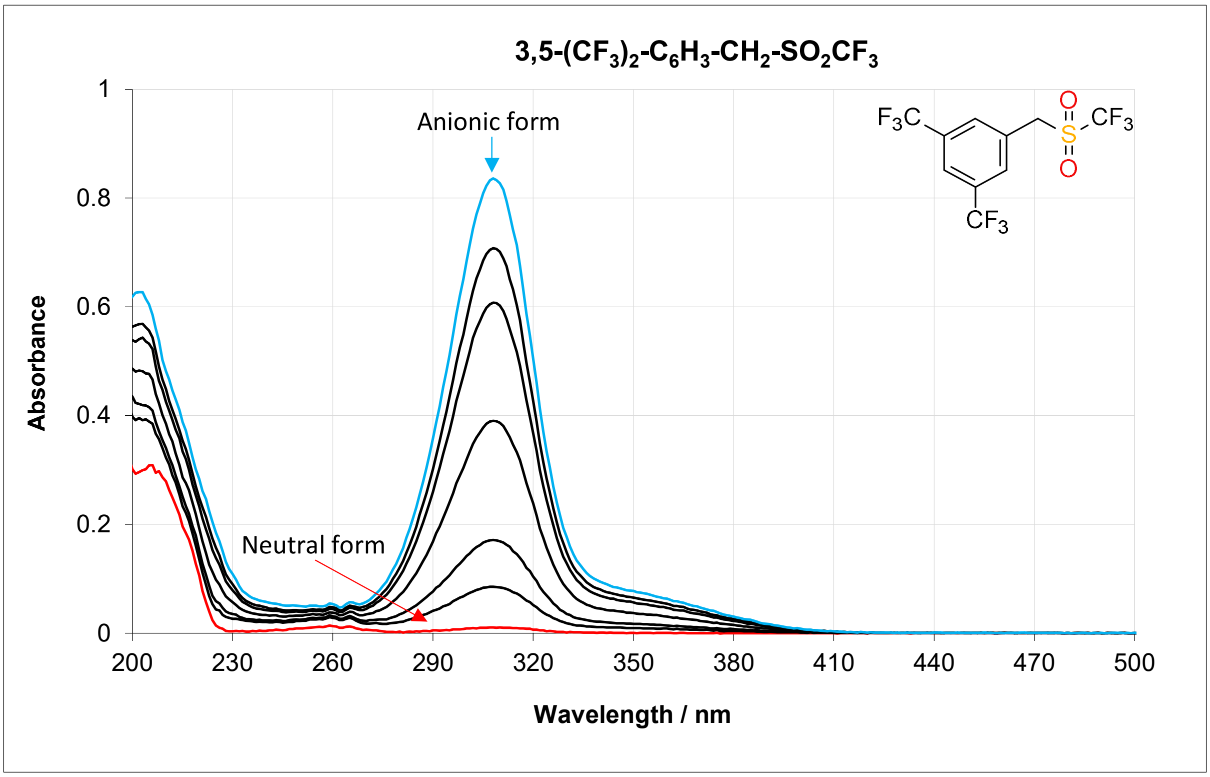

Figure S50. UV-Vis deprotonation spectra of 3,5-$(CF_3)_2$-C_6H_3-CH_2-SO_2CF_3 in MeCN.

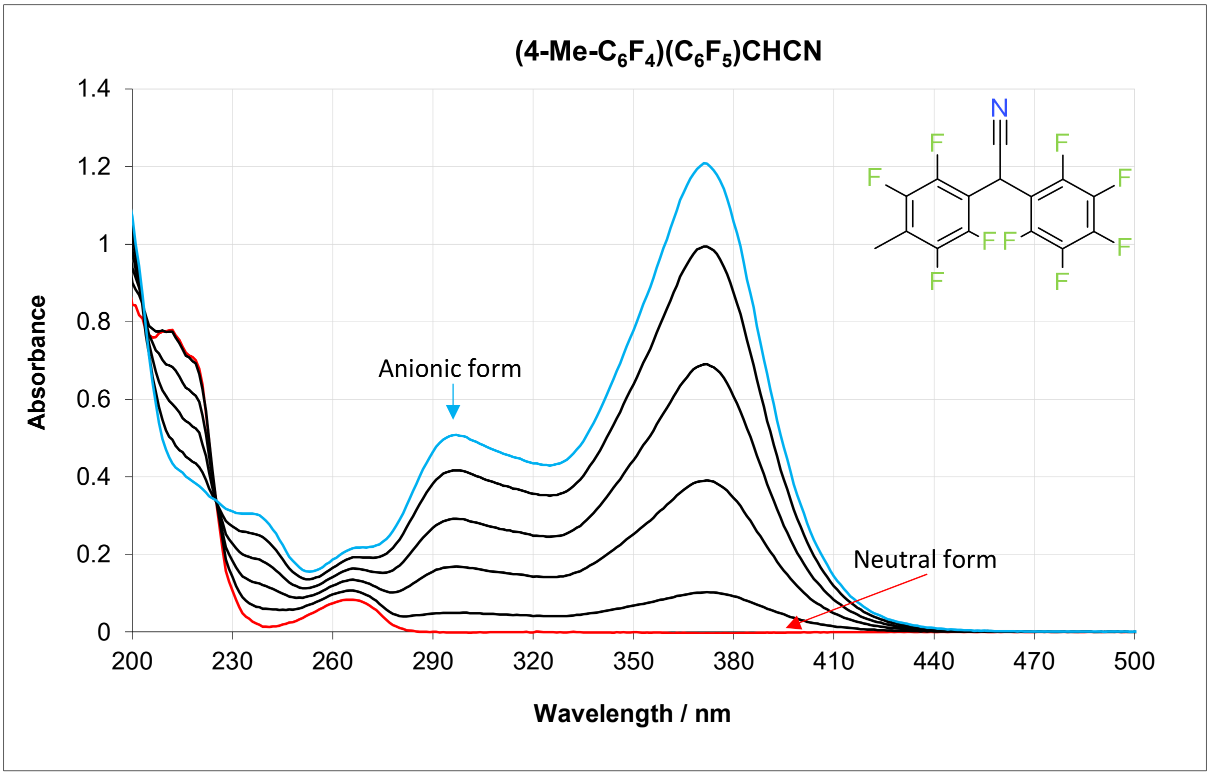

Figure S51. UV-Vis deprotonation spectra of (4-Me-C_6F_4)(C_6F_5) in MeCN.
